# Personalized RNA neoantigen vaccines stimulate T cells in pancreatic cancer

**DOI:** 10.1038/s41586-023-06063-y

**Published:** 2023-05-10

**Authors:** Luis A. Rojas, Zachary Sethna, Kevin C. Soares, Cristina Olcese, Nan Pang, Erin Patterson, Jayon Lihm, Nicholas Ceglia, Pablo Guasp, Alexander Chu, Rebecca Yu, Adrienne Kaya Chandra, Theresa Waters, Jennifer Ruan, Masataka Amisaki, Abderezak Zebboudj, Zagaa Odgerel, George Payne, Evelyna Derhovanessian, Felicitas Müller, Ina Rhee, Mahesh Yadav, Anton Dobrin, Michel Sadelain, Marta Łuksza, Noah Cohen, Laura Tang, Olca Basturk, Mithat Gönen, Seth Katz, Richard Kinh Do, Andrew S. Epstein, Parisa Momtaz, Wungki Park, Ryan Sugarman, Anna M. Varghese, Elizabeth Won, Avni Desai, Alice C. Wei, Michael I. D’Angelica, T. Peter Kingham, Ira Mellman, Taha Merghoub, Jedd D. Wolchok, Ugur Sahin, Özlem Türeci, Benjamin D. Greenbaum, William R. Jarnagin, Jeffrey Drebin, Eileen M. O’Reilly, Vinod P. Balachandran

**Affiliations:** 1 Immuno-Oncology Service, Human Oncology and Pathogenesis Program, Memorial Sloan Kettering Cancer Center, New York, NY USA; 2 Hepatopancreatobiliary Service, Department of Surgery, Memorial Sloan Kettering Cancer Center, New York, NY USA; 3 David M. Rubenstein Center for Pancreatic Cancer Research, Memorial Sloan Kettering Cancer Center, New York, NY USA; 4 Computational Oncology Service, Department of Epidemiology and Biostatistics, Memorial Sloan Kettering Cancer Center, New York, NY USA; 5 BioNTech, Mainz, Germany; 6 Genentech, San Francisco, CA USA; 7 Center for Cell Engineering, Memorial Sloan Kettering Cancer Center, New York, NY USA; 8 Immunology Program, Sloan Kettering Institute, Memorial Sloan Kettering Cancer Center, New York, NY USA; 9 Department of Oncological Sciences, Tisch Cancer Institute, Icahn School of Medicine at Mount Sinai, New York, NY USA; 10 Department of Surgery, Icahn School of Medicine at Mount Sinai, New York, NY USA; 11 Department of Pathology, Memorial Sloan Kettering Cancer Center, New York, NY USA; 12 Department of Epidemiology and Biostatistics, Memorial Sloan Kettering Cancer Center, New York, NY USA; 13 Department of Radiology, Memorial Sloan Kettering Cancer Center, New York, NY USA; 14 Department of Medicine, Memorial Sloan Kettering Cancer Center, New York, NY USA; 15 Meyer Cancer Center, Weill Cornell Medicine, Weill Cornell Medical College, New York, NY USA; 16 HI-TRON, Helmholtz Institute for Translational Oncology, Mainz, Germany; 17 Physiology, Biophysics and Systems Biology, Weill Cornell Medicine, Weill Cornell Medical College, New York, NY USA

**Keywords:** Cancer immunotherapy, Pancreatic cancer

## Abstract

Pancreatic ductal adenocarcinoma (PDAC) is lethal in 88% of patients^1^, yet harbours mutation-derived T cell neoantigens that are suitable for vaccines ^2,3^. Here in a phase I trial of adjuvant autogene cevumeran, an individualized neoantigen vaccine based on uridine mRNA–lipoplex nanoparticles, we synthesized mRNA neoantigen vaccines in real time from surgically resected PDAC tumours. After surgery, we sequentially administered atezolizumab (an anti-PD-L1 immunotherapy), autogene cevumeran (a maximum of 20 neoantigens per patient) and a modified version of a four-drug chemotherapy regimen (mFOLFIRINOX, comprising folinic acid, fluorouracil, irinotecan and oxaliplatin). The end points included vaccine-induced neoantigen-specific T cells by high-threshold assays, 18-month recurrence-free survival and oncologic feasibility. We treated 16 patients with atezolizumab and autogene cevumeran, then 15 patients with mFOLFIRINOX. Autogene cevumeran was administered within 3 days of benchmarked times, was tolerable and induced de novo high-magnitude neoantigen-specific T cells in 8 out of 16 patients, with half targeting more than one vaccine neoantigen. Using a new mathematical strategy to track T cell clones (CloneTrack) and functional assays, we found that vaccine-expanded T cells comprised up to 10% of all blood T cells, re-expanded with a vaccine booster and included long-lived polyfunctional neoantigen-specific effector CD8^+^ T cells. At 18-month median follow-up, patients with vaccine-expanded T cells (responders) had a longer median recurrence-free survival (not reached) compared with patients without vaccine-expanded T cells (non-responders; 13.4 months, *P* = 0.003). Differences in the immune fitness of the patients did not confound this correlation, as responders and non-responders mounted equivalent immunity to a concurrent unrelated mRNA vaccine against SARS-CoV-2. Thus, adjuvant atezolizumab, autogene cevumeran and mFOLFIRINOX induces substantial T cell activity that may correlate with delayed PDAC recurrence.

## Abstract (web summary)

A phase I clinical trial of an adjuvant personalized mRNA neoantigen vaccine, autogene cevumeran, in patients with pancreatic ductal carcinoma demonstrates that the vaccine can induce T cell activity that may correlate with delayed recurrence of disease.

## Main

PDAC is the third leading cause of cancer death in the United States^4^ and the seventh worldwide^5^. With an increasing incidence^6^, and a survival rate of 12%^1^ that has remained largely stagnant for nearly 60 years^1^, PDAC is projected to cause even greater global cancer deaths by 2025 (refs. ^6,7^). Surgery is the only curative treatment for PDAC. Yet, despite surgery, nearly 90% of patients have disease recurrence at a median of 7–9 months^8,9^, and the 5-year overall survival (OS) is only 8–10%^8,9^. Although adjuvant multiagent chemotherapies delay recurrence and are standard of care in surgically resected PDAC, nearly 80% of patients have disease recurrence at around 14 months^4^, and their 5-year OS is <30%^10^. Radiation, biologics and targeted therapies are also ineffective^4^.

PDACs are almost completely insensitive (<5% response rate^11,12^) to immune checkpoint inhibitors. This insensitivity is partially attributed to the fact that PDACs have a low mutation rate that generates few neoantigens^12^, mutation-generated proteins absent from healthy tissues that mark cancers as foreign to T cells, thus potenially rendering PDACs weakly antigenic with few infiltrating T cells. However, recent observations have shown that most PDACs in fact harbour more neoantigens^2,3,13^ than previously predicted^14^. Furthermore, studies of long-term survivors of PDAC^2,3^ have revealed that neoantigens may stimulate T cells in PDAC. Primary tumours enriched in immunogenic neoantigens also harbour around 12-fold higher densities of activated CD8^+^ T cells, which correlates with delayed disease recurrence and longer patient survival. Thus, as most PDACs harbour neoantigens with the potential to stimulate T cells, strategies to deliver neoantigens may induce neoantigen-specific T cells and affect patient outcomes.

Based on the observation that long-term survivors of PDAC mount spontaneous T cell responses against tumour-specific neoantigens not shared among patients^2,3^, we tested whether adjuvant individualized vaccines can stimulate neoantigen-specific T cells and provide clinical benefit in patients with surgically resected PDAC. Therapeutic mRNA vaccine technology has facilitated the rapid delivery of individualized neoantigen vaccines fully integrated into a routine oncologic workflow^15^. Moreover, mRNA can be rapidly manufactured as individualized vaccines with multiple neoantigens^16^, can activate antigen-presenting cells^17–20^ and can be efficiently delivered using newly developed clinical-stage formulations^21^. Therefore, we hypothesized that an effective individualized mRNA vaccine would induce neoantigen-specific T cells in PDAC, eliminate micrometastases and delay recurrence.

**Fig. 1:**
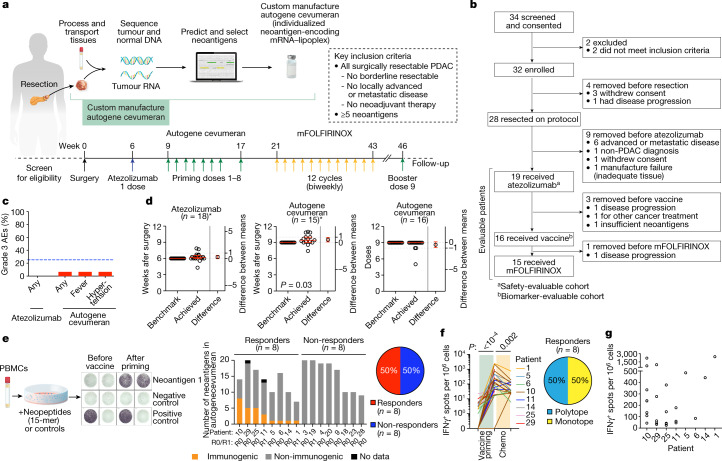
Individualized mRNA neoantigen vaccines are safe, feasible and immunogenic in patients with PDAC. **a**,**b**, Trial design (**a**) and consolidated standards of reporting trials diagram (**b**). **c**, Percentage of grade 3 AEs attributable to atezolizumab and autogene cevumeran (vaccine) in atezolizumab (*n* = 19) and vaccine (*n* = 16) safety-evaluable patients. Blue line indicates the study-defined safety threshold (25%). **d**, Achieved and benchmarked times to atezolizumab (left) and first vaccine dose (middle), and number of vaccine doses (right). Red line indicates the median, error bars are 95% confidence intervals and dotted lines the zone of clinical indifference. Asterisks indicate patients on study-specified treatment sequence. **e**–**g**, PBMCs collected after atezolizumab and before vaccine administration, 1–3 weeks after vaccine priming, and 5–6 weeks after mFOLFIRINOX were analysed for IFNγ^+^ T cells specific to all individual vaccine neoantigens by ex vivo IFNγ ELISpot in *n* = 16 patients in the biomarker-evaluable cohort. Patients were classified as responders if ELISpot detected IFNγ^+^ T cell induction against at least one vaccine neoantigen. **e**, Left, schematic and representative image of ex vivo IFNγ ELISpot. Middle, Number of vaccine neoantigens per patient that induced IFNγ^+^ T cells in PBMCs collected after vaccine priming. R0/R1 indicates the surgical margin status. For patient 25, 2 out of 5 ELISpot responses were detected against 2 neoantigen pools (pool 1 with 2 neoantigens, pool 2 with 5 neoantigens). Right, Proportion of vaccine responders and non-responders. **f**,**g**, Normalized ex vivo IFNγ ELISpot counts for vaccine neoantigens that induced a de novo response (*n* = 25 neoantigens in 8 patients): longitudinal (**f**, left); after priming (**g**). Spot counts of the non-stimulated controls were subtracted. Proportion of patients with monotope compared with polytope responses to all vaccine neoantigens (**f**, right). *n* indicates individual patients. Chemo, chemotherapy (mFOLFIRINOX). *P* values calculated using two-tailed unpaired *t*-test (**d**) or Wilcoxon matched-pairs signed-rank test (**f**). Source Data

To test this hypothesis, we conducted an investigator-initiated, phase I clinical trial of sequential adjuvant atezolizumab (Genentech), autogene cevumeran^22,23^ (an individualized mRNA neoantigen vaccine containing up to 20 major histocompatibility complex class I (MHCI) and MHC class II (MHCII) restricted neoantigens in lipoplex nanoparticles intravenously delivered; Individualized NeoAntigen-Specific Therapy (iNeST), BioNTech and Genentech) and mFOLFIRINOX in patients with surgically resectable PDAC (Fig. 1a) to : (1) amplify neoantigen-specific T cells inhibited by PD-1 signalling; and (2) prime naive T cells to vaccine neoantigens.

## Safety, feasibility and immunogenicity

**Extended Data Fig. 1:**
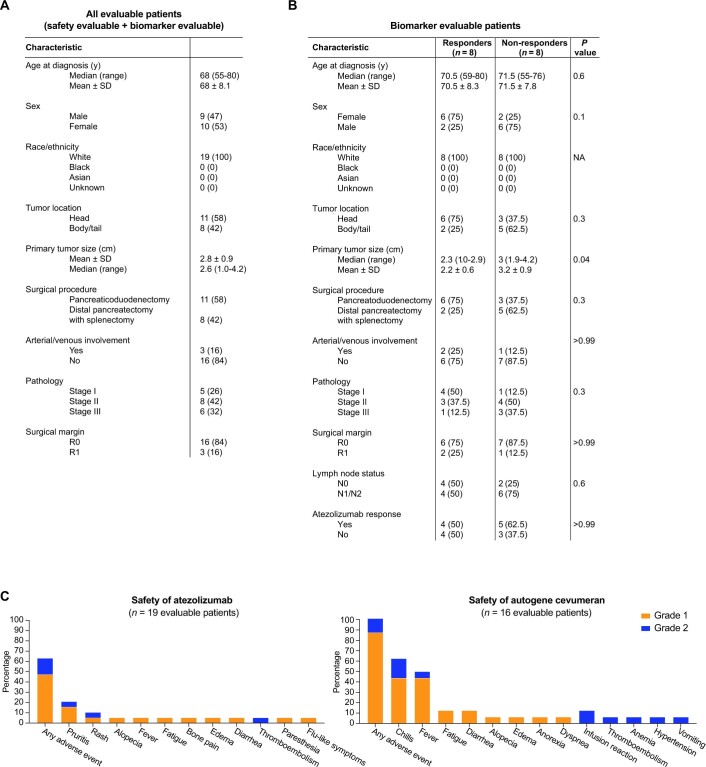
Patient demographics, clinical characteristics, and toxicity. (**A, B**) Demographics and clinical characteristics of all evaluable patients (*n* = 19) (**A**) and biomarker-evaluable patients (*n* = 16) stratified by autogene cevumeran responders and non-responders (**B**). (**C**) Frequency of grade 1 and 2 adverse events attributable to atezolizumab (**left**) and vaccine (**right**) in evaluable patients who received each drug. *n* = individual patients. Data are *n* (%) unless noted. *P* values by two-tailed Mann-Whitney test for numerical variables and Fisher’s exact test for categorical variables. Source Data

From December 2019 to August 2021, we enrolled 34 patients, of which 28 patients (Fig. 1b) underwent surgery. We then treated 19 patients with atezolizumab, of which 16 patients received subsequent autogene cevumeran. Fifteen out of these 16 patients also received subsequent mFOLFIRINOX (Fig. 1b). We analysed safety in a safety-evaluable cohort (*n* = 19 patients treated with atezolizumab, *n* = 16 treated with autogene cevumeran), and we correlated immune response to RFS in a biomarker-evaluable cohort (*n* = 16 patients treated with atezolizumab and autogene cevumeran). All 19 evaluable participants had clinical characteristics typical of patients with resectable PDAC (Extended Data Fig. 1a). All patients were treated and followed at the Memorial Sloan Kettering Cancer Center (MSK) during and beyond the enrolment period.

None of the 19 patients treated with atezolizumab in the safety-evaluable cohort had grade 3 or higher adverse events (AEs; Fig. 1c). One out of 16 (6%) patients treated with autogene cevumeran in the safety-evaluable cohort had grade 3 AEs (fever and hypertension; Fig. 1c). All 16 patients (100%) who received autogene cevumeran had grade 1–2 AEs (Extended Data Fig. 1c). We administered atezolizumab and autogene cevumeran at median times within 1 and 3 days of respective benchmarked times (median time to atezolizumab was 6.1 weeks (range of 4.3–7.9 weeks); median time to autogene cevumeran was 9.4 weeks (range of 7.4–11.0 weeks); Fig. 1d). Only 1 patient out of 19 (5%) had insufficient neoantigens that led to non-manufacture of the vaccine (Fig. 1b). Three out of 16 patients (19%) did not receive all 9 vaccine doses (Fig. 1d), which was due to progression, death or mFOLFIRINOX toxicity. Thus, autogene cevumeran can be rapidly administered even after complex oncologic surgery.

**Extended Data Fig. 2:**
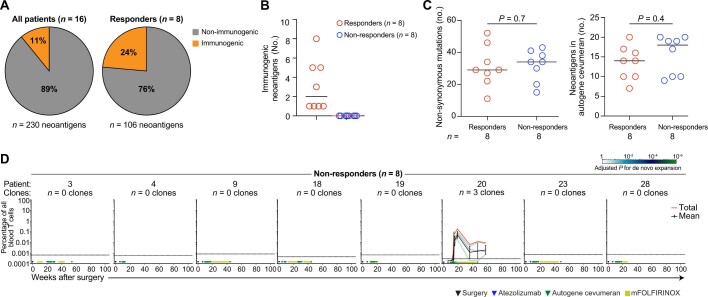
Immunogenic autogene cevumeran neoantigens. (**A**) Frequency of immunogenic autogene cevumeran (vaccine) neoantigens in all patients (**left**) and in vaccine responders (**right**). (**B**) Number of immunogenic vaccine neoantigens per patient. (**C**) Number of non-synonymous mutations (**left**) and vaccine neoantigens (**right**) in vaccine responders and non-responders. (**D**) Vaccine-expanded T cell clones by CloneTrack in non-responders. *n* = neoantigens or patients as noted. *P* values by two-tailed Mann-Whitney test (**C**) and modified two-tailed Fisher’s exact test (**D**). Source Data

Next, to measure the T cell responses induced by autogene cevumeran, we utilized a previously described ex vivo IFNγ ELISpot assay^24^ that detects high-magnitude T cell responses to vaccines without distinguishing CD8^+^ from CD4^+^ T cell responses. Eight out of 16 (50%) patients who received the vaccine generated T cell responses that were detected by ex vivo IFNγ ELISpot, and were deemed autogene cevumeran responders (Fig. 1e). By testing each specific target included in the neoantigen vaccines, we detected that 25 out of the 230 neoantigens (11%) administered across all patients who were evaluable at the single-target level induced a T cell response of sufficient high magnitude to be detectable by ex vivo IFNγ ELISpot (Fig. 1e and Extended Data Fig. 2a). Half of all the patients who received the vaccine mounted neoantigen-specific T cell responses against at least one vaccine neoantigen (median = 2, range = 1–8; Extended Data Fig. 2b). Furthermore, half of these responses were polytopic, targeting more than one vaccine neoantigen (Fig. 1e,f). No T cell responses against vaccine neoantigens were detectable before vaccination by ex vivo IFNγ ELISpot (Fig. 1f). Neoantigen-specific immune responses after vaccination were detected at levels that ranged from approximately 100 spots per million bulk peripheral blood mononuclear cells (PBMCs) to >2,000 spots per million bulk PBMCs (Fig. 1g). Inter-patient variation in the number and magnitude of all responses and intra-patient variation in the magnitude of polytopic responses were observed (Fig. 1g). Thus, autogene cevumeran induces substantial de novo T cell responses in a large proportion of patients with PDAC.

## T cell clonotypes and phenotypes

**Fig. 2:**
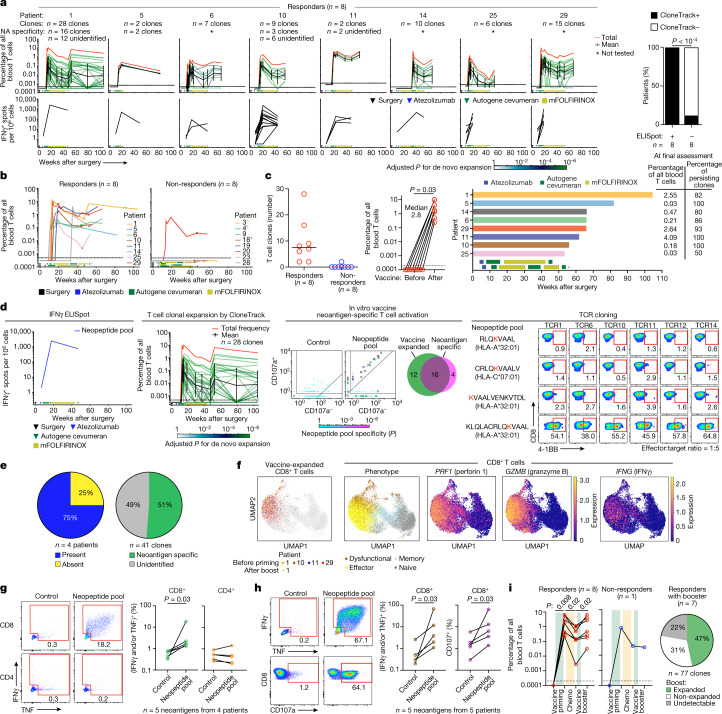
mRNA vaccines expand polyclonal, polyfunctional effector CD8^+^ T cells. **a**, Vaccine-expanded T cell clones assessed using CloneTrack (top), vaccine-induced IFNγ by ELISpot (bottom) and their correlation (right). **b**,**c**, Vaccine-expanded clones identified by CloneTrack: longitudinal aggregate percentage (**b**), number of unique clones (**c**, left), before vaccine and peak expansion aggregate percentage (**c**, middle), and final per patient assessment times (bar graph) with aggregate percentage and clonal fraction at final assessment (**c**, right). For **b**, inverted triangles indicate collection times for single-cell sequencing in **f** and circles indicate vaccine booster. **d**, Immunodominant vaccine neoantigen-specific clonal overlap with vaccine-expanded clones and specificity to immunodominant vaccine-neoantigens by TCR cloning in patient 1. **e**, Left, Percentage of patients with immunodominant vaccine neoantigen-specific clones in vaccine-expanded clones. Right, Percentage of vaccine-expanded clones specific to immunodominant vaccine neoantigens. **f**, Single-cell phenotypes of vaccine-expanded CD8^+^ T cells. Dots indicate blood CD8^+^ T cells. Coloured dots (far left) indicate vaccine-expanded clones in **a**. **g**,**h**, Percentage of IFNγ^+^, TNF^+^ and CD107a^+^CD8^+^ (**g**,**h**) and CD4^+^ T cells (**g**) in PBMCs after vaccine priming with ex vivo immunodominant long (**g**) or minimal (**h**) neopeptide rechallenge. Representative flow plots from patient 1 (**g, h**). Pregated on CD3^+^CD56^–^CD8^+^ (**g**,**h**) or CD4^+^ (**g**) cells. **i**, Left, Aggregate percentage of vaccine-expanded clones with priming, chemotherapy and booster in peripheral blood. Right, Percentage of primed clones that re-expand with booster. *n* indicates individual clones or patients. In **a** and **d**, the green lines indicate individual clone trajectories; the black line, the geometric mean clonal trajectory (error bars are the geometric s.d.); the red line, the cumulative percentage of all expanded clones. In **b,c,** asterisks indicate altered treatment schedules. In **b–d**, rectangles indicate the treatment sequence. *P* values calculated using modified two-tailed Fisher’s exact test (**a**,**d**, left), two-sided Chi square test (**a**, right), two-tailed paired *t*-test (**c**), one-tailed binomial test with Bonferroni correction (**d**, middle) or two-tailed Wilcoxon matched-pairs signed-rank test (**g**–**i**). Source Data

**Extended Data Fig. 3:**
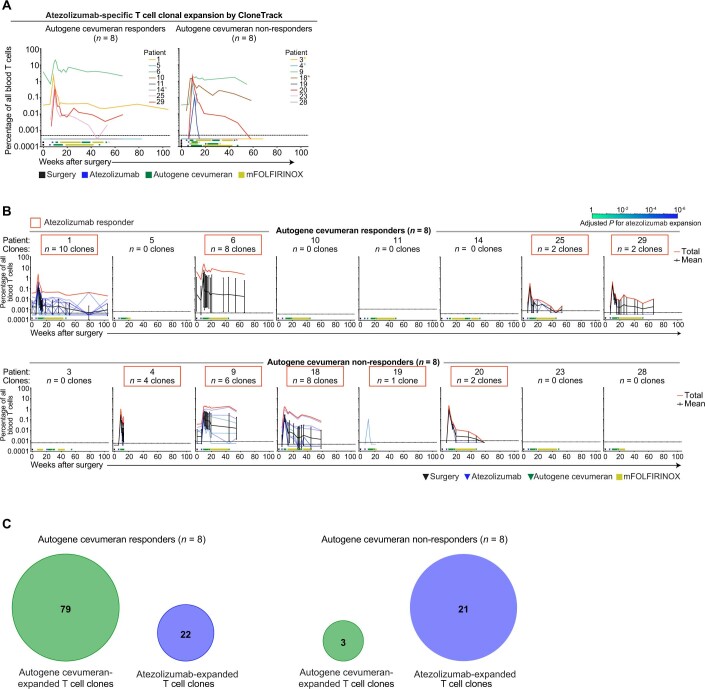
Autogene cevumeran–expanded T cell clones do not overlap with atezolizumab-expanded T cell clones. (**A**) Cumulative percentage of atezolizumab-expanded T cells by CloneTrack in autogene cevumeran (vaccine) responders and non-responders. * = altered treatment schedules for patients 3, 4, 14 and 18. (**B**) Atezolizumab-expanded T cell clones by CloneTrack in vaccine responders and non-responders. Red box = atezolizumab responder. Blue line = trajectory of an individual atezolizumab-expanded clone. Black line = geometric mean trajectory of all atezolizumab-expanded clones; error bars = geometric standard deviation. Red line = cumulative percentage of all atezolizumab-expanded clones. Black rectangle/triangle = time of surgery; blue rectangle/triangle = time of atezolizumab; green rectangle/triangle = times of autogene cevumeran (vaccine); yellow bars = mFOLFIRINOX cycles. (**C**) Venn diagrams show overlap of vaccine-expanded clones (as identified in Fig. 2a) with atezolizumab-expanded clones in vaccine responders and non-responders. *n* = number of clones or individual patients as noted. *P* values by modified two-tailed Fisher’s exact test (**B**). Source Data

**Extended Data Fig. 4:**
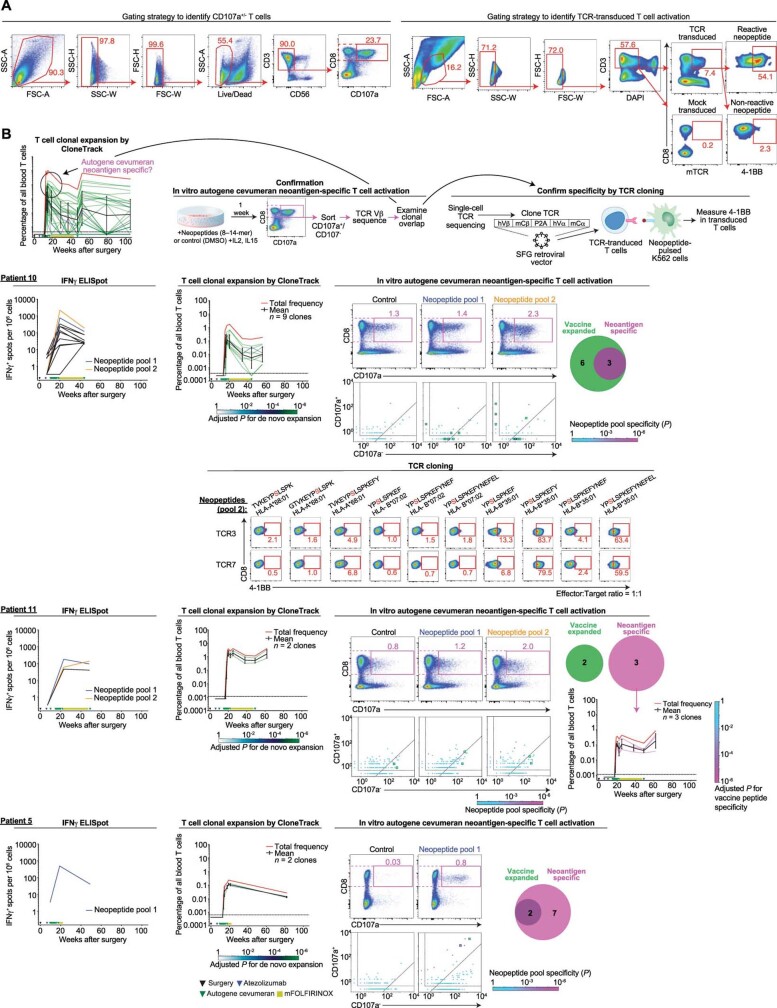
Autogene cevumeran–expanded T cell clones contain immunodominant neoantigen-specific T cells. (**A, B**) Autogene cevumeran (vaccine) induced IFNγ production by ex vivo IFNγ ELISpot (assay schematic, Fig. 1e) and clonal expansion by CloneTrack (assay schematic, **B, top**). Briefly, for ELISpot, we analyzed each patient’s PBMCs for specific T cells against all individual vaccine neoantigens post-atezolizumab/pre-vaccine, 1-3 weeks post-vaccine priming doses, and 5-6 weeks post-mFOLFIRINOX. Each patient’s PBMCs were stimulated overnight with separate pools of overlapping peptides (15 amino acids long), each pool representing one of up to 20 neoantigens in vaccines, or with anti-CD3 antibody as a positive control, followed by measurement of IFNγ production by ELISpot. PBMCs incubated with media alone were used as a negative control. To track T cell clones, we identified vaccine-expanded T cell clones with CloneTrack. To identify if expanded clones contained immunodominant neoantigen-specific clones, in 4 of 8 responders, we stimulated vaccine-expanded PBMCs collected 1-3 weeks post-vaccine priming doses in vitro with computationally predicted minimal neopeptide pools (8-14 amino acids long) from 6 neoantigens that generated the highest per-patient magnitude response by ex vivo IFNγ ELISpot. We then purified CD8^+^ T cells that either expressed or did not express the degranulation marker CD107a, identified clones with greater proportion of CD107a^+^ versus CD107a^-^ cells as in vitro neoepitope-activated clones, and examined overlap of in vitro neoepitope-activated to in vivo vaccine-expanded clones (Venn diagrams). For select patients, we further validated neoepitope-specificity by TCR cloning. (**A**) Flow cytometry gating strategies. (**B**) Ex vivo IFNγ ELISpot and T cell clonal expansion by CloneTrack in *n* = 3 of 4 patients tested (fourth patient in Fig. 2d). (**B, left**) Normalized ex vivo IFNγ ELISpot spot count per 1 x 10^6^ PBMCs for each immunogenic neoantigen in patients 10, 11 and 5. In patient 5, one neoantigen induced a high magnitude T cell response, while patients 10 and 11 had polytopic high magnitude responses against 8 and 3 vaccine neoantigens respectively. Black lines = individual neoantigens; coloured lines = neoantigen pools. (**B, middle**): T cell clonal expansion in patients 10, 11 and 5 by CloneTrack. Green line = trajectory of an individual expanded clone. Black line = geometric mean trajectory of all expanded clones; error bars = geometric standard deviation. Red line = cumulative percentage of all expanded clones. Black triangle = time of surgery; blue triangle = time of atezolizumab; green triangles = times of autogene cevumeran (vaccine); yellow bars = mFOLFIRINOX cycles. Dotted black line = detection threshold. (**B, right**): In vitro neoantigen-specific activation. Flow cytometry = CD107a expression on CD8^+^ T cells stimulated with neopeptides or control (DMSO). Dot plots = number of CD107a^+^ versus CD107a^-^ cells per T cell clone. Each circle = individual T cell clone. Green/blue squares = clones also detected by CloneTrack. Diagonal: CD107a^+^ clone frequency = CD107a^-^ clone frequency. Venn diagram = vaccine-expanded and in vitro neoantigen-specific clonal overlap. For patient 11, immunodominant neoantigen-specific clones resided in a lower magnitude vaccine-expanded clonal pool. (**B, right, bottom**) TCR cloning, patient 10: 4-1BB expression on putative neoantigen-specific TCR-transduced CD8^+^ T cells cultured with HLA-matched, neopeptide-pulsed antigen presenting cells (HLA-transduced K562 cells). *n* = number of clones. *P* values by modified two-tailed Fisher’s exact test (**B, middle**), and by one-tailed binomial test with Bonferroni correction (**B, right**). Source Data

To confirm ELISpot assay reactivity using an orthogonal technique and to probe the diversity and specificity of autogene-cevumeran-expanded T cell clones, we developed CloneTrack. CloneTrack is a new mathematical and immunological method that uses T cell receptor (TCR) Vβ sequencing of peripheral blood samples before and after treatment to identify treatment-expanded high-magnitude T cell clones (Methods). Using CloneTrack, we detected vaccine-induced clonal expansion in 8 out of 8 responders and in 1 out of 8 non-responders (Fig. 2a,b and Extended Data Fig. 2d). In responders, autogene cevumeran expanded multiple clones (median of 7.5 clones; Fig. 2c) from undetectable levels to up to 10% (median of 2.8%; Fig. 2c) of all blood T cells. Analogously, we applied CloneTrack to peripheral blood samples collected before and after atezolizumab treatment and found that autogene-cevumeran-expanded T cell clones did not overlap with atezolizumab-expanded T cell clones (Extended Data Fig. 3a–c). To assess the antigen specificity of autogene-cevumeran-expanded T cell clones, we identified T cell clones specific to ELISpot-identified immunodominant neoantigens in vitro and examined the clonal overlap to autogene-cevumeran-expanded clones in vivo in 2 patients with monotopic responses and in 2 patients with polytopic responses (Fig. 2d and Extended Data Fig. 4a,b). Three out of 4 patients (75%) and 51% (*n* = 21 out of 41 clones) of vaccine-expanded high-magnitude clones (28 out of 41 clones detected in 1 patient) contained immunodominant neoantigen-specific clones (Fig. 2e). In the fourth patient (patient 11), the immunodominant neoantigen-specific clones were in a lower magnitude vaccine-expanded clonal pool (Extended Data Fig. 4b). Thus, autogene cevumeran expands de novo polyclonal neoantigen-specific T cells in PDAC.

**Extended Data Fig. 5:**
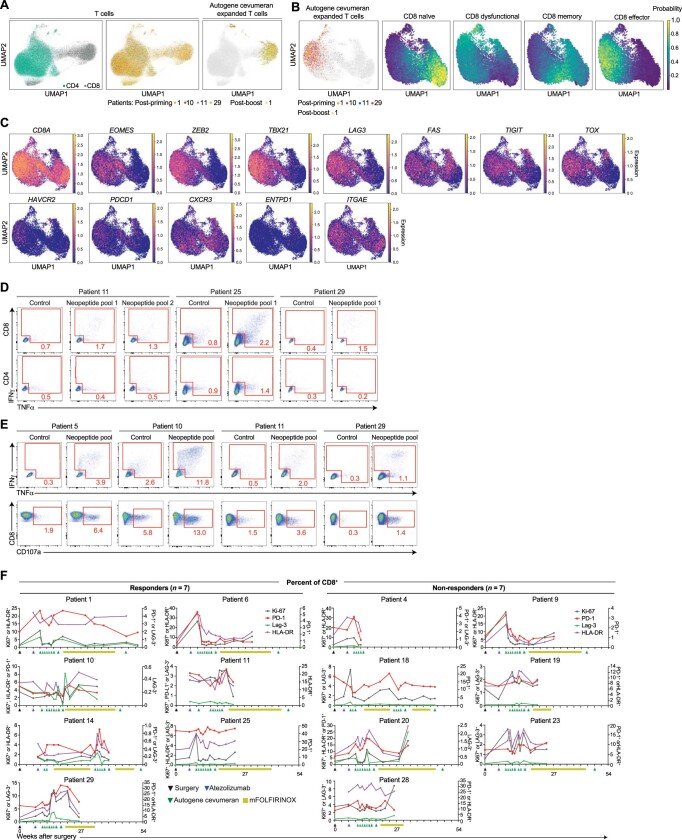
Autogene cevumeran activates neoantigen-specific polyfunctional effector CD8^+^ T cells. (**A**) Uniform manifold approximation and projection (UMAP) plots of single peripheral blood T cells by single-cell RNA/TCR sequencing in *n* = 4 patients (patients 1, 10, 11 and 29) stratified by lineage (CD8 vs. CD4, **left**), patient (**middle**), and vaccine-expanded clones (**right**; expanded clones identified in Fig. 2a). T cells purified post-vaccine priming doses at time points indicated in Fig. 2b (inverted triangles). (**B, C**) UMAP plots of single peripheral blood CD8^+^ T cells in patients 1, 10, 11 and 29 stratified by CD8^+^ T cell naïve (*SELL*, *CCR7, IL7R, BCL2, PECAM1, TCF7, BACH2, LEF1*), dysfunctional (*TIGIT, TOX, LAG3, ENTPD1, CXCL13, HAVCR2, GZMB*), memory (*EOMES, GZMK, CXCR3, TCF1, ID3, STAT4, CCR7, SELL*) and effector (*PRF1, GZMB, GNLY, IFNγ, EOMES, ZEB2, E2F7, TBX21, PDCD1, CXCR3, FAS*) transcriptional phenotypes (**B**) and select individual phenotype defining markers (**C**). (**D**) Cytokine (IFNγ, TNFα) production in CD8^+^ and CD4^+^ T cells after post-vaccine bulk PBMC ex vivo rechallenge with pools of overlapping long neopeptides. (**E**) Cytokine (IFNγ, TNFα) production and degranulation (CD107a) by CD8^+^ T cells after bulk PBMC ex vivo rechallenge with predicted minimal neopeptides. Flow cytometry gating strategies as in Extended Data Fig. 4a. (**F**) Peripheral blood CD8^+^ T cell proliferation (Ki67) and activation (PD-1, LAG-3, TIM-3, HLA-DR) pre- and serially post-atezolizumab, vaccine and mFOLFIRINOX by flow cytometry in *n* = 7 of 8 responders and *n* = 7 of 8 non-responders with available data. *n =* number of patients. Source Data

**Extended Data Fig. 6:**
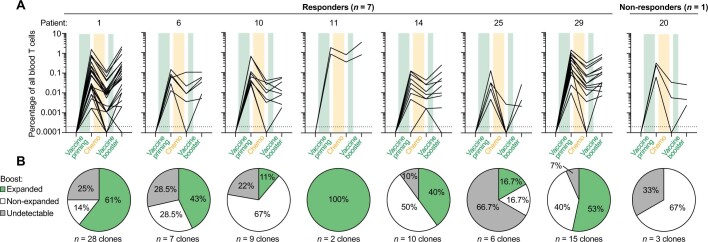
Post-mFOLFIRINOX autogene cevumeran booster re-expands primed T cell clones. (**A**) CloneTrack-identified autogene cevumeran (vaccine) expanded T cell clones with vaccine priming, mFOLFIRINOX chemotherapy (chemo) and vaccine booster. Data shown for patients that had detectable vaccine-expanded clones and received the vaccine booster. (**B**) Percentage of vaccine-expanded T cell clones that are detectable and re-expand with vaccine booster in responders. *n =* number of patients or clones. Source Data

We next studied the phenotype and function of autogene-cevumeran-expanded T cells. Using single-cell RNA sequencing, we found that autogene-cevumeran-expanded high-magnitude clones were CD8^+^ T cells that expressed lytic markers (perforin 1 and granzyme B) and cytokines (IFNγ) and resembled effector T cells induced by protective viral vaccines^25^ (Fig. 2f and Extended Data Fig. 5a–c). Consistently, peripheral blood samples collected after vaccination contained polyfunctional CD8^+^ T cells but not CD4^+^ T cells that produced cytokines (IFNγ and TNF) and degranulated on in vitro rechallenge with both long neopeptides (Fig. 2g and Extended Data Fig. 5d) and MHCI-restricted minimal epitopes (Fig. 2h and Extended Data Fig. 5e). Notably, autogene-cevumeran-expanded T cells maintained functionality despite post-vaccination mFOLFIRINOX treatment, with persistent IFNγ production (Fig. 1f), uniform re-expansion with a vaccine booster in all responders (Fig. 2i) and sustained persistence as high as 2.5% of all blood T cells up to 2 years after surgery (Fig. 2c and Supplementary Table 1). Furthermore, vaccine boosters re-expanded identical primed clones in 7 out of 7 patients who received boosters (47% of all primed clones; Fig. 2i and Extended Data Fig. 6). Although autogene cevumeran expanded multiple clones, standard flow cytometry did not reliably detect T cell expansion and activation (Extended Data Fig. 5f). Collectively, autogene cevumeran substantially expanded T cells that included vaccine neoantigen-specific, functional and durable CD8^+^ T cells.

## Vaccine response and clinical outcome

**Fig. 3:**
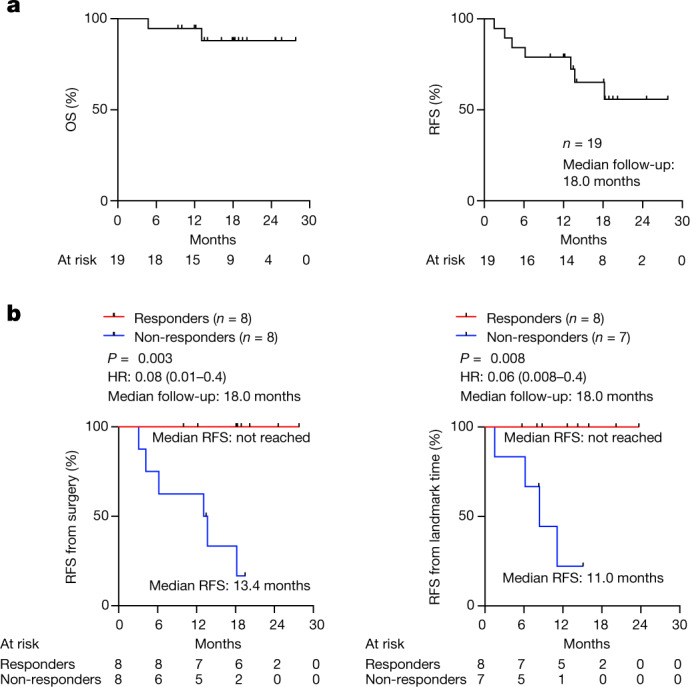
mRNA vaccine response correlates with delayed PDAC recurrence. **a**, OS and RFS in *n* = 19 patients in the safety-evaluable cohort. **b**, RFS from surgery and from landmark time (date of the last vaccine priming dose) stratified by vaccine response in patients in the biomarker-evaluable cohort. *n* indicates individual patients. HR indicates hazard ratio with 95% CI. *P* values calculated using two-tailed log-rank test.

**Extended Data Fig. 7:**
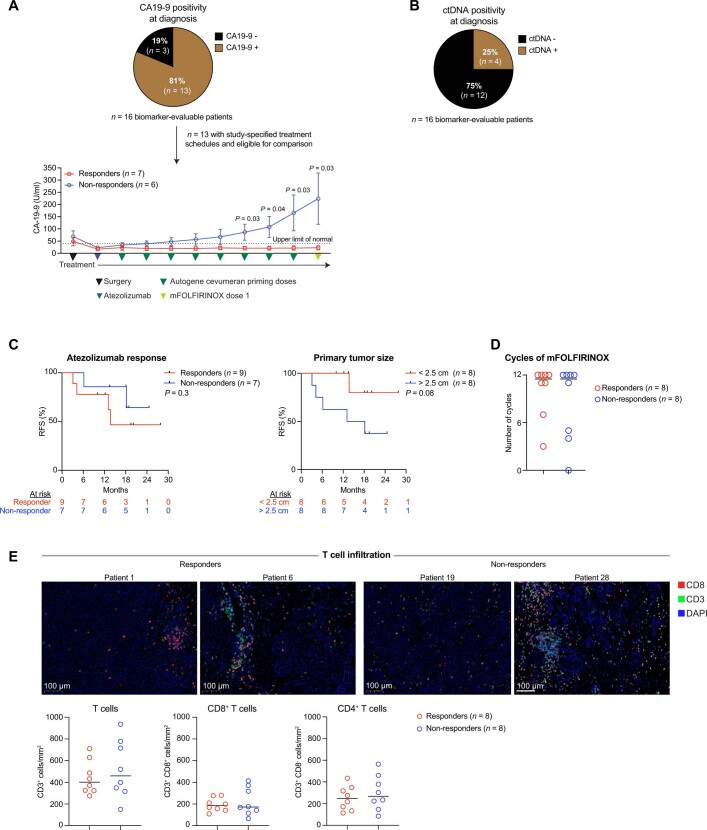
Autogene cevumeran responders evidence lower post-vaccination serum CA19-9, equivalent chemotherapy doses and comparable intratumoural T cells. (**A, B**) Fraction of biomarker-evaluable patients with detectable CA19-9 (**A**) or circulating tumour DNA (ctDNA) (**B**) in the peripheral blood at diagnosis. Longitudinal serum CA19-9 levels in autogene cevumeran (vaccine) responders and non-responders (**A, bottom**). Circle = mean, error bars = standard error of the mean (**C**) Recurrence-free survival (RFS) stratified by atezolizumab response (**left**) and median primary tumour size (**right**). (**D**) Number of cycles of mFOLFIRINOX (chemotherapy) in vaccine responders and non-responders. (**E**) Intratumoural T cell infiltration in autogene cevumeran responders and non-responders. *n* = individual patients. *P* values by two-tailed Mann Whitney test (**A**), and log-rank test (**C**). Source Data

**Extended Data Fig. 8:**
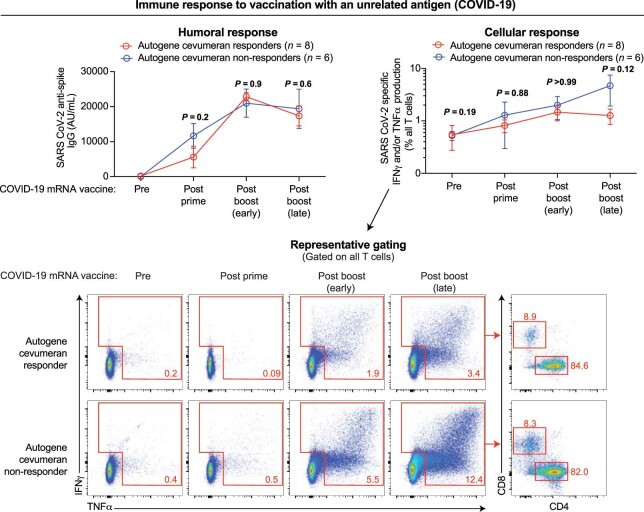
Autogene cevumeran responders and non-responders have equivalent humoral and cellular responses to an unrelated mRNA vaccine. (**Top left**) Anti-SARS-CoV-2 spike IgG in sera of vaccine responders and non-responders before (pre), after priming (post-prime) and after booster (post-boost early and late) doses of COVID-19 mRNA vaccine (Pfizer-BioNTech BNT162b2 or Moderna Spikevax). (**Top right**) Serial PBMCs pre- and post-COVID-19 mRNA vaccination were stimulated with SARS CoV-2 peptide pools. SARS CoV-2-specific IFNγ and/or TNFα production by all T cells was measured by flow cytometry. Composite data (**top right**) with representative gating (**bottom**) are shown. Flow cytometry pre-gated on CD3^+^ CD56^−^ cells. Circle = mean, error bars = standard error of the mean; *n* = individual patients; data shown for all patients with available samples. *P* values by two-tailed Wilcoxon matched-pairs signed rank test. Source Data

**Extended Data Fig. 9:**
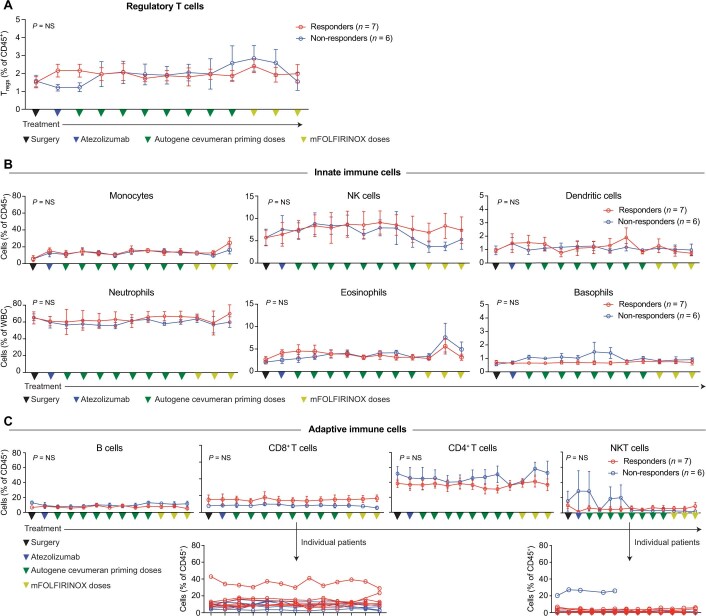
Autogene cevumeran responders and non-responders have equivalent frequencies of circulating immune cells. (**A-C**) Longitudinal frequencies of regulatory T cells (**A**), innate (**B**) and adaptive (**C**) immune cells in the peripheral blood of autogene cevumeran responders and non-responders during vaccination. Analyses in *n* = 13 patients with identical study-specified treatment schedules and thus eligible for direct comparison. Circle = mean, error bars = standard error of the mean; *n* = individual patients. *P* values by two-tailed Mann Whitney test. Source Data

At a median follow-up of 18 months that extended beyond the prespecified secondary end point, the median OS and RFS of the patients in the safety-evaluable cohort were not reached (Fig. 3a). For patients in the biomarker-evaluable cohort, the 8 autogene cevumeran responders had a median RFS that was not reached compared with the 8 non-responders who had a median RFS of 13.4 months (*P* = 0.003, hazard ratio (HR) = 0.08 (95% confidence interval (CI) 0.01–0.4); Fig. 3b). To exclude a time-to-response bias^26^, we performed a landmark analysis to correlate RFS to response in patients who were recurrence-free when completing all 8 autogene cevumeran priming doses (landmark RFS). The median landmark RFS was similarly not reached in responders compared with 11.0 months in non-responders (*P* = 0.008, HR = 0.06 (95% CI 0.008–0.40); Fig. 3b). Consistently, compared with non-responders, responders had persistently lower serum CA19-9 levels (Extended Data Fig. 7a), the most widely used clinical PDAC biomarker^27^. Only 25% of patients in the biomarker-evaluable cohort had detectable circulating tumour DNA at diagnosis (Extended Data Fig. 7b), as previously reported in patients with resectable PDAC tumours^28,29^, and thus was not a reliable biomarker of recurrence. To identify if responders were merely enriched in patients with better prognosis, we found response to atezolizumab, lymph node positivity, margin positivity, primary tumour size, the number of chemotherapy doses and density of intratumoural CD8^+^ T cells did not correlate with vaccine response (Extended Data Figs. 1b and 7c–e). Responders and non-responders also had comparable immunological fitness, as they mounted equivalent humoral and cellular responses to an unrelated mRNA vaccine (SARS-CoV-2) that was concurrently administered with autogene cevumeran (Extended Data Fig. 8). Responders and non-responders also had equivalent peripheral frequencies of all major innate and adaptive immune cells (Extended Data Fig. 9a–c), and similar somatic and germline genetic characteristics (Supplementary Tables 2–4). In summary, the autogene-cevumeran-expanded T cell response correlates with delayed PDAC recurrence that is not confounded by detectable differences in patient selection, intratumoural T cell frequency or peripheral T cell frequency or fitness.

**Extended Data Fig. 10:**
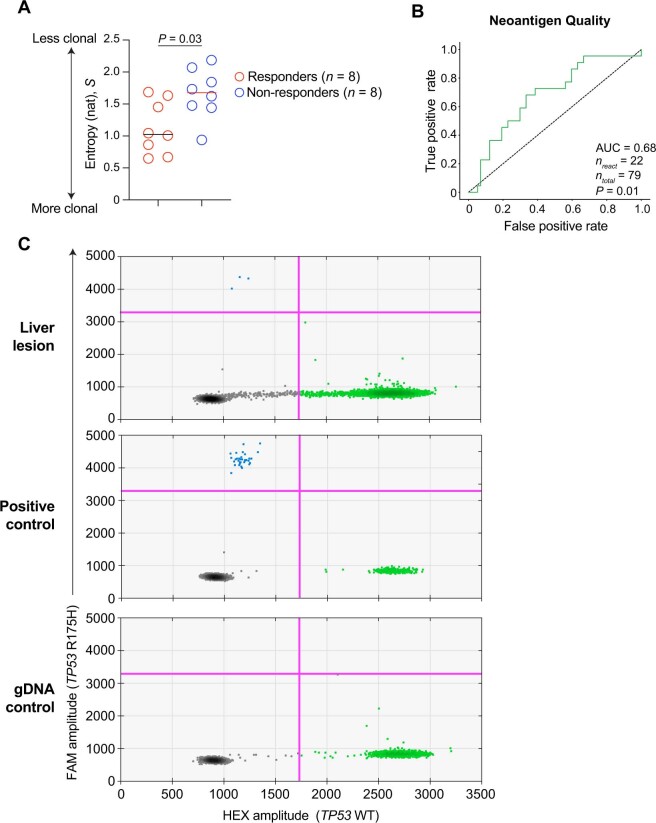
Tumour clonality and neoantigen quality in biomarker-evaluable patients, and mutated *TP53* in a disappearing liver lesion. (**A**) Shannon entropy (*S*) of tumour clones in autogene cevumeran (vaccine) responders and non-responders. (**B**) Receiver operating curve indicating the ability of neoantigen quality as a continuous variable to classify vaccine neoantigens as an inducer or non-inducer of an IFNγ ELISpot response. Dotted line = all neoantigens included in individualized mRNA vaccines. (**C**) Digital droplet PCR showing number of wild-type (WT) *TP53*, or R175H mutated *TP53* droplets in liver lesion from patient 29 (Fig. 4), with positive and negative (gDNA) controls. *n* = individual patients (**A**) or neoantigens (**B**). *P* values by two-tailed Mann-Whitney test. Source Data

As autogene cevumeran induced high-magnitude T cell responses specific to 25 out of 106 vaccine-encoded neoantigens (24%) in responders (Extended Data Fig. 2a), we searched for correlates of vaccine response. Our previous findings^2,3,30^ showed that CD8^+^ T-cell-enriched PDAC tumours are also enriched in immunogenic ‘high-quality’ neoantigens distributed in greater proportions across tumour clones. Therefore, we examined whether tumour clonality and neoantigen quality correlate with vaccine-induced T cell responses. Consistently, responders to autogene cevumeran had more clonal tumours than non-responders (Extended Data Fig. 10a). Next, we examined whether immunogenic neoantigens in responders contained high-quality features. We adapted our previously described model^2,3^ (Methods) that identifies spontaneously targeted neoantigens in tumours to correlate immunogenicity to the quality of vaccinated neoantigens. In responders, neoantigen quality as a continuous variable correlated with vaccine neoantigens that induced IFNγ ELISpot responses (Extended Data Fig. 10b). Notably, non-responders had similar numbers of non-synonymous mutations and vaccine neoantigens as responders (Extended Data Fig. 2c).

## Vaccine clones and a micrometastasis

**Fig. 4:**
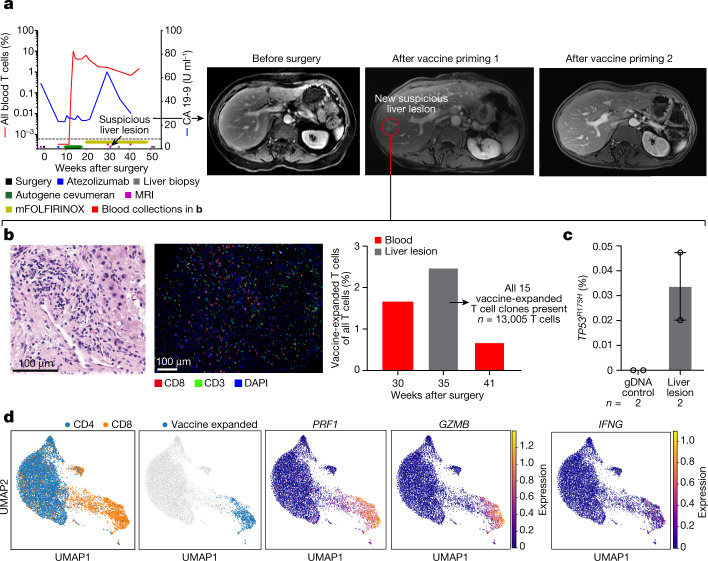
Vaccine-expanded T cells can infiltrate a micrometastasis. Clinical and immunological snapshot of a disappearing intrahepatic lymphoid aggregate after vaccination in a patient who responded to the vaccine. **a**, Serial percentage of vaccine-expanded T cells in blood analysed using CloneTrack and serum CA19-9 (left), and abdominal MRI (right) before and after vaccination. **b**, Haematoxylin and eosin staining (left), multiplexed immunofluorescence (middle) and percentage of vaccine-expanded T cells measured using CloneTrack (right, grey bar) in a new liver lesion that developed after vaccination detected by MRI as in **a**. All 15 vaccine-expanded T cell clones (**a**, red line) were present in liver lesion (right, grey bar). **c**, Percentage of mutant *TP53*^R175H^ reads by digital droplet PCR in the liver lesion. The bar indicates the median, the error bars are the s.e.m. **d**, Uniform manifold approximation and projection (UMAP) plots of single-cell phenotypes of all blood T cells (left) and vaccine-expanded clones (middle), with effector markers (right). *n* indicates the number of T cells detected in liver lesion (**b**) or technical replicates (**c**). Data represent analyses of a single patient. Source Data

Patient 29 responded to autogene cevumeran with the second-highest maximal percentage of expanded blood T cells (Fig. 2b) that included vaccine neoantigen-specific polyfunctional CD8^+^ T cells (Extended Data Fig. 5d,e). Patient 29 developed increased serum CA19-9 levels with a new 7-mm liver lesion suggestive of a metastasis after vaccine priming (Fig. 4a). A biopsy sample did not reveal malignant cells but a dense lymphoid infiltrate (Fig. 4b, left) that included all 15 autogene-cevumeran-expanded (Fig. 4b, middle) CD8^+^ T cell clones with phenotypic evidence of lytic and effector potential (Fig. 4d). Digital droplet PCR revealed that this lymphoid infiltrate contained rare cells harbouring the *TP53*^R175H^ mutation, identical to the R175H driver mutation in the primary tumour of this patient (Fig. 4c and Extended Data Fig. 10c). This liver lesion disappeared on subsequent imaging (Fig. 4a), which suggests that autogene-cevumeran-expanded T cells may possess the capacity to eradicate micrometastases.

## Discussion

We demonstrated that adjuvant autogene cevumeran, an individualized neoantigen vaccine based on uridine mRNA–lipoplex nanoparticles, in combination with atezolizumab and mFOLFIRINOX, is safe, feasible and generates substantial neoantigen-specific T cells in 50% of unselected patients with resectable PDAC. Vaccine-expanded T cells were durable, persisting up to 2 years despite post-vaccination mFOLFIRINOX treatment. High-magnitude vaccine-induced T cell responses, the focus of our immune response analysis that included a new method to track vaccine-expanded clones, correlated with delayed PDAC recurrence. Despite the limited sample size, these early results warrant larger studies of individualized mRNA neoantigen vaccines in PDAC.

As multiple immunotherapies^31^ have emerged for immune-inflamed tumours, there remains a need for new immunotherapies for the majority of patients with non-inflamed tumours that are largely insensitive to current immunotherapies. Indeed, the prevailing thought has been that the low passenger mutation rate of such tumours renders them with insufficient neoantigens for vaccines. Here, we provided evidence that despite the low mutation rate of PDAC, a mRNA vaccine can induce T cell activity against neoantigens in this cancer, a non-inflamed tumour with predominantly immune-excluded or desert phenotypes. Whether mRNA neoantigen vaccines can similarly activate T cells in other non-inflamed cancers should be more broadly tested.

We did not find evidence that the correlation of vaccine response to delayed recurrence is confounded by known prognostic variables, such as lymph node or margin-positive disease. Non-responders on average had slightly larger primary tumours than responders; however, larger primary tumour size did not correlate with shorter RFS. As the uridine mRNA–lipoplex vaccine technology is based on potent antigen delivery into lymphoid compartments and stimulates weak T cell responses in splenectomized mice^20^, it is notable that non-responders were also marginally enriched in patients with splenectomies (Extended Data Fig. 1b). Furthermore, that vaccines induced high-magnitude T cell responses in 50% of patients may highlight the need for biomarkers to select optimal patients and tumours for this treatment. Of note, although autogene cevumeran is designed to activate both neoantigen-specific CD4^+^ and CD8^+^ T cells and we find it activates high magnitude CD8^+^ T cells in PDAC, the primary and confirmatory immune response assays in this study do not distinguish CD8^+^ from CD4^+^ T cell responses. In fact, as these assays bias towards high-magnitude T cell responses, assays that detect lower magnitude responses may include both CD4^+^ T cell responses and pre-existing responses. In other tumours, we observed that a substantial proportion of vaccine neoantigens induce de novo responses that are below the ex vivo detectable threshold (manuscript in preparation), a level of response not assessed in this trial. Nevertheless, our results in PDAC imply that a high-magnitude T cell response may contribute to a favourable clinical outcome. Thus, strategies to ensure high-magnitude responses are being pursued, including further optimization of mRNA vaccine potency and extension of the neoantigen discovery space to include genetic aberrations other than single nucleotide variations (SNVs) and insertions and deletions (indels) (for example, fusions)^32^. Notwithstanding, as vaccines expand polyclonal T cells, whether vaccine-induced clonal diversity contributes to durable control^33,34^ is another key query for future work.

Our study was not powered to detect differences in biomarkers of vaccine response. Despite this limitation, we observed that tumours in responders were more clonal—possibly representing tumours in immune-edited evolution as seen in immunogenic PDACs in long-term survivors^3^. Thus, we speculate that a more clonal primary tumour may reflect the ability of the immune system to recognize a tumour and therefore respond to a vaccine. Moreover, the observation that neoantigen quality^2,3,30^ correlates with immunogenic vaccine neoantigens provides further support for the concept that select neoantigens may possess higher immunogenic qualities that are possibly desirable for vaccines. However, in this trial, as responders and non-responders had comparable numbers of vaccine neoantigens drawn from a similar number of tumour mutations, we consider that an absence of a response in non-responders is unlikely due to a failure to include immunogenic neoantigens. Overall, these observations remain preliminary but support future investigation of whether tumour clonality and neoantigen quality could serve as biomarkers of vaccine response.

We tested individualized mRNA cancer vaccines in the adjuvant setting motivated by observations that vaccines against pathogens have historically been most effective in preventive and not therapeutic settings, which likely reflects that vaccine efficacy requires an optimally functioning host immune system. In patients with advanced cancer, global impairments in host immunity and knowledge gaps on neoantigen heterogeneity between tumours may hamper neoantigen vaccination. Thus, we propose that vaccines should be tested in patients with minimal residual disease, as is currently ongoing in trials in high-risk colorectal cancer (ClinicalTrials.gov identifier NCT04486378) and in triple-negative breast cancer (ClinicalTrials.gov identifier NCT02316457). Notably, our study demonstrated that mRNA neoantigen vaccines can be individualized in 9 weeks and fully integrated into a standard clinical workflow even after complex oncologic surgery. Given this trial was the index experience with individualized mRNA vaccination for PDAC, mFOLFIRINOX was administered >12 weeks^35–37^ after surgery. Furthermore, given its limited sample size, this trial enrolled only white individuals. Future studies must test individualized mRNA neoantigen vaccines in a diverse population of patients with PDAC, coupled with a faster time to adjuvant mFOLFIRINOX. Experience with individualized cancer vaccines^16^ that predated and accelerated mRNA-based SARS-CoV-2 pandemic vaccines^38^ can now further hasten individualized cancer vaccine manufacture times^22,23^ and enable more rapid adjuvant custom vaccination and chemotherapy.

Overall, we reported preliminary evidence that adjuvant autogene cevumeran, an individualized mRNA neoantigen vaccine, in combination with atezolizumab and mFOLFIRINOX induces substantial T cell activity in patients with surgically resected PDAC that correlates with delayed recurrence. A follow-up global randomized trial (IMCODE 003, BNT122) is imminent.

## Methods

### Trial design, treatments, oversight and conduct

We administered atezolizumab, autogene cevumeran and mFOLFIRINOX sequentially to measure how each immunotherapy modulated neoantigen-specific T cells. To establish clinical feasibility, we set the following benchmarked times to treatment after surgery (Fig. 1a): (1) one 1,200 mg intravenous dose of atezolizumab on week 6; (2) nine 25 µg intravenous doses of autogene cevumeran given as seven weekly priming doses beginning on week 9, an eighth dose at week 17 and a ninth booster dose at week 46; (3) 12 cycles of mFOLFIRINOX beginning on week 21. As the half-life of atezolizumab is 27 days, with receptor occupancy persisting for several months^39,40^, we hypothesized that this dosing scheme would allow sufficient PD-L1 receptor occupancy to support proficient T cell activation by autogene cevumeran. Additional details are in provided in the protocol in Supplementary Data 1. As we treated the first patient before the COVID-19 pandemic, patients received SARS-CoV-2 vaccines as they became available either before, interspersed during or following completion of individual experimental treatments.

We conducted the study in accordance with the Declaration of Helsinki and good clinical practice guidelines. The study was approved by the institutional review board at MSK, the US Food Drug Administration and was registered on ClinicalTrials.gov (NCT04161755). All participants provided written informed consent.

### Immune response assays

We investigated peripheral blood samples using two independent assays. Both, per design, detect high-magnitude T cell responses to vaccines without distinguishing CD8^+^ from CD4^+^ T cell responses. IFNγ ELISpot was performed ex vivo (that is, without previous expansion in culture to capture T cells below the assay threshold) to map the induction of neoantigen-specific T cell responses for each vaccine target used across all patients. Patients were classified as responders to autogene cevumeran if the IFNγ ELISpot assay detected T cell reactivity against at least one vaccine neoantigen.

To confirm IFNγ ELISpot results using an orthogonal technique, we used TCR Vβ sequencing-based CloneTrack to detect greater than twofold in vivo expansion of T cell clones to vaccines in a non-antigen-specific manner. Clones that expanded at different treatment times (before and after atezolizumab; before and after autogene cevumeran) further distinguished atezolizumab from autogene-cevumeran-expanded T cell clones. For 6 ELISpot-identified immunodominant neoantigens in 4 out of 8 (50%) autogene cevumeran responders, we used TCR Vβ sequencing of in vitro neopeptide-stimulated T cells to validate the specificity of in vivo vaccine-expanded T cell clones.

### Patients

We enrolled patients with an Eastern Cooperative Oncology Group performance status of 0–1 with single, radiographically suspicious, surgically resectable PDACs, no distant metastases and ≥5 neoantigens. We excluded patients with metastatic, borderline or locally unresectable PDACs, and patients who received neoadjuvant therapy.

After surgery, we included patients with pathologically confirmed PDAC with R0/R1 margins. Additional eligibility criteria and ethical study conduct information are in the protocol (Supplementary Data 1). We aimed to accrue 20 evaluable patients.

### Surgery

Patients underwent open pancreaticoduodenectomy or either open or laparoscopic distal pancreatectomy and splenectomy at MSK. We then transported tumour blocks with the most (minimum ≥10%) histological tumour content with matched blood to BioNTech.

### End points

The primary end point was safety (Extended Data Table 1). Secondary end points were 18-month RFS and 18-month OS. We defined recurrence as new lesions on the basis of response evaluation criteria in solid tumours (v.1.1), and RFS from either the date of surgery (RFS) or from the date of the last autogene cevumeran priming dose (landmark RFS) to the date of recurrence or death, whichever occurred first. We censored patients without events at the last known date they were recurrence-free. We defined OS from the date of surgery to the date of death. As exploratory end points, we measured immune response and feasibility as actual compared with benchmarked treatment times. Data cut-off was 1 April 2022, extending the median follow-up beyond the prespecified 18-month RFS secondary end point.

### Mutation identification and neoantigen selection

DNA was extracted from PBMCs. DNA and RNA were extracted from tumours. Expressed non-synonymous mutations and HLA type were identified by whole-exome sequencing of patient-specific tumour–normal pairs and tumour RNA sequencing. Neoantigens were bioinformatically predicted and ranked by immunogenicity as previously described^16^. mRNA vaccine neoantigen characteristics are detailed in Supplementary Table 5.

### Somatic and germline mutation testing

For the detection of somatic tumour mutations in key cancer genes, we used MSK-IMPACT, a previously published targeted tumour-sequencing test that covers 468 cancer genes^41^.

For the detection of germline mutations, we utilized the MSK-IMPACT panel to detect pathogenic germline variants. In brief, gDNA was enriched for targeted regions using a hybridization-based protocol and sequenced on an Illumina HiSeq instrument. Sequence reads were aligned to the GRCh37/hg19 reference human genome and variants (SNVs, small indels, and copy number variants encompassing one or more exons) were called using publicly available and in-house developed bioinformatics tools. Variants were classified according to the American College of Medical Genetics and Genomics guidelines^42^. Only variants classified as pathogenic or likely pathogenic are reported. The list of mutations analysed in the MSK-IMPACT germline panel are provided in Supplementary Table 4.

### Production benchmarks

We set the following a priori benchmarks from surgery to vaccine manufacture: (1) transport specimen from operating room to pathology in ≤5 min; (2) fix specimen in formalin and embed in paraffin in ≤15 min; (3) select blocks for vaccine production in ≤2 days; (4) ship to BioNTech in ≤72 h; (5) produce vaccines in ≤6 weeks; (6) administer first dose of the vaccine in ≤9 weeks.

### Autogene cevumeran manufacturing

For every patient, individualized mRNA neoantigen vaccines were manufactured under good manufacturing practice conditions containing two uridine-based mRNA strands with noncoding sequences optimized for superior translational performance^43,44^. Each strand encoded up to 10 MHCI and MHCII neoepitopes, formulated in approximately 400 nm diameter lipoplex nanoparticles^20^ comprising the synthetic cationic lipid (*R*)-*N*,*N*,*N*-trimethyl-2,3-dioleyloxy-1-propanaminium chloride (DOTMA) and the phospholipid 1,2-dioleoyl-*sn*-glycero-3-phosphatidylethanolamine (DOPE) to enable intravenous delivery.

### Cell culture

We purified patient PBMCs from blood samples by density centrifugation over Ficoll-Paque Plus (GE Healthcare). We purified healthy donor PBMCs from buffy coats (New York Blood Center) and isolated T cells using a Pan-T cell isolation kit (Miltenyi Biotech). We activated T cells with CD3/CD28 beads (Thermo Fisher) with IL-7 (3,000 IU ml^–1^) and IL-15 (100 IU ml^–1^) (Miltenyi Biotec), and transduced T cells on day 2 after activation. Virus-producing cell lines (H29 and RD114-envelope producers) were as previously described^45,46^. We cultured T cells and K562 cells in RPMI medium supplemented with 10% FBS (Nucleus Biologics), 100 U ml^–1^ penicillin–streptomycin (Thermo Fisher Gibco) and 2 mM l-glutamine (Thermo Fisher Gibco). We cultured patient PBMCs with RPMI medium supplemented with 10% FBS, 1 mM sodium pyruvate, 2 mM l-glutamine, non-essential amino acids and 2-mercaptoethanol (MSK medium preparation core facility). We cultured H29, RD114-envelope producers and Phoenix-AMPHO in DMEM medium supplemented with 10% FBS (Nucleus Biologics), 100 U ml^–1^ penicillin–streptomycin (Thermo Fisher Gibco) and 2 mM l-glutamine (Thermo Fisher Gibco).

### Immune response

#### Ex vivo IFNγ ELISpot

Multiscreen filter plates (Merck Millipore), precoated with antibodies specific for IFNγ (Mabtech), were washed with PBS and blocked with X-VIVO 15 (Lonza) containing 2% human serum albumin (CSL-Behring) for 1–5 h. Next, 3 × 10^5^ effector cells per well were stimulated for 16–20 h with pools of 15-amino-acid-long peptides (JPT Peptide Technologies) overlapping by 11 amino acids covering the length of each target. Cryopreserved PBMCs were subjected to ELISpot after a resting period of 2–5 h at 37 °C. All tests were performed in duplicate and included anti-CD3 (Mabtech) as a positive control. Bound IFNγ was visualized using a secondary antibody directly conjugated with alkaline phosphatase (ELISpotPro kit, Mabtech). Next, plates were incubated with BCIP/NBT (5-bromo-4-chloro-3′-indolyl phosphate and nitro blue tetrazolium) substrate (ELISpotPro kit, Mabtech). Plates were scanned using an AID Classic Robot ELISPOT reader and analysed using AID ELISPOT 7.0 software (AID Autoimmun Diagnostika). A sample was deemed positive if the IFNγ ELISpot count exceeded a minimum threshold of 7 spots per 300,000 PBMCs. A post-vaccination PBMC sample was deemed positive on the basis of a significant increase in ELISpot count compared with a negative control (medium alone, as no IFNγ ELISpot responses were detected in pre-vaccination samples). To account for varying sample quality reflected in the number of spots in response to anti-CD3 antibody stimulation, we applied a normalization method that enabled direct comparison of spot counts and strength of response between individuals, as described previously^24,47,48^. Statistical significance was determined based on two statistical tests (distribution-free resampling)^47,48^.

#### TCR Vβ clone tracking (CloneTrack)

For TCR Vβ sequencing, we prepared gDNA from bulk PBMCs or purified T cells using a Qiagen DNA extraction kit according to the manufacturer’s instructions. We quantified samples using Dropsense 96 and diluted to standard concentrations for library preparation. We generated sample data using an immunoSEQ Assay (Adaptive Biotechnologies). In brief, the somatically rearranged TCRB CDR3 was amplified^49,50^ from gDNA using a two-step, amplification bias-controlled multiplex PCR approach. The first PCR consists of forward and reverse amplification primers specific for every known V and J gene segment, and amplifies the hypervariable CDR3 of the immune receptor locus. The second PCR adds a proprietary barcode sequence and Illumina adapter sequences^51^. In addition, reference gene primers were included in the PCR reaction to quantify total nucleated cells that can be sequenced and to accurately measure the fraction of T cells in each sample. CDR3 and reference gene libraries were sequenced on an Illumina instrument according to the manufacturer’s instructions. Raw sequence reads were demultiplexed according to Adaptive’s proprietary barcode sequences. Demultiplexed reads were further processed to remove adapter and primer sequences and to identify and remove primer dimer, germline and other contaminant sequences. The filtered data were clustered using both the relative frequency ratio between similar clones and a modified nearest-neighbour algorithm to merge closely related sequences to correct for technical errors introduced through PCR and sequencing. The resulting sequences were sufficient to annotate the V, D and J genes and the N1 and N2 regions constituting each unique CDR3 and the translation of the encoded CDR3 amino acid sequence. Gene definitions were based on annotation in accordance with the IMGT database (https://www.imgt.org). The set of observed biological TCRB CDR3 sequences were normalized to correct for residual multiplex PCR amplification bias and quantified against a set of synthetic TCRB CDR3 sequence analogues^50^.

#### T cell clone definition

We identified and tracked T cell clones by their β chain sequence (TRB), defined as the nucleotide CDR3 sequence (including the conserved C and F residues) and a deterministic V and J gene alignment. For T cells identified by single-cell sequencing, we similarly defined clones by the TRB sequence to map clones to paired TCR Vβ sequencing. Owing to the higher entropy of the nucleotide CDR3 sequence generation probability distribution^52^, we used nucleotide instead of amino acid CDR3 sequences to minimize the chance of conflating two different T cell clones (different original VDJ recombination events). This becomes crucial to differentiate clones that may have different α chain (TRA) sequences, which are unobserved in the bulk TCR Vβ sequencing step. We used the provided deterministic V and J alignments from Adaptive Biotechnologies (for bulk TCR Vβ sequencing) and 10x (for single-cell sequencing).

#### T cell clone frequency estimation

For a given sample of bulk TRB sequences, we estimated the total number of effective cells sequenced, *N*, as the summation of all productive (in-frame, no stop codons) reads. We excluded non-productive reads, as they must necessarily be recombined CDR3s from silenced alleles (we did not model the fraction of productive reads from silenced alleles and assumed them to be a small correction). We estimated a T cell clone $x$’s cell count ${n}_{x}$ in a sample as the number of reads corresponding to the clone as defined above (V and J gene and nucleotide CDR3 sequence). We therefore estimated the frequency of clone $x$ as ${f}_{x}=\frac{{n}_{x}}{N}$. For the purpose of visualization, if we did not observe a clone in a sample, we used a pseudo-frequency of $\frac{1}{3N}$ (if plotting a trajectory with multiple samples, we used the largest *N* over the samples) and indicated this observation threshold as dotted black lines. We computed the aggregate frequency of several clones $x\in X$ in a similar fashion using an aggregate count ${n}_{X}={\sum }_{x\in X}{n}_{x}$ and used the same convention for a pseudo-frequency if ${n}_{X}=0$.

#### T cell clone significance determination

We took a statistically conservative approach to minimize the false-positive identification of expanded T cell clones. To this end, to calculate treatment-expanded T cell clones, we used a significance threshold of *P*_adjusted_ < 0.001, where the *P* value is adjusted using Bonferroni correction (*P*_adjusted _ = *P* × no. of T cell clones) to account for the large number of T cell clones that were screened.

#### T cell expansion criteria

To identify treatment-expanded T cell clones, we used an adapted Fisher’s exact test and computed *P* values for expanded T cell clones using a two-tailed adapted exact Fisher’s test for a twofold increase in a T cell clone between any two samples.

We implemented this by rescaling the repertoire size of the initial sample by half, to effectively reduce the sample size and the number of cells not belonging to the clone in question. We computed this as a Fisher’s exact test (implemented from scipy.stats.fisher_exact) using the categorical table supplied in Extended Data Table 2.

Clones that had a fold change <2 (that is, $\frac{{m}_{x}}{M} < 2\times \frac{{n}_{x}}{N}$) were assigned a *P* value of 1. These *P* values were then adjusted using Bonferroni correction: ${P}_{{\rm{adjusted}}}=P\times | N\cup M|$, where $|N\cup M|$ designates the number of unique clones in the union of the two samples.

We applied this T cell clone expansion *P* value in the following two contexts to determine whether atezolizumab or autogene cevumeran expanded T cell clones.

Atezolizumab response: to determine whether atezolizumab expanded T cell clones (Extended Data Fig. 3), we compared the number of cells of a particular T cell clone in a blood sample taken on the day of but before atezolizumab administration to the number of cells of that T cell clone in a blood sample taken on the day of but before the first dose of autogene cevumeran. We then considered that a patient had a response to atezolizumab if any T cell clone was found to be significantly expanded (*P*_adjusted_ < 0.001) according to the above outlined expansion criterion.

Autogene cevumeran response with priming doses: to determine whether autogene cevumeran expanded T cell clones, we imposed two criteria: (1) a quality control requirement that a T cell clone must have a minimum of three reads in at least two samples, and (2) the T cell clone must not be observed before vaccination (0 cells in all samples taken until the day of but before the first dose of autogene cevumeran).

Then, for clones that passed these criteria (as defined above), we compared the number of cells of a particular T cell clone in a blood sample taken after atezolizumab and before vaccination to the number of cells of that T cell clone in any blood sample taken until the day of but before the first dose of mFOLFIRINOX. We further assigned an expansion *P* value as defined as the minimum adjusted expansion *P* value for all samples, further adjusted by Bonferroni correction for the number of samples the expansion *P* values were computed for.

We then considered that a patient had a response to autogene cevumeran if any T cell clone significantly expanded (*P*_adjusted_ < 0.001) according to the above outlined expansion criteria.

For 50% of the responders (*n* = 4 patients), we further examined whether the autogene-cevumeran-expanded T cell clones included neoantigen-specific in vitro clones (see the section ‘In vitro stimulation and T cell clone specificity to peptides’ below).

Autogene cevumeran response with a booster dose: to determine whether an autogene-cevumeran-expanded clone further expanded after the booster dose, we used a standard Fisher’s exact test, with no additional fold change criteria, to compare the clone sizes in samples taken immediately before booster administration to samples from the first follow-up after the booster. As we had previously identified these clones and assessed each one independently, we did not use any multiple hypothesis adjustment and set a significance threshold of *P*_adjusted_ *<* 0.01 for boost expansion. Boost expansion was analysed in all patients with identified autogene-cevumeran-expanded clones, except for patient 5 who did not receive a booster.

### In vitro stimulation and T cell clone specificity to peptides

We resuspended neopeptides (Genscript) in DMSO at 10 mg ml^–1^ and a SARS-CoV-2 peptide pool (Mabtech) following the manufacturer’s instructions. We stored all peptides at –80 °C. We restimulated PBMCs with peptides in vitro as previously described with minor modifications^2^. In brief, we cultured 1 × 10^6^ PBMCs in a 48-well plate with individual (10 μg ml^–1^) or pooled (1–5 μg ml^–1^ per peptide) peptides on day 1. We added IL-2 (100 U ml^–1^) and IL-15 (10 ng ml^–1^) on day 2 and every subsequent 2–3 days. On day 7, we restimulated cells with peptides and added a CD107a antibody (clone H4A3, PE, BD Biosciences) for 1 h at 37 °C. After 1 h, we added a protein transport inhibitor containing monensin (BD Biosciences) and incubated for 4 h at 37 °C. We then stained the cells for additional surface or intracellular markers as per the manufacturer guidelines, and either analysed or purified cells based on CD107a surface expression, or analysed cells based on intracellular cytokine expression.

To determine whether a T cell clone was specifically stimulated by the peptide pool, we sorted and identified T cell clones in CD107a^–^ and CD107a^+^ fractions after peptide stimulation as described above. We then determined a peptide-specificity stimulation *P* value for each T cell clone using a one-tailed binomial test *P* value (implementing the scipy.stats.binom_test) with a 0.2 threshold (specifically, significance with respect to at least 20% of a clone being CD107a^+^ as opposed to CD107a^–^). We adjusted *P* values using Bonferroni correction and determined significance at a *P*_adjusted_ *<* 0.001 threshold. We included DMSO as a control to identify nonspecifically stimulated T cell clones. Of all patients tested, only one nonspecific clone was identified (patient 10) as nonspecifically stimulated in DMSO and both screened peptide pools. This clone was therefore excluded as a peptide-specific clone.

### HLA cloning and transduction

We cloned the HLA alleles into an SFG γ-retroviral vector^53^ and sequence-verified all plasmids (Genewiz). We transfected Phoenix-AMPHO cells with the plasmids using MegaTran 2.0 (OriGene). We collected virus-containing supernatants 48 h after transfection, added Polybrene (EMD Millipore) and spinoculated K562 cells for 2 h at 2,400 r.p.m. at 33 °C. Seventy-two hours after transduction, we sorted HLA^+^ K562 cells using an Aria Cell sorter (BD Biosciences).

### TCR cloning, transduction and peptide stimulations

We constructed TCR fragments as previously described^54^. In brief, we isolated TRB V-D-J and TRA V-J sequences from purified, sequenced single T cells and fused the TRB V-D-J and TRA V-J sequences to modified mouse constant TRB and TRA chain sequences^54^, respectively (gift from A. Gros) to prevent mispairing of transduced TCRs with the endogenous TCRs^55^. In brief, we joined TRB and TRA chains with a furin SGSG P2A linker, cloned the TCR constructs into a SFG γ-retroviral vector^53^ and sequence-verified all plasmids (Genewiz). We transfected H29 cells (gpg29 fibroblasts) with retrovirus vectors using calcium phosphate and produced VSV-G pseudo-typed retroviruses^45^. We next used Polybrene (Sigma) and virus-containing supernatants to generate stable RD114-enveloped producer cell lines^46^. We collected and concentrated virus-containing supernatants using a Retro-X Concentrator (Takara). We then coated non-tissue-culture treated 6-well plates with Retronectin (Takara), plated a titrated virus quantity to 3 × 10^6^ activated T cells per well, centrifuged cells for 1 h at room temperature at 300*g* and used transduced T cells between day 7 and 14 after transduction or cryopreserved them for future use. To stimulate TCR-transduced T cells with peptides, we pulsed 5 × 10^4^ (effector:target ratio 1:1) or 2.5 × 10^5^ (effector:target ratio of 1:5) HLA-transduced K562 cells (antigen presenting cells) in a 96-well U-bottom plate for 1 h at 37 °C with the indicated peptides at the indicated concentrations. After 1 h, we washed the peptide by centrifugation and added 5 × 10^4^ TCR or mock (control) transduced T cells per well. We then measured CD137 (also known as 4-1BB) expression on CD8^+^ mouse TCR^+^ T cells 24 h later.

### Immunophenotyping by flow cytometry and optical impedance

For flow cytometry, PBMCs from patients were rested overnight at 37 °C and 5% CO_2_ before staining^55^. We defined TCR-transduced CD8^+^ T cells as live, CD3^+^, CD8^+^, mouse TCR^+^ cells. We stained cells with the following antibodies: from BioLegend, CD62L (clone DREG-56, BV510), CD56 (clone HCD56, BV605), CD4 (clone OKT4, BV650), CD19 (clone HIB19, BV711), FOXP3 (clone 206D, PE), CD3 (clone SK-7, PE-Cy7), CD8 (clone SK1, FITC or Alexa Fluor 700), CD45RA (clone HI100, APC), CD45 (clone 2D1, Alexa Fluor 700), CD39 (clone A1, BV421), LAG-3 (clone 11C3C65, PerCP-Cy5.5), CD366 (clone F38-2E2, APC-Cy7) CD11c (clone S-HCL-3, BV421), HLA-DR (clone L243, BV785), CD14 (clone HCD14, PE), CD11b (clone ICRF44, APC), IFNγ (clone 4S.B3, BV421), mouse TRB (clone H57-597, PE-Cy5), CD137 (clone 4B4-1, PE), HLA-A,B,C (clone W6/23, APC) and a Zombie Red Fixable Viability kit (423110); from BD Biosciences, PD-1 (clone EH12.1, BV786), TNF (clone MAb11, APC), CD107a (clone H4A3, PE), CD56 (clone NCAM16.2, BV786) and DAPI (564907); from ThermoFisher Scientific, Ki-67 (clone SolA15, PE-Cy5). We stained cells using antibody cocktails in the dark at 4 °C, washed and analysed on a FACS LSR Fortessa (BD Biosciences) using FACSDiva (v.8.0.1) software (BD Biosciences). To examine the expression levels of intracellular markers, we surface-stained, fixed, permeabilized and stained the cells for intracellular proteins using a Fixation and Permeabilization Buffer kit per the manufacturer’s recommendations (Invitrogen). We used appropriate FMO controls as indicated. We analysed the data using FlowJo (v.10, Tree Star). We used the following definitions (all pre-gated on live, CD45^+^ cells): regulatory T cells, CD3^+^CD56^–^CD8^–^CD4^+^FOXP3^+^; dendritic cells, CD3^–^CD56^–^CD19^–^CD14^–^CD11c^+^HLA^–^DR^+^; monocytes, CD3^–^CD56^–^CD19^–^CD11b^+^CD14^+^; natural killer cells, CD3^–^CD56^+^; B cells, CD3^–^CD19^+^; CD8^+^ T cells, CD3^+^CD56^–^CD8^+^CD4^–^; CD4^+^ T cells, CD3^+^CD56^–^CD8^–^CD4^+^; natural killer T cells, CD3^+^CD56^+^. To identify frequencies of peripheral blood neutrophils, eosinophils and basophils, we measured respective cell frequencies by optical impedance on a clinical-grade Sysmex analyser.

### T cell sorting

We sorted bulk T cells from patient PBMC samples immediately after thawing on a BD FACS Aria flow cytometer (BD Biosciences). We sorted CD107a^–^ and CD107a^+^ CD8^+^ T cells after 7 days of peptide stimulation. We used the sorted T  cell samples for TCR Vβ sequencing, single-cell RNA/TCR sequencing, or single-cell TCR sequencing as indicated.

### Single-cell RNA/TCR sequencing

Library preparations for single-cell immune profiling, sequencing and post-processing of the raw data were performed at the Epigenomics Core at Weill Cornell Medicine.

#### Sample preparation

Single-cell RNA sequencing libraries were prepared according to 10x Genomics specifications (Chromium Single Cell V(D)J User Guide PN-1000006, 10x Genomics). Each cellular suspension (>90% viability), at a concentration between 700 and 1,000 cells per µl, was loaded onto to a 10x Genomics Chromium platform to generate Gel Beads-in-Emulsion (GEM), targeting about 10,000 single cells per sample. After GEM generation, polyA cDNA barcoded at the 5′ end by the addition of a template switch oligonucleotide (TSO) linked to a cell barcode and unique molecular identifiers (UMIs) was generated by incubation at 53 °C for 45 min in a C1000 Touch Thermal cycler with a 96-Deep Well Reaction module (Bio-Rad). GEMs were broken and the single-strand cDNA was cleaned up using DynaBeads MyOne Silane Beads (Thermo Fisher Scientific). The cDNA was amplified for 13 cycles (98 °C for 45 s; 98 °C for 20 s, 67 °C for 30 s, 72 °C for 1 h). Quality and quantity of the cDNA was assessed using an Agilent Bioanalyzer 2100, obtaining a product of about 1,600 bp. For generation of 5P expression libraries, an aliquot of the cDNA (about 50 ng) was enzymatically fragmented, end repaired, A-tailed, subjected to a double-sided size selection with SPRI select beads (Beckman Coulter) and ligated to adaptors provided in the kit. A unique sample index for each library was introduced through 14 cycles of PCR amplification using the indexes provided in the kit (98 °C for 45 s; 98 °C for 20 s, 54 °C for 30 s, and 72 °C for 20 s × 14 cycles; 72 °C for 1 min; held at 4 °C). Indexed libraries were subjected to a second double-sided size selection, and libraries were then quantified using Qubit fluorometric quantification (Thermo Fisher Scientific). The quality was assessed on an Agilent Bioanalyzer 2100, obtaining an average library size of 430 bp. For generation of full-length TCR VDJ regions, an aliquot of the cDNA (about 5 ng) was subjected to nested PCR amplification with specific VDJ outer and inner primer pairs (98 °C for 45 s; 98 °C for 20 s, 67 °C for 30 s, and 72 °C for 20 s × 8 cycles; 72 °C for 1 min; held at 4 °C), and one-sided size selection using SPRI select beads. Quality and quantity of the VDJ region was assessed using an Agilent Bioanalyzer 2100. The average library size was 620 bp.

#### Sequencing and post-processing of data

5P expression and TCR libraries were clustered on an Illumina NovaSeq pair-end read flow cell and sequenced for 28 cycles on R1 (10x barcode and the UMIs), followed by 8 cycles of I7 Index (sample Index), and 91 bases on R2 (transcript), obtaining about 250 million clusters for 5P expression and 50 million for TCR libraries. Primary processing of sequencing images was done using Illumina’s Real Time Analysis software (RTA). 10x Genomics Cell Ranger Single Cell Software suite (https://support.10xgenomics.com/single-cell-gene-expression/software/pipelines/latest/what-is-cell-ranger was used to perform sample) was used for demultiplexing, alignment (hg19), filtering, UMI counting, single-cell 5′ end gene counting, TCR assembly, annotation of paired VDJ and performing quality control using the manufacturer’s parameters.

#### Analysis

Filtered gene expression matrices generated from 10x CellRanger for five samples were matched to paired TCR sequences using the python package Scirpy^56^. All five samples were aggregated into a single unnormalized counts matrix and all downstream analyses were performed using GeneVector^57^. Batch-effect correction was applied over all cells using the samples as batch labels. Cells were first classified as either CD4 or CD8 T cells using the respective gene marker. CD8^+^ T cells were further classified into four phenotypes (effector, memory, naive and dysfunctional) using previously published gene markers^25,58^. A probability distribution over phenotypes was generated for each cell, and phenotype assignment corresponded to the maximum probability. Vaccine-specific T cells were identified by exact match of the associated nucleotide sequence. Uniform manifold approximation and projection visualizations were constructed using the python library Scanpy^59^.

### Clonality

Whole-exome sequence reads of tumour–normal paired samples of patients were aligned to the reference human genome (hg19) using the Burrows–Wheeler alignment tool (bwa mem v.0.7.17) and samtools (v.1.6). Duplicates were marked with picard-2.11.0 MarkDuplicates (http://broadinstitute.github.io/picard). Indel realignments were done using the Genome Analysis toolkit (GenomeAnalysisTK-3.8-1-0-gf15c1c3ef) RealignerTargetCreator and IndelRealigner^60^ using 1000 genome phase1 indel (1000G_phase1.indels.b37.vcf) and Mills indel calls (Mills_and_1000G_gold_standard.indels.b37.vcf) as references. Base calls were recalibrated using BaseRecalibrator and dbSNP (v.138). Both tumour samples were covered at 378× and normal samples at 346× on average on its target regions.

MuTect 1.1.7 and Strelka 1.0.15 were used to call SNV and indels on pre-processed sequencing data. For the MuTect calls, dbSNP 138 and CosmicCodingMuts.vcf (v.86)^61^ were used as reference files. For the Strelka calls, we set “isSkipDepthFilters = 1” to prevent filtering-out of mutation calls from exome sequencing due to exome-sequencing mapping breadth. Unbiased normal and tumour read counts for each SNV and indel call were then assigned with the bam-readcount software 0.8.0-unstable-6-963acab-dirty (commit 963acab-dirty) (https://github.com/genome/bam-readcount). A minimum base quality filter was set with the “-b 15” flag. The reads were counted in an insertion-centric way with the “-i” flag, so that reads overlapping with insertions were not included in the per-base read counts. We then used the normal and tumour read counts to filter mutations. The following filtering criteria were used: (1) total coverage for tumour ≥10; (2) variant allele frequency for tumour ≥2%; (3) number of reads with alternative allele ≥5 for tumour; (4) total coverage for normal ≥7; and (5) variant allele frequency for normal ≤1% at a given mutation. Filtered mutation sets were annotated using SnpEff (v.4.3t). 23 Dbsnp138 (b37) was used for snp-pileup.

To infer clonality of vaccine targets, we extracted missense and frameshift mutations from the filtered VCF files, and these mutations were put into the PhyloWGS software package (https://github.com/morrislab/phylowgs, v.1.0-rc2, branch: 681df79) along with copy number variant calls for phylogeny reconstruction. Among 10,000 trees from PhyloWGS, we took the top five trees based on the likelihood and computed the average entropy level to measure tumour heterogeneity. For a given tree, we computed exclusive clone frequencies such as

$${x}^{\alpha }={X}^{\alpha }-\sum _{\beta \in D(\alpha )}{X}^{\beta }$$

where $D(\alpha )$ is the set of clones that are direct descendants of clone $\alpha$ in the given tree, and ${X}^{\alpha }$ is the cellular cancer fraction of clone $\alpha$. Based on exclusive clone frequencies, we computed Shannon’s entropy as a measure of tumour heterogeneity as follows:

$$S={\left\langle -\sum _{\alpha \in \tau }{x}^{\alpha }{\rm{\log }}({x}^{\alpha })\right\rangle }_{\tau }$$

where ${\langle .\rangle }_{\tau }$ is the arithmetic average operator from top five trees ($\tau ).$

### Neoantigen quality and vaccine response

To model neoantigen quality, we adapted our previously described model^2,3^ that identified spontaneously targeted neoantigens in tumours to identify immunogenic neoantigens for vaccination. Specifically, according to our model^2,3^ the immunogenicity (or quality) of a neoantigen is the product of two components. The first component—the non-self-recognition potential, *R*, of a neoantigen—is the inherent immunogenicity of the neopeptide. The second component—the self-discrimination potential, *D*—models whether the cognate T cells of a neoantigen avoid negative thymic selection to therefore render neoantigen recognition less constrained by self-toleration.

Previous versions of our quality model^2,3^ estimated the non-self-recognition potential *R* of a neopeptide using sequence homology (as determined by soft max rescaling of BLAST alignment) to the immunogenic infectious disease-derived epitopes in the Immune Epitope Database (IEDB). Self-discrimination was estimated as a sum of two free discrimination energies between the neoantigen (MT) and its wild-type (WT) peptide, one for differential MHC presentation, the other for differential T cell cross reactivity:

$$D=\log \left(\frac{{K}_{{\rm{d}}}^{{\rm{WT}}}}{{K}_{{\rm{d}}}^{{\rm{MT}}}}\right)\,+\,| \log \left(\frac{{{\rm{EC}}}_{50}^{{\rm{MT}}}}{{{\rm{EC}}}_{50}^{{\rm{WT}}}}\right)| $$

where *K*_d_ is the HLA specific peptide–MHC affinity (as estimated by netMHC 3.4), and EC_50_ is the concentration for 50% activation for an avidity curve with the neopeptide and its cognate T cell clone^2,3^. Furthermore, in previous studies, we restricted our definition of minimal epitopes to consider to only 9-mers, the most common length of MHCI-bound peptides, predicted to bind to the HLA of the patient with a cut-off of 500 nM.

To now extend the notion of the cross-reactivity or epitope distance beyond the single substitution case as previously described^2,3^, we now made an independent site approximation by modelling the cross-reactivity distance, *d*_C_, between two 9-mer epitopes, *p*^A^ and *p*^B^ as follows:

$${d}_{{\rm{C}}}({p}^{{\rm{A}}},{p}^{{\rm{B}}})=\mathop{\sum }\limits_{i=1}^{9}{d}_{i}{M}_{{p}_{i}^{{\rm{A}}},{p}_{i}^{{\rm{B}}}}\approx | \log \left(\frac{{{\rm{EC}}}_{50}({p}^{{\rm{A}}})}{{{\rm{EC}}}_{50}({p}^{{\rm{B}}})}\right)| $$

where *d*_*i*_ is a scaling weight for position *i* and *M* is the substitution matrix as inferred from ref. ^3^. This extension allowed us to replace the estimation of the non-self-recognition potential *R* of a neopeptide from sequence homology using BLAST with epitope distance.

We now took as our two components, in the context of vaccination, how far a neopeptide is from the germline and how close it is to known antigenic IEDB epitopes. For a given 9-mer minimal neoepitope, *p*^MT^ we defined the quality of the 9-mer as follows:

$$Q({p}^{{\rm{M}}{\rm{T}}})={d}_{{\rm{C}}}({p}^{{\rm{M}}{\rm{T}}}\,,{p}^{{\rm{W}}{\rm{T}}})-\mathop{{\rm{\min }}}\limits_{{p}^{{\rm{a}}}\in {P}_{{\rm{I}}{\rm{E}}{\rm{D}}{\rm{B}}}}{d}_{{\rm{C}}}({p}^{{\rm{M}}{\rm{T}}}\,,{p}^{{\rm{a}}})$$

where *P*_IEDB_ is the collection of all 9-mers sequences and subsequences of IEDB epitopes.

We defined the quality of a neopeptide as the average quality over the two highest quality 9-mer subsequences that include the substituted residue and are predicted binders (threshold of 4,000 nM) to the HLA type of the individual. As a vaccine can induce neoantigen expression in excess of endogenous expression in a tumour, we dropped the differential MHC presentation term and relaxed our MHC binding cut-off.

To determine whether neoantigen quality correlated with immunogenicity of neoantigenic peptides included in the vaccines used in this study, we classified the neopeptides from the *n* = 8 immune responders as derived from immunogenic or non-immunogenic neoantigens according to the ELISpot assay. Individual immunogenicity was unable to be established for 7 out of the neoantigens from patient 25 and were excluded from the analysis. We used neoantigens only from immune responders to ensure that lack of an immunological response to a neoantigen reflected non-immunogenicity and not general vaccine failure. This generated 23 immunogenic neoantigens out of a total of 99 screened neoantigens from *n* = 8 immune responders. After excluding neoantigens with no predicted minimal epitope binders, we had a final cohort of 22 reactive neopeptides out of a total of 79.

### Immunofluorescence

Automated double immunofluorescence was conducted using the Leica Bond BX staining system. Paraffin-embedded tissues were sectioned at 5 μm and baked at 58 °C for 1 h. Slides were loaded in Leica Bond and staining was performed as follows. Samples were pretreated with EDTA-based epitope retrieval ER2 solution (Leica, AR9640) for 20 min at 95 °C. The double antibody staining and detection were conducted sequentially. Primary antibodies against CD3 (0.6 µg ml^–1^, rabbit, Dako, A0452) and CD8 (1/10, rabbit, Ventana (Roche), 790-4460) were used. The Leica Bond Polymer anti-rabbit HRP secondary antibody (Leica Biosystems, DS9800) was applied followed by Alexa Fluor tyramide signal amplification reagents (Life Technologies, B40953) or CF dye tyramide conjugates (Biotium, 92174) for detection. After CD3 staining, epitope retrieval was performed for denaturation of primary and secondary antibodies before CD8 antibody was applied. After the run was finished, slides were washed in PBS and incubated in 5 μg ml^–1^ 4′,6-diamidino-2-phenylindole (DAPI) (Sigma Aldrich) in PBS for 5 min, rinsed in PBS and mounted in Mowiol 4–88 (Calbiochem). Slides were kept overnight at −20 °C before imaging. Slides were scanned on a Panoramic scanner (3DHistech) using a ×20/0.8 NA objective. Whole tissues were annotated in CaseViewer (3DHistech) and converted to Tiff images. ImageJ was used to segment cells based on DAPI and to quantify whether a given cell is single, double or null positive.

### Humoral responses to SARS-CoV-2

We measured anti-SARS-CoV-2 spike IgG antibody titres with a chemiluminescent microparticle immunoassay (AdviseDx SARS-CoV-2 IgG II assay; Abbott). In brief, we combined serum samples with paramagnetic particles coated with recombinant SARS-CoV-2 protein specific for the receptor-binding domain of the S1 protein, followed by incubation, washing and addition of a conjugate and chemiluminescent substrate. We then measured the resulting chemiluminescent reaction as a relative light unit, with a direct relationship between the amount of IgG antibodies to SARS-CoV-2 in the sample and the relative light unit detected by the system optics (Architect i2000 analyzer). We used a 4 Parameter Logistic Curve fit data reduction method (4PLC, Y-weighted) to generate calibration, with a positivity cut-off of 50.0 AU ml^–1^.

### Circulating tumour DNA

We measured circulating tumour DNA using MSK-ACCESS^62^, a high-depth next generation sequencing assay with molecular barcoding technology for the detection of very low frequency somatic alterations in 129 key cancer-associated genes within the plasma cell-free DNA (cfDNA) fraction in peripheral blood. In brief, cfDNA MSK-ACCESS raw sequence data were demultiplexed and processed as previously described^62^. cfDNA samples were sequenced to a median raw coverage of 25,465× (range 5,007×–49,869×); after collapsing, the median duplex coverage was 1,088× (range 187×–1,783×). Variant calling was performed in a matched tumour-informed manner (“genotyping”) using GetBaseCountMultiSample (https://github.com/msk-access/GetBaseCountsMultiSample) and required at least 1 duplex consensus read or 2 simplex consensus reads, to call a somatic SNV or short indel at a site known to be altered in the matched tumour sample from a given patient, as previously described^62^.

### Digital droplet PCR

#### DNA extraction

Formalin-fixed paraffin-embedded (FFPE) curls collected in AutoLys M tubes (Thermo Fisher, A38738) were digested with protease solution. DNA was extracted using a MagMAX FFPE DNA/RNA Ultra kit (Thermo Fisher, A31881) on a KingFisher Flex purification system (Thermo Fisher) according to the manufacturer’s protocol. Samples were eluted in 55 µl elution solution.

#### Detection of *TP53*^R175H^ mutation by digital droplet PCR

*TP53* assays were ordered from Bio-Rad (assay identifiers dHsaCP2000105 for TP53 p.R175H c.524G>A; dHsaCP2000106 for TP53 WT). Cycling conditions were tested to ensure optimal annealing and extension temperatures and optimal separation of positive from empty droplets. Optimization was done using a known positive control.

After PicoGreen quantification, 9 ng of gDNA was combined with locus-specific primers, FAM-labelled and HEX-labelled probes, HaeIII and digital PCR Supermix for probes (no dUTP). All reactions were performed on a QX200 ddPCR system (Bio-Rad, 1864001), and each sample was evaluated in technical duplicates. Reactions were partitioned into a median of around 22,000 droplets per well using a QX200 droplet generator. Emulsified PCR assays were run on a 96-well thermal cycler using cycling conditions identified during the optimization step (95 °C for 10 min; 40 cycles of 94 °C for 30 min and 55 °C for 1 min; 98 °C for 10 min; 4 °C hold). Plates were read and analysed using QuantaSoft software to assess the number of droplets positive for mutant DNA, WT DNA, both or neither.

### Statistical analyses

Safety end points are presented descriptively as percentages. Sample sizes (*n*) represent the number of patients, tumours, T cell clones or neoantigens. We analysed feasibility as the statistical equivalence between benchmarked and achieved treatment times. Here, we defined a delay of <1 week as the zone of clinical indifference and defined the achieved time to be statistically equivalent to the benchmarked time if the 90% confidence interval of the achieved time was within the zone of clinical indifference. We analysed survival curves using log-rank (Mantel Cox) test, compared two groups using unpaired two-tailed Mann–Whitney test and categorical variables using Chi square test. We compared longitudinal clonal expansion using two-tailed Fisher’s exact test, and in vitro clonal activation using binomial test with Bonferroni correction. *P* < 0.05 was considered significant. All analyses were performed using GraphPad Prism (v.9.3.1) or Python (v.3.4).

### Reporting summary

Further information on research design is available in the Nature Portfolio Reporting Summary linked to this article.

## Online content

Any methods, additional references, Nature Portfolio reporting summaries, source data, extended data, supplementary information, acknowledgements, peer review information; details of author contributions and competing interests; and statements of data and code availability are available at 10.1038/s41586-023-06063-y.

### Supplementary information

Supplementary Data 1 Clinical trial protocol.

Reporting Summary

Supplementary Tables 1–4

Supplementary Table 5

### Source data

Source Data Fig. 1

Source Data Fig. 2

Source Data Fig. 4

Source Data Extended Data Fig. 1

Source Data Extended Data Fig. 2

Source Data Extended Data Fig. 3

Source Data Extended Data Fig. 4

Source Data Extended Data Fig. 5

Source Data Extended Data Fig. 6

Source Data Extended Data Fig. 7

Source Data Extended Data Fig. 8

Source Data Extended Data Fig. 9

Source Data Extended Data Fig. 10

## Figures and Tables

**Extended Data Table 1:**
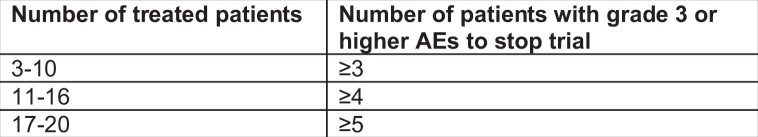
Primary Safety Endpoint

The number of patients with grade 3 or higher adverse events (AEs) due to atezolizumab and autogene cevumeran required to stop trial.

**Extended Data Table 2:**
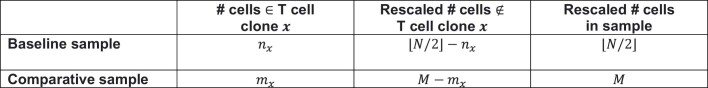
Repertoire rescaling for CloneTrack

## Data Availability

The clinical protocol approved by the institutional review board is provided in Supplementary Data 1. All single-cell sequencing data are available at the Gene Expression Omnibus (accession number GSE222011). De-identified individual participant data reported in the paper will be shared under data use agreements upon reasonable request. Requests must be made to balachav@mskcc.org. Source data are provided with this paper.

## Code availability

The following codes are available at GitHub: to infer clonality, https://github.com/genome/bam-readcount, https://github.com/mskcc/facets and https://github.com/morrislab/phylowgs (with details outlined in the Methods); to construct and apply the neoantigen quality model, https://github.com/LukszaLab/NeoantigenEditing; and for CloneTrack, https://github.com/zsethna/CloneTrack.
