# Supplementary material for: Personalized RNA neoantigen vaccines stimulate T cells in pancreatic cancer
Source: Nature. 2023 May 10;618(7963):144–50. doi: 10.1038/s41586-023-06063-y (PMC10171177; doi:10.1038/s41586-023-06063-y)
Supplement: Supplementary file 1 — Clinical trial protocol. [file 41586_2023_6063_MOESM1_ESM.pdf]

nature portfolio

https://doi.org/10.1038/s41586-023-06063-y

Supplementary information

Personalized RNA neoantigen vaccines stimulate T cells in pancreatic cancer

In the format provided by the authors and unedited

MSK PROTOCOL COVER SHEET

Phase 1 Clinical Trial of Personalized Neoantigen Tumor Vaccines and Programmed Death-Ligand 1 (PD-L1) Blockade in Patients with Surgically Resected Pancreatic Cancer

Principal Investigator/Department: Vinod Balachandran, MD/Surgery

Memorial Sloan Kettering Cancer Center
1275 York Avenue
New York, New York 10065

Table of Contents

TABLE OF CONTENTS **2**
1.0 PROTOCOL SUMMARY AND/OR SCHEMA **6**
2.0 OBJECTIVES AND SCIENTIFIC AIMS **7**
3.0 BACKGROUND AND RATIONALE **7**
3.1 Background on PD-L1 and Atezolizumab (Please refer to the most recent version of atezolizumab's Investigator Brochure) 8
3.1.1 Summary of nonclinical studies for atezolizumab 9
3.1.2 Summary of clinical studies for atezolizumab 9
3.1.3 Clinical pharmacokinetics and immunogenicity for atezolizumab 10
3.2 Rationale for Combination Atezolizumab, Neoantigen Vaccines, and mFOLFIRINOX 11
3.2.1 Summary of nonclinical studies for the combination of vaccines and anti-PD-L1 11
3.3 Background on RO7198457 (Personalized Cancer Vaccine) 13
4.0 OVERVIEW OF STUDY DESIGN/INTERVENTION **18**
4.1 DESIGN **18**
4.2 INTERVENTION **21**
4.3 Biomarker Assessments 22
4.3.1 Assessment of vaccine-specific immune responses 23
4.4 Tumor Tissue Specimen Assessments 23
5.0 THERAPEUTIC/DIAGNOSTIC AGENTS **23**
5.1 Oxaliplatin (Eloxatin) 23
5.2 Irinotecan(CAMPTOSAR) 24
5.3 Leucovorin 24
5.4 5-Fluorouracil (5-FU) (Efudex, Arucil, Carac, Fluroplex) 25
5.5 Atezolizumab 25
5.6 RO7198457 26
5.7 Concomitant Therapy 28
5.7.1 Permitted therapy 29
5.7.2 Systemic corticosteroids and TNF-α inhibitors 29
5.7.3 Prohibited therapy 29
6.0 CRITERIA FOR SUBJECT ELIGIBILITY **30**
6.1 SUBJECT INCLUSION CRITERIA **30**
6.2 SUBJECT EXCLUSION CRITERIA **31**
7.0 RECRUITMENT PLAN **33**
8.0 PRETREATMENT EVALUATION **34**
9.0 TREATMENT/INTERVENTION PLAN **35**

9.1 ADMINISTRATION 35
9.2 TREATMENT DURATION 36
9.3 TREATMENT SCHEDULE 36

10.0 EVALUATION DURING TREATMENT/INTERVENTION 36

11.0 TOXICITIES/SIDE EFFECTS 40

11.1 TOXICITY RELATED TO MFOLFIRINOX 40
11.2 TOXICITY RELATED TO ATEZOLIZUMAB 40
11.3 POTENTIAL RISKS ASSOCIATED WITH RO7198457 41
11.3.1 Systemic reactions during or after infusion of RO7198457 41
11.3.2 Immune-mediated adverse events 42
11.3.3 Lymphopenia 42
11.4 POTENTIAL FOR OVERLAPPING TOXICITIES WITH RO7198457 AND ATEZOLIZUMAB 43
11.5 MANAGEMENT OF PATIENTS WHO EXPERIENCE SPECIFIC ADVERSE EVENTS ASSOCIATED WITH RO7198457 43
11.5.1 Dose modifications with RO7198457 43
11.5.2 Treatment interruption with RO7198457 46
11.5.3 Management guidelines for specific adverse events with RO7198457 47
11.5.4 Discontinuation criteria 53
11.5.5 Infusion-related reactions and guidance on premedication 53
11.6 DEFINITION OF DRUG-LIMITING TOXICITY OF RO7198457 AND ATEZOLIZUMAB 54
11.7 TREATMENT DISCONTINUATION CRITERIA 55
11.8 STUDY TREATMENT DISCONTINUATION 56
11.8.1 Study treatment discontinuation visit 56
11.8.2 Survival and subsequent anti-cancer therapy follow-up 57
11.9 MFOLFIRINOX DOSE MODIFICATION SCHEDULE 57
11.10 PRESCRIBED THERAPY DURING STUDY PERIOD 63

12.0 CRITERIA FOR THERAPEUTIC RESPONSE/OUTCOME ASSESSMENT 63

12.1 TOXICITY ASSESSMENT 63
12.2 DETERMINATION OF RECURRENCE-FREE SURVIVAL 64
12.3 DEFINITIONS OF MEASURABLE AND NON-MEASURABLE DISEASE (PER RECIST V.1.1) 64
12.3.1 Measurable disease 64
12.3.2 Non-measurable disease 64
12.4 GUIDELINES FOR EVALUATION OF MEASURABLE DISEASE 64
12.5 DETERMINATION OF OVERALL SURVIVAL 65
12.6 DETERMINATION OF RO7198457 -SPECIFIC IMMUNOLOGIC CHANGES AT BASELINE AND AFTER THERAPY 65
12.7 IDENTIFY BLOOD AND TISSUE-SPECIFIC GENOMIC, TRANSCRIPTOMIC, AND IMMUNOLOGIC CHANGES WITH RO7198457 AND IMMUNE CHECKPOINT INHIBITION THERAPY 65

13.0 CRITERIA FOR REMOVAL FROM STUDY 65

14.0 BIOSTATISTICS 65

15.0 RESEARCH PARTICIPANT REGISTRATION AND RANDOMIZATION PROCEDURES 67

15.1 RESEARCH PARTICIPANT REGISTRATION 67

16.0 DATA MANAGEMENT ISSUES 67

16.1 QUALITY ASSURANCE 68
16.2 DATA AND SAFETY MONITORING 68
16.3 REGULATORY DOCUMENTATION 68
16.4 DOCUMENT MAINTENANCE 69

17.0 PROTECTION OF HUMAN SUBJECTS 69

17.1 PRIVACY 70

17.2 SERIOUS ADVERSE EVENT (SAE) REPORTING 70

***ASSESSMENT OF SAFETY* 70**

17.2.1 Serious adverse reporting to Genentech and BioNTech 76

CASE TRANSMISSION VERIFICATION OF SINGLE CASE REPORTS 76

18.0 INFORMED CONSENT PROCEDURES 80

19.0 REFERENCES 82

20.0 APPENDICES 86

APPENDIX 1. EASTERN COOPERATIVE ONCOLOGY GROUP (ECOG) SCALE OF PERFORMANCE STATUS[76] 86

APPENDIX 2. CLAVIEN-DINDO CLASSIFICATION OF POSTOPERATIVE COMPLICATIONS[68] 86

APPENDIX 3. PRE-EXISTING AUTOIMMUNE DISEASES 86

APPENDIX 4. RISKS ASSOCIATED WITH ATEZOLIZUMAB AND GUIDELINES FOR MANAGEMENT OF ADVERSE EVENTS ASSOCIATED WITH ATEZOLIZUMAB 88

APPENDIX 5. BIOMARKER MANAGEMENT PLAN (BMP) FOR IMCORE ISRS 110

FIGURES

Figure 1: Pictorial Protocol Schema 6
Figure 2. RNA-Lipoplex Combined with Anti-PD-L1 Leads to Increased Anti-Tumor Activity and Survival in TC1 Mouse Tumor Model 12
Figure 3. RNA-Lipoplex Combined with Anti-PD-L1 Leads to Increased Survival and Decreased Tumor Growth in a CT26 Mouse Model 12
Figure 4. RO7198457 Multi-Step Manufacturing Process 14
Figure 5. Schematic of mRNA 15
Figure 6. Schematic for Processing and Presentation of Neoantigens from Personalized Cancer Vaccine 15
Figure 7. Workflow Diagram 19

TABLES

Table 1 Protocol Schema 6
Table 2. Treatment Schema 21
Table 3. Administration of First and Subsequent RO7198457 Infusions 27
Table 4. Pretreatment Evaluation Schema 42
Table 5. Evaluations During Treatment 49
Table 6. Dose Modification Guidance for Flu-Like Symptoms/Influenza-Like-Illness 44
Table 7. Dose Modification Guidance for Infusion-Related Reactions 44
Table 8. Dose Modification Guidance for Cytokine Release Syndrome 46
Table 9. Guidelines for Management of Infusion-Related Reactions Related to Atezolizumab 48
Table 10. Guidelines for Management of Flu-Like Symptoms/influenza-Like Illness related to RO7198457 48
....54
Table 11. Guidelines for Management of Infusion-Related Reactions Related to RO7198457 49
Table 12. Guidelines for Management of Cytokine-Release Syndrome Related to RO7198457 62
Table 13. mFOLFIRINOX Dose Modifications 63
Table 14. Non-Hematologic Toxicity Dose Modification for Day 1 of Each Cycle 64
Table 15. Hematologic Toxicity Dose Modification for Day 1 of Each Cycle 65

1.0 PROTOCOL SUMMARY AND/OR SCHEMA

This is an open-label, Phase 1 study of sequential administration of adjuvant programmed death-ligand 1 (PD-L1) inhibitory antibody, personalized neoantigen vaccines, and modified FOLFIRINOX (mFOLFIRINOX) in subjects with resectable pancreas cancer. The objective and primary endpoint of this study is to assess the safety of this combination treatment. The secondary endpoints of this study are recurrence-free survival (RFS) and overall survival (OS). Exploratory scientific correlates include preliminary assessments of vaccine-induced immunity and the relative immunogenic potential of neoantigens of varying qualities.

The anticipated time to project completion is 2.5 years.

Table 1 Protocol Schema

Surgical resection of radiographically or histologically suspicious PDAC
Pathologic confirmation of resected tumor as PDAC
WES of resected tumors followed by RO7198457 (personalized neoantigen RNA Vaccine, iNeST) manufacturing
Atezolizumab (PD-L1 inhibitory antibody)
RO7198457 administration (9 total doses)
mFOLFIRINOX (12 cycles)
Surveillance imaging and serologic tumor markers

PDAC, pancreatic ductal adenocarcinoma; WES, whole-exome sequencing; PD-L1, programmed death-ligand 1; iNeST, individualized neoantigen specific therapy

Figure 1: Pictorial Protocol Schema

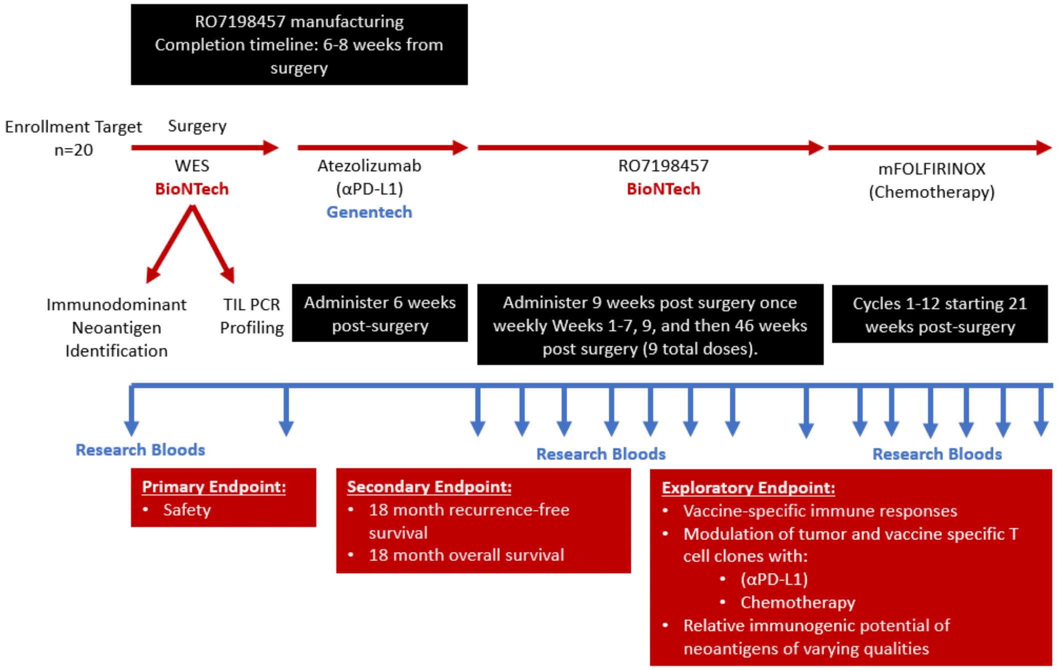

WES, whole-exome sequencing; TCR seq, T-cell receptor sequencing; TIL, tumor infiltrating lymphocytes; αPD-L1, anti-programmed death-ligand 1; PBMC, peripheral blood mononuclear cells

2.0 OBJECTIVES AND SCIENTIFIC AIMS

Primary objective:

- To determine the safety of sequential administration of atezolizumab and RO7198457 (an RNA-based individualized neoantigen specific therapy, iNeST), followed by standard of care mFOLFIRINOX. Safety is determined by a pre-specified percentage of subjects with a Grade 3 or higher drug-related adverse event (AE) or treatment termination due to a drug-related AE

Secondary objectives:

- To assess 18-month recurrence-free survival (RFS)
- To assess 18-month overall survival (OS)

Exploratory scientific correlates:

- To assess induction of vaccine-specific immune responses
- To evaluate cancer antigen (CA) 19-9 biomarker changes over time
  - To identify the most immunogenic neoantigens for clinical vaccination
  - To evaluate patterns of failure / disease recurrence
- To evaluate cell free DNA (cfDNA) at specified time points during patient's treatment and follow up
  - To track KRAS for recurrence

3.0 BACKGROUND AND RATIONALE

Pancreatic ductal adenocarcinoma (PDAC) is the fourth most common cause of cancer death in the U.S., with a 5-year OS rate of 7%.[1] PDAC is projected to become the second most common cause of cancer death by 2020.[2] The majority of patients initially present with distant metastasis or locally unresectable disease, accounting for the poor overall prognosis. Surgical resection is the only potentially curative therapy; however, only 20% of patients with PDAC present with a localized, resectable tumor.[3] Even after surgical resection, 5-year OS is only 12-27%[3-6], and most patients will experience disease recurrence at a median of 11.7 months.[3] Adjuvant chemotherapy remains the standard of care following surgery, however it has limited efficacy. The two primary adjuvant chemotherapy regimens remain mFOLFIRINOX and gemcitabine/nab-paclitaxel, based on efficacy of these agents in the metastatic setting[77, 78]. Gemcitabine monotherapy has shown to improve median OS from 20 months with no therapy, to 24 months.[7] However, although combination gemcitabine and nab-paclitaxel improved median OS to 8.5 months compared to 6.7 months with gemcitabine monotherapy in metastatic patients,[9] the APACT trial investigating this regimen in the adjuvant setting failed to improve its primary endpoint of DFS (press release, Celgene, May 2019). In contrast, mFOLFIRINOX has shown to improve survival in the adjuvant setting[79] and therefore is the standard of care in the adjuvant setting for patients < 75 year of age. Radiation therapy is largely ineffective in PDAC in prolonging survival. Given the ineffectiveness of all these above therapies, novel agents are urgently needed.

Immunotherapy agents blocking the inhibitory programmed death-1 (PD-1)/PD-L1 T-cell checkpoint have now demonstrated unprecedented response rates in patients with a wide array of treatment-refractory fatal cancers. Anti-programmed death-1/programmed death-ligand 1 (αPD-1/PD-L1) therapy has demonstrated dramatic response rates in advanced, treatment-refractory melanoma (28%), non-small cell lung cancer (NSCLC) (18%), renal cell carcinoma (RCC) (27%)[11], and hepatocellular cancer[12], and has shown efficacy in epithelial cancers such as colon[13], head and neck[14], gastric[15,16], and triple negative breast cancer (TNBC)[17], with durable responses even after drug discontinuation.[18] αPD-1/PD-L1 antibodies

are now FDA-approved to treat NSCLC, melanoma, urothelial cancer, RCC, non-Hodgkin lymphoma, mismatch repair-deficient colon cancer, gastric cancer, and hepatocellular cancer. Checkpoint blockade immunotherapy hence remains one of the most promising options to improve outcomes in conventional therapy-recalcitrant tumors such as PDAC.

Recent investigations by our group and others have demonstrated that checkpoint blockade immunotherapies boost T-cell responses against neoantigens generated by cancer-specific mutations.[19,20] Additionally, groups have shown that tumors resistant to αPD-1/PD-L1 such as PDAC can be rendered responsive to immunotherapy by combining αPD-1/PD-L1 with cancer vaccines, including neoantigen vaccines.[21-23] However, as neoantigens are unique to every patient, a concern in the implementation of neoantigen-directed therapies has remained the need for personalized genomic assessment in clinically relevant timeframes. Recent advances in next-generation sequencing and computational biology have now overcome these limitations, allowing rapid neoantigen identification and making clinical application of neoantigen-based immunotherapies feasible.[24] As a testament to this, 3 groups recently demonstrated that personalized neoantigen-vaccine approaches are feasible, safe, effective at generating anti-tumor T-cell responses, and associated with clinical benefit.[25-27] Furthermore, in preclinical models, vaccination with immunodominant neoantigens has shown to be as effective as checkpoint blockade immunotherapy in curtailing tumor growth.[28-30] Hence, as neoantigens are patient specific, tumor restricted, highly immunogenic, and not subject to natural mechanisms of T-cell tolerance, combining personalized neoantigen vaccines with αPD-L1 is a highly attractive, untested, therapeutic strategy in PDAC.

Two additional central questions to application of neoantigen-based therapies in PDAC remain:

1) Are neoantigens bona fide T-cell antigens in human PDAC?
2) How can we select a priori immunodominant neoantigens?

As such, our group has recently made significant progress in overcoming both of these barriers. We have developed a computational strategy to identify immunodominant neoantigens and demonstrated that neoantigen quality (a marker of tumors with the most immunodominant neoantigens) is predictive of response to immunotherapy. Additionally, in PDAC, although early reports posited that neoantigens were unlikely to be bona fide T-cell antigens[31,32], our recent work has demonstrated that immunodominant neoantigens are T-cell antigens in PDAC. Strikingly, patients whose tumors have the highest-quality neoantigens are the longest-term survivors, and evidence long-lasting persistent circulating T-cell immunity against these immunodominant neoantigens.[33] Consistent with this, neoantigen quality was prognostic of OS in 2 independent datasets of resected PDACs. Hence, given these striking findings and extensive data generated by our group in these areas of investigation, we believe we are now optimally positioned to apply these principles in a clinical context.

3.1 Background on PD-L1 and Atezolizumab (please refer to the most recent version of atezolizumab's Investigator Brochure)

PD-L1 is a co-inhibitory molecule expressed on the surface of tumor cells, tumor-infiltrating macrophages, and antigen-presenting cells (APCs). PD-L1 is a ligand for the inhibitory T-cell receptor (TCR) PD-1, which induces T-cell exhaustion, apoptosis, and anergy.[34] Antibody-mediated inhibition of the PD-1/PD-L1 axis has now shown dramatic responses in multiple solid tumors. However, many patients treated with PD-1/PD-L1 blockade alone do not experience sustained clinical benefit, underscoring the

need to explore immunotherapy combinations with the potential to overcome intrinsic or acquired resistance to checkpoint inhibition.[35] In PDAC, although PD-L1 expression was associated with worse OS in a study of 223 patients with resected PDAC[36], in a Phase 1 trial of an αPD-L1 antibody in metastatic solid tumors, there were no objective responses to monotherapy in the 14 patients with PDAC enrolled in the study; however, importantly, there was a highly favorable toxicity profile with only 9% of patients with a Grade 3 or higher toxicity due to αPD-L1.[21]

Atezolizumab is a human immunoglobulin G1 (IgG1) monoclonal antibody that binds to PD-L1, blocking its interaction with PD-1 and B7.1 (CD80) receptors on T-cells and APCs, respectively, abrogating PD-1 pathway-mediated T-cell inhibition. As a fully human IgG1 monoclonal antibody, atezolizumab is expected to be degraded into small peptides and amino acids via catabolic pathways in the same manner as endogenous immunoglobulin G (IgG). Atezolizumab is FDA-approved for locally advanced or metastatic urothelial carcinoma, first-line treatment of non-squamous NSCLC in combination with bevacizumab and chemotherapy, previously treated advanced, platinum-resistant NSCLC, locally advanced or metastatic TNBC, and first-line treatment, in combination with chemotherapy, for extensive-stage small cell lung cancer (ES-SCLC).[37,38] Atezolizumab will be supplied by Genentech (South San Francisco, CA).

3.1.1 Summary of nonclinical studies for atezolizumab

The nonclinical safety program demonstrated that weekly intravenous (IV) administration of atezolizumab at dose levels up to 50 mg/kg for up to 26 weeks (cynomolgus monkeys, only) was well tolerated in mice and cynomolgus monkeys. The nonclinical safety findings include neuropathy in C57BL/6 mice, vasculitis in several organs, and disturbed menstrual cycles in cynomolgus monkeys. The neuropathy and vasculitis are consistent with the anticipated pharmacologic activity of down-modulating the PD-L1/PD-1 pathway as well as identifying heightened immune responses and the potential to increase the frequency and/or the severity of immune-mediated AEs as possible safety risks to patients. These findings are consistent with the clinical safety profile, are considered to be amenable to monitoring, and are expected to be manageable.

3.1.2 Summary of clinical studies for atezolizumab

Relevant clinical data for atezolizumab are available mainly from 17 clinical trials in patients with solid tumors and hematologic malignancies. For each of these studies, treatment and/or analyses are ongoing. These include the following studies:

- **Single-agent studies of atezolizumab:**
  - **PCD4989g**: Open-label, Phase 1a study of atezolizumab in patients with locally advanced or metastatic solid tumors or hematologic malignancies
  - **GO29625**: Single-arm, Phase 2 study of atezolizumab in patients with PD-L1-selected NSCLC
  - **GO28753**: Randomized, open-label, Phase 2 study in patients with locally advanced or metastatic NSCLC who have failed a prior platinum-containing regimen; patients in the control arm receive docetaxel alone
  - Additional monotherapy studies include JO28944, GO29293,

GO28754, and WO29074

- **Combination studies with atezolizumab:**
  - **GP28328**: Phase 1b study evaluating atezolizumab administered with bevacizumab and/or chemotherapy in patients with advanced solid tumors
  - **GP28384**: Phase 1b study evaluating atezolizumab administered with vemurafenib or vemurafenib plus cobimetinib in patients with previously untreated BRAF V600 mutation-positive metastatic melanoma
  - **RO7198457 (PS003210) and Atezolizumab (MPDL3280A)—Genentech, Inc.**
  - **GP28363**: Phase 1b study evaluating atezolizumab administered with cobimetinib in patients with locally advanced or metastatic solid tumors
  - **WO29074**: Randomized, open-label, Phase 2 study of atezolizumab administered as monotherapy or in combination with bevacizumab versus sunitinib in patients with inoperable, locally advanced, or metastatic RCC
  - Additional combination studies include GO29383, WP29158, GO29322, BP29428, GO29674, BP29435, and BP29392

In addition, several ongoing studies by external sponsors are investigating atezolizumab in combination with chimeric antigen receptor (CAR)-T-cell, vaccine, or viral vector-based therapies, as follows:

- **NCT02609984:** Open-label, Phase 2 randomized study evaluating the safety and efficacy of CMB305 (a dendritic cell [DC]-targeting viral vector expressing the NY-ESO-1 gene plus an NY-ESO-1 recombinant protein plus glucopyranosyl lipid adjuvant formulated in a stable emulsion [GLA-SE]) in combination with atezolizumab in patients with locally advanced, relapsed, or metastatic sarcoma
- **NCT02926833:** Open-label, Phase 1/2 study evaluating the safety and efficacy of KTE-C19, an autologous anti-CD19 CAR T-cell therapy in combination with atezolizumab in patients with refractory diffuse large B-cell lymphoma (DLBCL)
- **NCT0295636:** Open-label, Phase 2 study evaluating the safety and efficacy of CDX-1401 (NY-ESO-1 fusion protein vaccine) in combination with Poly-ICLC and atezolizumab in NY-ESO-1 positive patients with locally advanced or metastatic NSCLC

3.1.3 Clinical pharmacokinetics and immunogenicity for atezolizumab

The pharmacokinetics of atezolizumab monotherapy have been characterized in patients in Study PCD4989g at doses 0.01 mg/kg to 20 mg/kg every 3 weeks (Q3W), including the fixed dose 1200 mg (equivalent to 15 mg/kg). Exposure to atezolizumab increased dose proportionally over the dose range of 1 mg/kg to 20 mg/kg. While a subset of anti-drug antibody (ADA)-positive patients in Study PCD4989g receiving 0.3 to 3 mg/kg atezolizumab Q3W experienced a reduction of atezolizumab minimum serum concentration (Cmin) to below the pharmacokinetic (PK) assay lower limit of quantification (LOQ), patients receiving 10 to 20 mg/kg atezolizumab, including the fixed 1200 mg dose, maintained geometric mean Cmin that was in excess of both the LOQ and the target serum concentration of 6 µg/mL.

A Phase 1 population PK (popPK) analysis that included 472 patients from Studies PCD4989g and JO28944 described atezolizumab pharmacokinetics for the dose range 1 to 20 mg/kg with a linear 2-compartment disposition model with first-order elimination. The popPK analysis indicated that central compartment volume of distribution (V1) was 3.28 L and the volume of distribution at steady state (Vss) was 6.91 L in the typical patient. Further, the clearance (CL) of atezolizumab was 0.20 L/day and the terminal half-life (t½) was 27 days. Steady state was obtained after 6 to 9 weeks (2 to 3 cycles) of repeated dosing.

3.2 Rationale for Combination Atezolizumab, Neoantigen Vaccines, and mFOLFIRINOX

Improved OS and progression free survival in patients with advanced PDAC using combination therapy regimens such as FOLFIRINOX[39] and gemcitabine/nab-paclitaxel[9] has generated significant interest in testing these drug regimens in patients with localized, resectable PDAC (no direct comparisons between the two have been done to date).[10,40-42] However, despite the proven efficacy of these combination therapy regimens in advanced PDAC, most patients do not respond to treatment and cytotoxic chemotherapy is not curative.[9,39] Hence, alternative therapeutic modalities are actively being investigated, including immunotherapy.

Rationale for efficacy: In PDAC, over 90% of tumor cells express PD-L1, and patients with PD-L1-expressing tumors have worse outcomes. Hence, as T-cell immunity has been shown to be associated with improved outcomes in PDAC[33,43], these data suggest that the PD-1/PD-L1 pathway may induce T-cell immunosuppression in PDAC.[44-46] Although single-agent PD-1/PD-L1 inhibition has not been shown to be effective in advanced PDAC[21,47], preclinical studies show combining PD-1/PD-L1 inhibition with cancer vaccines can induce sustained tumor regressions.[21-23] These data build on our findings that immunodominant neoantigens can induce robust intratumoral and long-term T-cell responses in PDAC.[33] Hence, there is strong rationale to combine PD-1/PD-L1 pathway inhibition with neoantigen-based vaccines. Tumors of long-term PDAC survivors are heavily infiltrated by activated CD8+ T-cells and have robust responses to immunodominant neoantigens in primary tumors with lasting immunodominant neoantigen-specific T-cell immunity in the periphery, providing further rationale for immunodominant neoantigen-based vaccines for PDAC. Finally, mFOLFIRINOX, in addition to having a proven survival benefit in the adjuvant setting, has been shown to synergize with vaccine therapies in pre-clinical models of pancreatic cancer[80].These results provide strong rationale for combining αPD-L1, neoantigen vaccines, and chemotherapy in PDAC.

3.2.1 Summary of nonclinical studies for the combination of vaccines and anti-PD-L1

The efficacy of the combination of RNA-Lipoplex vaccination and αPD-L1 was studied in the HPV16-E7-expressing TC-1 mouse model. TC-1/luc tumor cells were inoculated into the right flank of C57BL/6 mice subcutaneously. Fourteen days after tumor inoculation, mice received one immunization with 40 µg mRNA vaccine targeting E7 in lipoplex (E7 $RNA_{(LIP)}$) or 40 µg irrelevant RNA in lipoplex (Control $RNA_{(LIP)}$). Three days after vaccination, mice received five injections with either 200 µg αPD-L1 antibody or matched isotype control every

3-4 days. Tumor growth and survival were monitored over a period of 150 days. The combination of the E7 RNA(LIP) with αPD-L1 resulted in a longer survival benefit than the monotherapies with RNA(LIP) or αPD-L1 (Figure 2).

Figure 2. RNA(LIP) Combined with Anti-PD-L1 Leads to Increased Anti-Tumor Activity and Survival in TC1 Mouse Tumor Model

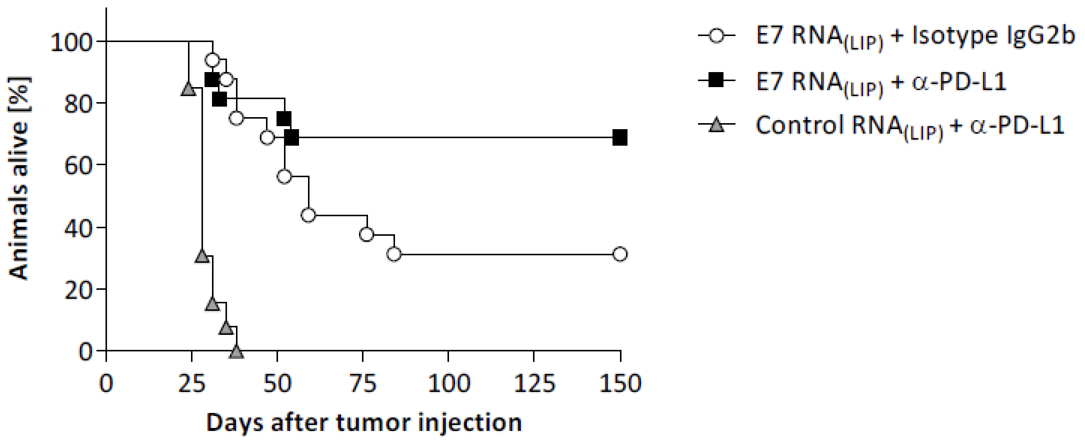

α-PD-L1, anti-programmed death-ligand 1; Ig = immunoglobulin
TC-1/luc tumor-bearing C57BL/6 mice (n = 13-16) were treated with E7 RNA(LIP) or α-PD-L1 antibody or a combination thereof. The Kaplan-Meier plot shows animal deaths for tumor-related reasons over time (150 days). α-PD-L1, anti-programmed death-ligand 1; Ig, immunoglobulin; Control RNA(LIP), irrelevant RNA(LIP); E7 RNA(LIP), RNA(LIP) targeting HPV16 oncoprotein E7.

In a second study, the efficacy of RNA-Lipoplex vaccination and αPD-L1 was studied in the CT26-mouse tumor model expressing the endogenous retroviral neoantigen gp70. BALB/c mice were injected with CT26 cells and treated (4 treatments each) with the following: (a) 20 µg RNA(LIP) targeting gp70 (gp70 RNA) or an irrelevant RNA; and (b) 200 µg of a mouse αPD-L1 antibody or an isotype control. The mice were treated with RNA(LIP) and antibody treatments concomitantly (i.e., both treatments on Days 9, 16, 23, and 30) or offset by 3 days (antibody treatment preceded RNA-Lipoplex by 3 days, i.e., days 6, 13, 20, and 27). This study shows that gp70 RNA(LIP) vaccination in combination with αPD-L1 antibody resulted in improved anti-tumor efficacy as measured by survival and tumor size compared to monotherapies (Figure 3). Moreover, gp70-specific T-cell frequency in blood was also increased upon combination treatment with gp70 RNA(LIP) and αPD-L1.

Figure 3. RNA-Lipoplex Combined with Anti-PD-L1 Leads to Increased Survival and Decreased Tumor Growth in a CT26 Mouse Model

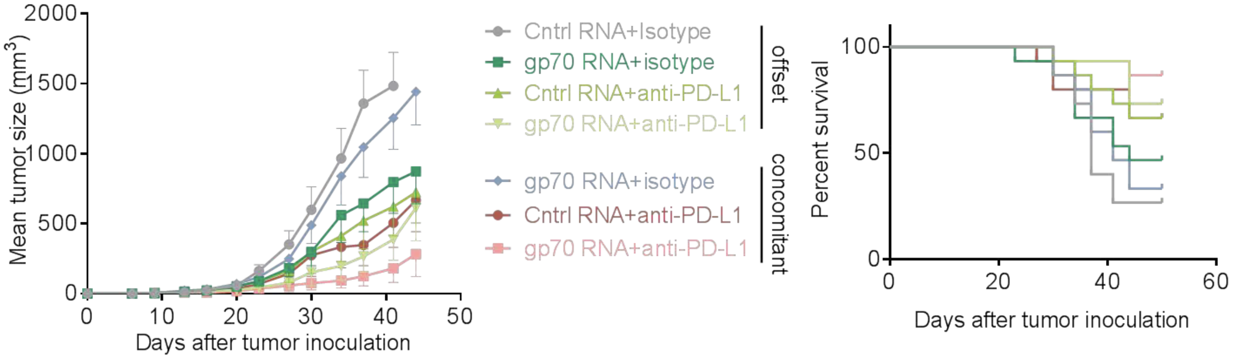

CT26 tumor-bearing C57BL/6 mice (n=15/group) were treated with gp70 RNA(LIP) or anti-PD-L1 antibody or a combination thereof
Left: Mean tumor growth ±SEM (last observation carried forward as long as N > 5 per group).
Right: Percentage of surviving mice is shown.
anti-PD−L1, anti-programmed death-ligand 1; Cntl RNA, irrelevant RNA(LIP); gp70 RNA, RNA(LIP) targeting endogenous retroviral neoantigen gp70

Dedicated combination toxicity studies with RO7198457 and atezolizumab have not been performed. No specific overlapping target organs were identified in the individual single-agent toxicology studies for RO7198457 or atezolizumab; however, these studies share the caveat that healthy, non-tumor bearing animals do not model the chronic antigen challenge expected in patients with advanced malignancies. Moreover, there were no overt toxicities noted in the combination efficacy studies of RO7198457 and αPD-L1 in mice. While the actual risk of combined toxicity is unknown, due to the similar pharmacological stimulatory effects of both agents on T-cell immunity, the potential exists for exacerbation of Immune-mediated toxicity, such as autoimmune disposition and systemic inflammatory responses, in patients treated concomitantly with both agents. Each agent had a low incidence and severity of drug-related adverse findings in nonclinical safety studies. Therefore, the anticipated toxicities from the combined administration of RO7198457 and atezolizumab are expected to be amenable to monitoring and manageable in the clinical setting. This is further supported by the clinical evaluation of vaccines with 2 other complementary immune checkpoint inhibitors (i.e., nivolumab and ipilimumab), which demonstrated a marginally increased frequency of Immune-mediated toxicity as compared with either single agent alone. However, most of those events were largely manageable and amenable to monitoring.[49,50] As a result, patients in the proposed Phase 1 trial will be closely monitored for evidence of immune-mediated AEs in addition to the standard monitoring for infusion reactions, altered clinical pathology parameters, and changes in their general health (see Section 5.0 THERAPEUTIC/DIAGNOSTIC AGENTS).

In summary, these data provide a strong rationale for combining RO7198457 with αPD-L1. The anti-tumor activity was observed with concomitant administration of vaccine and inhibition of PD-L1, even in models where single-agent vaccine had little activity.

3.3 Background on RO7198457 (Personalized Cancer Vaccine)

Most mutations are randomly induced by carcinogens, ultraviolet radiation, or DNA repair defects. As the resulting neoantigens are unique to each patient, targeting them through vaccination requires a personalized approach.[51] RO7198457 is a personalized cancer vaccine (PCV) that is based on the immunotherapeutic targeting of the unique mutations in a given patient's tumor. Multiple key technologies have been combined to generate a controlled process that covers all steps, from identification of mutations in individual clinical tumor specimens to the supply of an individually tailored RNA vaccine for use in a specific patient.

The manufacture of RO7198457 is a multi-step process (Figure 4), whereby somatic mutations in the patient's tumor are identified by next-generation sequencing and immunogenic neoantigens are predicted. The RNA cancer vaccine targeting the selected neoepitopes is manufactured on a per-patient basis. The investigational medicinal product (IMP) generated in this process is an RNA-based cancer vaccine consisting of up to 2 messenger RNA molecules, each encoding up to 10 neoepitopes (for a total of up to 20 neoepitopes), which are specific to the patient's tumor. A lipoplex nanoparticle formulation for the RNA (RNA-Lipoplex) is used to enable IV delivery of RO7198457. Neoantigen selection for personalized vaccine creation will be based on the BioNTech/Genentech (BioNTech, Mainz, Germany; Genentech, South San Francisco, USA) proprietary platform.

Figure 4. RO7198457 Multi-Step Manufacturing Process

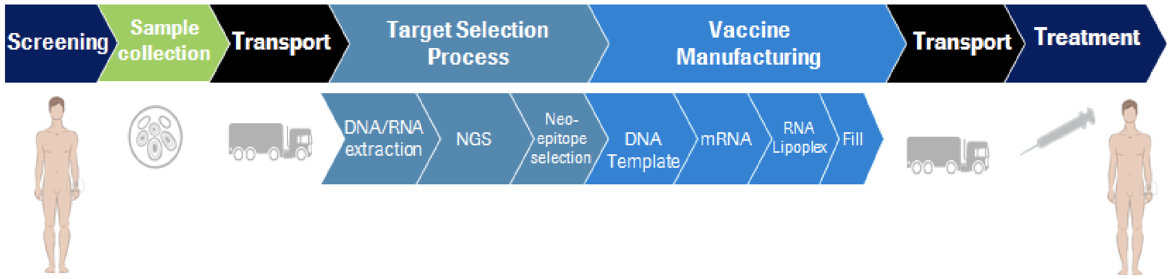

NGS, next-generation sequencing

There are 3 critical vaccine components that determine the type and strength of the immune response: 1) the antigens, 2) the delivery platform that brings antigens to DCs for presentation to T-cells, and 3) the adjuvant that provides the immunostimulatory signal to DCs that shapes the outcome of the immune response. These 3 critical components are all incorporated in RO7198457.

Expressed non-synonymous mutations are identified by whole-exome sequencing (WES) of tumor DNA and peripheral blood mononuclear cell (PBMC) DNA (as a source of healthy tissue from the patient) as well as tumor RNA sequencing (to assess expression). From the resulting list of mutant proteins, potential neoantigens are predicted using a bioinformatics workflow that ranks their likely immunogenicity on the basis of multiple factors, including the binding affinity of the predicted epitope to individual major histocompatibility complex class (MHC) molecules, the likelihood of presentation, the likelihood of binding by a T-cell, and the relative tumor clonal abundance.[33,52] Up to 20 MHC-I neoepitopes that are predicted to elicit CD8 T-cell immunity for an individual patient are selected for inclusion into the vaccine (Figure 5). Vaccinating against multiple neoepitopes is expected to increase the breadth and

magnitude of the overall immune response to the PCV and may help to mitigate the risk of immune escape, which can occur when tumors are exposed to the selective pressure of an effective immune response.[53,54]

Figure 5. Schematic of mRNA

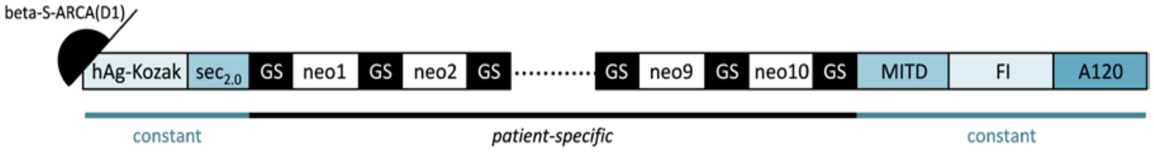

A lipoplex nanoparticle formulation for the RNA cancer vaccine comprising the synthetic cationic lipid (R)-N,N,N-trimethyl-2,3-dioleyloxy-1-propanaminium chloride (DOTMA) and the phospholipid 1,2-dioleoyl-sn-glycero-3-phosphatidylethanolamine (DOPE) is used to enable IV delivery of RO7198457. The DOTMA/DOPE liposomal component has been optimized for IV delivery and targeting of APCs in the spleen and other lymphoid organs. In vivo studies in mice demonstrated that the lipoplex formulation protects the RNA from degradation by extracellular ribonucleases and directs the RNA primarily to the spleen. The RNA-Lipoplex is internalized primarily by professional APCs including myeloid DCs, plasmacytoid DCs, and macrophages in the spleen for functional antigen presentation.[55]

The RNA molecule provides 2 of the functions critical to vaccine activity: 1) neoantigen expression and 2) adjuvant stimulus. Single-stranded RNA is a toll-like receptor (TLR) 7 and 8 agonist, and the RNA-Lipoplex compound is targeted to endosomal/lysosomal compartments in APCs where TLR7 and TLR8 are expressed. Plasmacytoid DCs express TLR7, while myeloid DCs express TLR8. Shared by many organisms, TLRs are important members of pattern recognition receptors (PRRs) that recognize conserved motifs called pathogen-associated molecular patterns (PAMPs), including TLRs, and are essential to stimulate immunity. TLR7/8 agonists are effective adjuvant stimuli for DCs, resulting in the enhancement of antigen presentation on MHC-I and MHC-II, the upregulation of costimulatory molecules, and cytokine production, including type I interferons (IFNs) and interleukin (IL)-12, that are necessary for the induction of T-cell responses. The production of type I IFNs has been shown to be important for T-cell priming and anti-tumor immunity.[56] Immunization studies in non-human primates showed that TLR7/8 agonists are potent adjuvants for induction of CD4 and CD8 T-cell responses.[57,58]

In myeloid DCs, the RNA is released into the cytosol and translated into a poly-neoepitopic peptide.[59] The polypeptide contains additional sequences to enhance antigen presentation. The signal sequence (sec) from the MHC-I heavy chain at the N-terminal of the polypeptide targets the nascent molecule to the endoplasmic reticulum, which has been shown to enhance MHC-I presentation efficiency.[60] The transmembrane and cytoplasmic domains of MHC-I heavy chain guide the polypeptide to the endosomal/lysosomal compartments that were shown to improve MHC-II presentation[60] (Figure 6).

Figure 6. Schematic for Processing and Presentation of Neoantigens from Personalized Cancer Vaccine

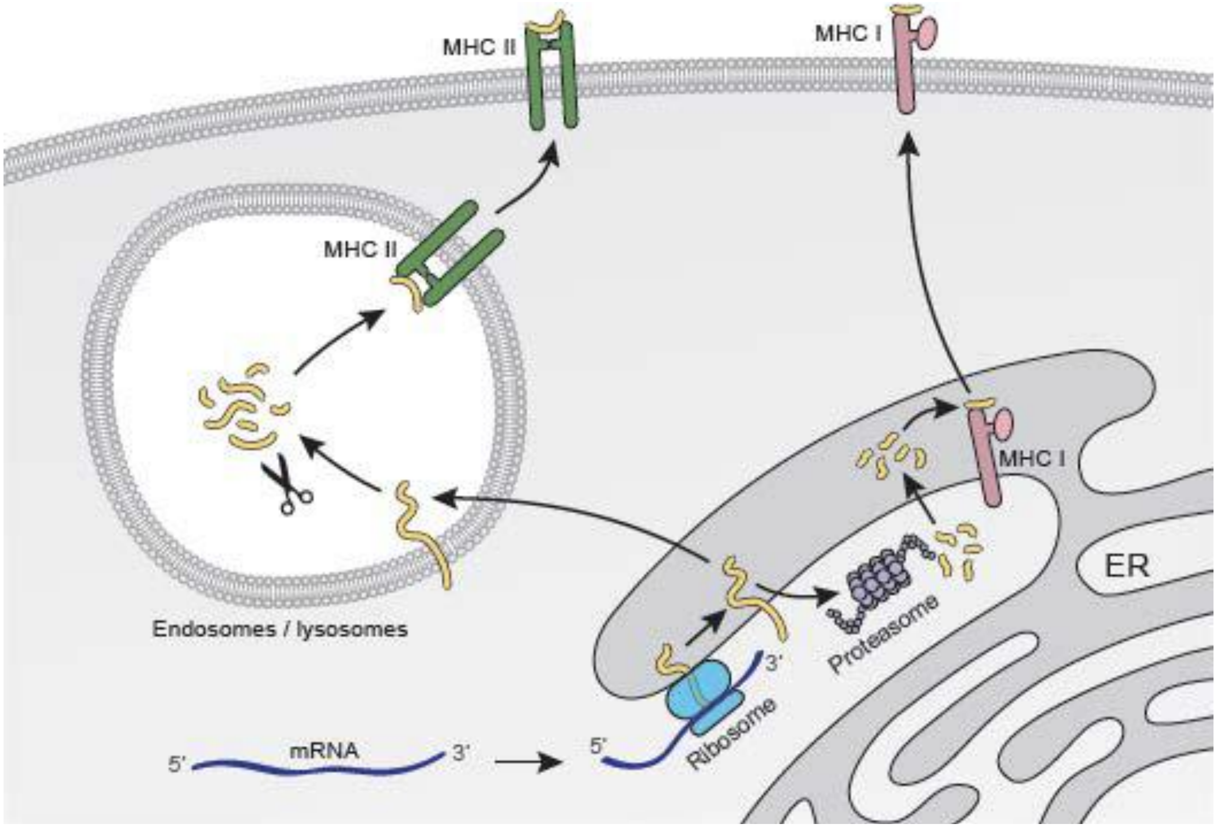

MHC-I, major histocompatibility complex class 1; MHC-II, major histocompatibility complex class 2; ER, endoplasmic reticulum

Rationale for safety: Relevant clinical data have been generated by the manufacturer of neoantigen vaccine (BioNTech Mainz, Germany) in 4 interventional, multicenter, first-in-human, open-label studies in patients with melanoma and TNBC. Please refer to the investigator brochure for details.

GO39733 (NCT03289962): This is a phase 1a/1b open label, dose escalation study of the safety and pharmacokinetics of RO7198457 as a single agent and in combination with atezolizumab in patients with locally advanced or metastatic tumors.

This study is currently ongoing with 307-770 patients planned for enrollment.

As of 28 February 2019, 25 patients have been enrolled in the Phase Ia single-agent portion and 85 patients (including 7 patients that have crossed over from Phase Ia) have been enrolled in the Phase Ib portion of Study GO39733. Phase Ia patients were enrolled into 5 different dose levels in dose-escalation, including backfill slots in dose levels that had cleared the DLT assessment period: 8 patients in the 25 μg RO7198457, 4 patients in the 38 μg RO7198457, 4 patients in the 50 μg RO7198457, 8 patients in the 75 μg RO7198457, and 1 patient in the 100 μg RO7198457 dose levels. In Phase Ib, all patients received RO7198457 in combination with 1200 mg atezolizumab. Patients were enrolled into four different Phase Ib dose levels in dose-escalation cohorts, including backfill slots in dose levels that had cleared the DLT assessment period: 10 patients in the 25 μg RO7198457, 9 patients in the 38 μg RO7198457, and 9 patients in the 50 μg RO7198457 dose levels. Patients were enrolled into two different dose levels in dose-exploration: 12 patients in the 15 μg RO7198457 and 7 patients in the 25 μg RO7198457. Patients were enrolled into different

indication specific cohorts in Phase Ib dose-expansion: 7 patients with melanoma, 5 patients with NSCLC, 6 patients with triple-negative breast cancer, 2 patients with renal cell cancer, and 11 patients with protocol defined tumor types in the serial biopsy cohort. In addition, 7 patients crossed over from Phase Ia portion and were treated in the Phase Ib portion of the study at 25 μg or 38 μg RO7198457 in combination with 1200 mg atezolizumab.

Among 103 patients, 25 enrolled in the Phase Ia portion and 85 (including 7 patients who have crossed over from Phase Ia portion) enrolled in the Phase Ib portion; all 25 patients (100%) enrolled in the Phase Ia portion and 85 patients (94%) enrolled in the Phase Ib portion had experienced at least one adverse event regardless of attribution to study drug. The most common adverse events, regardless of relationship reported in Study GO39733 in ≥ 25% of patients in Phase Ia, were infusion-related reaction (IRR; 52.0%, 13 patients), cytokine release syndrome (CRS; 32.0%, 8 patients), diarrhea (28.0%, 7 patients), fatigue (28%, 7 patients), and in Phase Ib were IRR (45.9%, 39 patients), and fatigue (29.4%, 25 patients).

In the Phase Ia portion, Grade ≥3 adverse events regardless of attribution occurred in 11 of 25 (44%) patients. Grade ≥3 adverse events assessed as related to the study treatment by the investigator were reported in 2 of 25 (8%) patients. These events included Grade 3 CRS (1 patient in 100 μg RO7198457 cohort) and Grade 3 fatigue (1 patient in 50 μg RO7198457 cohort). No Grade ≥ 3 events assessed as related to study drug were reported in the 25 μg, 38 μg, and 75 μg RO7198457 cohorts.

In the Phase Ib portion, Grade ≥ 3 adverse events regardless of attribution occurred in 31 of 85 (37%) patients. Grade ≥3 adverse events assessed as related to study treatment by the investigator were reported in 16 of 85 (19%) patients. These events included Grade 3 IRR (4 patients, 5%), Grade 3 lipase increased (3 patients, 4%), Grade 3 rash (2 patients, 2%), and Grade 3 autoimmune colitis, CRS, diarrhea, enterocolitis hemorrhagic, hypertension, hypotension, lymphocyte count decreased, non-cardiac chest pain, pemphigoid, pneumonitis, and systemic immune activation (1 patient each, 1%). The Grade 3 CRS resolved within 24 hours without sequelae.

No Grade 4 or Grade 5 adverse events that were assessed by the investigator to be related to the study drug(s) were reported in either the Phase Ia and Phase Ib portion of study GO39733.

As of 28 February 2019, one DLT was reported in the Phase Ia 100-μg RO7198457 cohort (Grade 3 CRS after the first infusion of RO7198457 at this high dose without prophylactic treatment). The patient recovered without sequelae.

As of the cut-off date, no DLTs were reported in Phase Ib.

The 25μg dose is also being evaluated in an ongoing phase II study of RO7198457 in combination with pembrolizumab (NCT03815058).

The MTD for RO7198457 has not yet been established. Based upon the safety profile of RO7198457 in combination with pembrolizumab, another immune checkpoint inhibitor, a phase 2 dose of 25 micrograms of RO7198457 was chosen and is currently being tested in combination with atezolizumab in a Phase 2 trial of advanced melanoma (NCT03815058). Given these safety data of RO7198457 in combination with pembrolizumab, and the ongoing phase 2 trial testing 25 micrograms of RO7198457 in combination with atezolizumab, we have chosen to test 25 micrograms of RO7198457 in combination with atezolizumab in our study

Additional rationale for safety with PD-L1 and chemotherapy: Cytotoxic T-lymphocyte-associated antigen 4 **(**CTLA-4) checkpoint inhibition using ipilimumab in combination with gemcitabine is safe in advanced PDAC (NCT02309177), and PD-L1 inhibition in combination with gemcitabine and nab-paclitaxel is currently being investigated in advanced PDAC (NCT02715531). Combination tumor vaccines and CTLA-4 checkpoint inhibition is safe in advanced PDAC[50], and combination neoantigen vaccines and PD-1 inhibition is safe in advanced melanoma.[26,27] Taken together, there is strong rationale that combining PDL-1 blockade and chemotherapy will be both effective and safe.

4.0 OVERVIEW OF STUDY DESIGN/INTERVENTION

4.1 Design

This is a Phase 1, open-label study of sequential administration of atezolizumab, personalized neoantigen RNA tumor vaccines, and mFOLFIRINOX in individuals with resectable PDAC. The overall study design is outlined Table 1 and Figure 1.

Patients with radiographic evidence of a primary, resectable pancreatic malignancy with features consistent with a ductal adenocarcinoma, and without evidence of distant metastatic spread, will be included in the study. Tumor resectability will be determined based on a dedicated pancreas-protocol contrast-enhanced computed tomography (CT) scan of the abdomen with 3-D reconstruction or MRI of the abdomen, with radiographic assessment of the relationship of the primary tumor to the mesenteric vasculature. Chest CT will be obtained to rule out distant metastatic disease. Radiographic tumor resectability will be defined as:

- no distant metastasis or extra-regional nodal disease
- a clear fat plane around the celiac and superior mesenteric arteries
- patent portal and superior mesenteric veins without tumor involvement
- no encasement of the superior mesenteric vein or portal vein involvement
- no encasement of the superior mesenteric or hepatic arteries

Subjects with metastatic PDAC, borderline resectable, or locally unresectable tumors will not be included in the study. All radiographic tests on study patients will be evaluated by 1 of 2 dedicated HPB radiologists (Dr. Richard Kinh Gian Do, MD, PhD; Dr. Seth Katz, MD, Department of Radiology).

A baseline serum CA 19-9 will be obtained on all study subjects.

Staging laparoscopy will be performed in the same setting as definitive surgical resection on all subjects. Tumors will be surgically resected, and the pathology will be confirmed as PDAC. All patients enrolled in the trial will be flagged for rapid pathologic review (Dr. Olca Basturk, MD, Department of Pathology). Tumor samples will be obtained from the tissue procurement services for RO7198457 manufacturing following which the resected samples will be submitted for complete pathologic analysis. The workflow for RO7198457 manufacturing is as follows, as outlined in Figure 7:

- DNA extraction (BioNTech; all unused samples, including unsequenced nucleic acid (DNA and RNA), will be returned to MSK)
- WES and mutation identification (BioNTech/Genentech)
- Neoantigen identification, prioritization, and selection for vaccines (BioNTech/Genentech)

- An input to the target selection process is the patient's HLA type. This is determined from an analysis of the patient's blood sample
- RO7198457vaccine manufacturing (BioNTech/Genentech)

Estimated timeline from tumor resection to RNA vaccine creation is 6-8 weeks.

Figure 7. Workflow Diagram

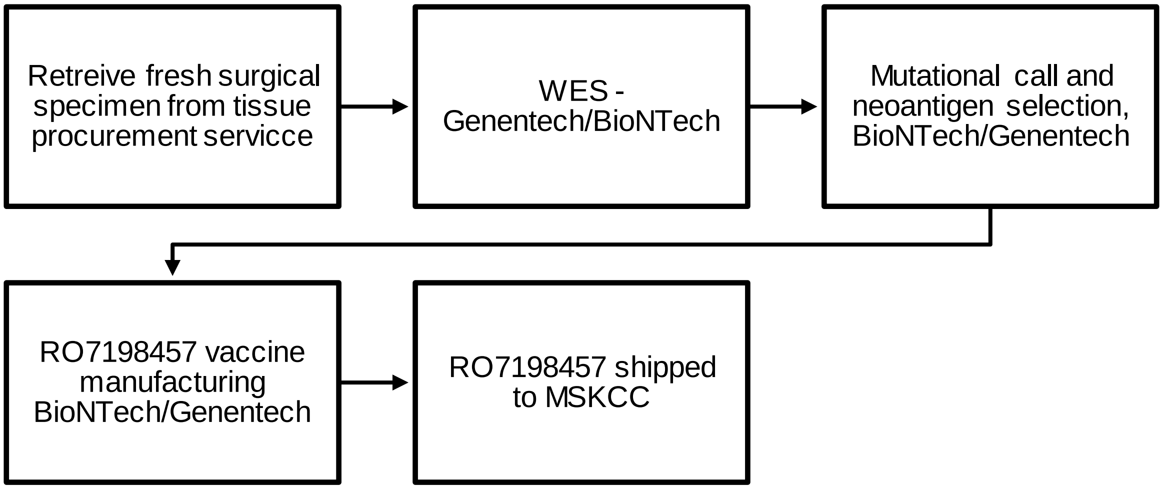

WES, whole-exome sequencing; MSKCC, Memorial Sloan Kettering Cancer Center

Approximately 6 weeks post-surgery, subjects will receive a single dose of atezolizumab 1200 mg IV (see Figure 1). The half-life of atezolizumab is 27 days with evidence of receptor occupancy persisting for several months (personal communication, Genentech)[61]. Based on findings from preclinical models treated with combination PD-L1 blockade and vaccines as detailed in Section 3.2.1 we hypothesize that this dosing schema will sufficiently allow for PD-L1 receptor occupancy prior to commencement of RO7198457 dosing. Our scientific hypothesis here is although PD-L1 inhibition has shown little efficacy as a single agent in metastatic PDAC, PD-L1 inhibition prior to RO7198457 administration will remove PD-L1 mediated T cell inhibition during RO7198457 administration, thereby leading to enhanced T cell priming with the RO7198457 is administered. Hence, a single dose may be able to achieve this. Furthermore, in a trial of personalized neoantigen melanoma tumor vaccines, 2 patients who received personalized vaccines had complete responses after administration of a single dose of a PD-1 inhibitor, suggesting that a single dose of a checkpoint inhibitor can be effective[27].

Starting approximately 9 weeks post-surgery, RO7198457 will be administered (Figure 1). RO7198457 will be administered at a dose of 25 µg once weekly for 7 doses, followed by an 8th dose 2 weeks later, and then a final dose 46 weeks after surgery (Figure 1). This RO7198457 administration schedule is supported by ongoing RO7198457 Phase 1 GO39733 trial in advanced cancers (NCT03289962).

Approximately 21 weeks post-surgery, subjects will receive 12 Cycles of mFOLFIRINOX as follows:

Oxaliplatin, at a dose of 85 mg per square meter delivered as a 2-hour intravenous infusion, followed by leucovorin, at a dose of 400 mg per square meter given as a 2-hour intravenous infusion, and after 30 minutes, the addition of irinotecan at a dose of 150 mg per square meter administered as a 30-90 minute intravenous infusion, immediately followed by fluorouracil at a dose of 2400 mg per square meter administered by continuous intravenous infusion over a period of 46 hours, every 14 days for 24 weeks (12 cycles). Dose administration logistics are per MSK guidelines. Cycles may also be reduced or omitted at the discretion of the treating clinician if deemed to be medically appropriate and the best interest of the patient. Such subjects may receive the final dose of the tumor vaccine approximately 3 weeks following the completion of the last cycle of chemotherapy.

Multiple studies specifically assessing the effect of delayed administration of adjuvant chemotherapy in patients with resected PDAC have demonstrated that there is no survival difference between subjects who receive chemotherapy <12 weeks or >12 weeks after resection[62-64]. Delaying receipt of adjuvant chemotherapy was carefully considered by the HPB Disease Management Team, and was considered to be reasonable, given that current evidence suggests that delaying administration of chemotherapy does not impact survival.

All adjuvant therapy regimens will be administered +/- 2 weeks of planned date of administration starting 6 weeks post-surgery.

Research blood draws will be performed at baseline, on the day of surgical resection, at post-op, prior to administration of atezolizumab, prior to administration of each dose of RO7198457, one week following the 8th dose of RO7198457, day 1 of the first two cycles of chemotherapy and each even cycle of chemotherapy thereafter, and 2 weeks following the completion of chemotherapy. Refer to lab manual for sample collection.

Serum CA 19-9 and surveillance cross-sectional imaging will be obtained at baseline following surgery prior to initiation of adjuvant therapies, and every 3 months after surgery during Postoperative Years 1 and 2, then every 6 months for Years 3-5, and then annually. Imaging will consist of CT chest/abdomen/pelvis with IV and oral contrast or MRI abdomen/pelvis and non-contrast CT chest.

Enrolled subjects will be monitored for drug-limiting toxicity throughout the study. A subject who withdraws from the study during the DLT assessment period for reasons other than a drug-limiting toxicity will be replaced (Section 13.0 CRITERIA FOR REMOVAL FROM STUDY). These patients will be followed for the full DLT evaluation period. The DLT assessment period begins from administration of atezolizumab until 30 days after completion of Cycle 12 of mFOLFIRINOX.

A total of 20 evaluable subjects will be enrolled. Subjects are considered evaluable once they enter the drug-limiting toxicity assessment window. Enrollment of the first 2 patients will be staggered by 2 weeks. If 3 or more subjects experience an Immune-mediated or RO7198457-related drug-limiting toxicity defined by a series of stopping rules based on the number of patients enrolled (Section 14.0 BIOSTATISTICS), enrollment will be stopped, and the data will be reviewed by the Disease Management Team (DMT) Safety Committee: Vinod P. Balachandran (PI), Eileen M. O'Reilly (Co-PI), Jedd D. Wolchok (Co-I), and T. Peter Kingham (Co-I), before any subject continues in the study. The DMT Safety Committee will review all drug-limiting toxicities and will determine if the study is safe to continue.

A drug-limiting toxicity is defined as a ≥ Grade 3 drug-related AE (see Section 11.6) occurring from administration of atezolizumab until 30 days after completion of Cycle 12 of

mFOLFIRINOX, excluding a transient Grade 3 infusion related reaction AE, using the National Cancer Institute (NCI) Common Terminology Criteria for Adverse Events (CTCAE) version 5.0. A drug-limiting toxicity will be considered related to the study regimen unless there is a clear, well-documented alternative explanation for the AEs. AEs ascribed to mFOLFIRINOX will not be considered a drug-limiting toxicity. See Section 11.6 (Definition of Drug-Limiting Toxicity of RO7198457 and Atezolizumab) for the full definition of drug-limiting toxicity.

Dosing in a patient will be halted based upon the occurrence of a ≥ Grade 3 drug-related AE and drug-limiting toxicities, beginning with administration of atezolizumab until 30 days after completion of Cycle 12 of mFOLFIRINOX. Delayed drug-limiting toxicities will be collected and evaluated by the Primary Investigator and will be used to assess the overall safety of the study regimen. If the number of ≥ Grade 3 drug-related adverse events and DLT's cross the predefined stopping rules (as defined in section 11.6), accrual will be held pending safety analysis and will be restarted only with review of the DMT Safety Committee. A delayed AE is defined as an DLT (see Section 11.6 Definition of Drug-Limiting Toxicity of RO7198457 and Atezolizumab for the full definition of drug-limiting toxicity) occurring >30 days after the DLT assessment period (30 days after completion of Cycle 12 of mFOLFIRINOX). Toxicity assessments will continue for the duration of adjuvant therapy administration.

Surveillance cross-sectional imaging evaluation will be obtained to assess for disease recurrence. RFS will be calculated based on the time from surgical resection until radiographically confirmed development of a new lesion. Subjects who develop a new lesion on surveillance imaging will undergo biopsy and WES of the locally recurrent or metastatic lesion. Subjects with development of tumor recurrence during receipt of adjuvant therapy, but with otherwise stable or improved performance/clinical status, may receive RO7198457 and chemotherapy according to the protocol schema for 2 additional cycles. At the next scheduled imaging evaluation, if there is further increase in the sum of the longest diameter or development of additional new lesions, then the subject will stop treatment and complete off-trial treatment.

Table 2. Treatment Schema

**Tumor Resection**
**Atezolizumab*** ~6 weeks post-tumor resection‡
RO7198457† ~9 weeks post-tumor resection‡
**mFOLFIRINOX**§ ~21 weeks post-tumor resection‡

*Atezolizumab 1200 mg IV (Single dose)
‡All therapies will be administered +/- 2 weeks of the indicated above time frames
†25 μg of total liposomal RNA IV once weekly for 7 doses, an 8th dose 2 weeks later, and then a final dose 46 weeks after surgery
§mFOLFIRINOX regimen will consist of oxaliplatin, at a dose of 85 mg per square meter delivered as a 2-hour intravenous infusion, followed by leucovorin, at a dose of 400 mg per square meter given as a 2-hour intravenous infusion, and after 30 minutes, the addition of irinotecan at a dose of 150 mg per square meter administered as a 30-90 minute intravenous infusion, immediately followed by fluorouracil at a dose of 2400 mg per square meter administered by continuous intravenous infusion over a period of 46 hours, every 14 days for 24 weeks (12 cycles). Dose administration logistics are per MSK guidelines.

4.2 Intervention

All subjects with a radiographically resectable pancreas tumor will undergo surgery. Staging laparoscopy will be performed during the same operative procedure as definitive resection to

confirm absence of distant metastatic disease. Following surgery, PDAC will be confirmed histologically. A single dose of atezolizumab will be administered approximately 6 weeks postoperatively following recovery. RO7198457 manufacturing will be based on selected tumor neoantigens from WES analysis performed on the surgically resected tumor specimen. Neoantigen selection and prioritization will be based on a proprietary platform (BioNTech, Mainz, Germany and Genentech, South San Francisco, USA). Approximately 9 weeks postoperatively, all subjects will begin adjuvant RO7198457 administration per the design schema indicated in Table 2. Three weeks after completing the first 8 doses of RO7198457, approximately week 21 postoperatively, subjects will receive 12 Cycles of mFOLFIRINOX. The mFOLFIRINOX regimen will consist of oxaliplatin, at a dose of 85 mg per square meter delivered as a 2-hour intravenous infusion, followed by leucovorin, at a dose of 400 mg per square meter given as a 2-hour intravenous infusion, and after 30 minutes, the addition of irinotecan at a dose of 150 mg per square meter administered as a 30-90 minute intravenous infusion, immediately followed by fluorouracil at a dose of 2400 mg per square meter administered by continuous intravenous infusion over a period of 46 hours, every 14 days for 24 weeks (12 cycles). The final (9th) dose of RO7198457 will be administered approximately 3 weeks following the completion of the 12th or the last chemotherapy dose. Dose administration logistics are per MSK guidelines. Surveillance cross-sectional imaging will be obtained every 3 months, starting in Postoperative Month 3. Additional imaging may also be obtained at the discretion of the treating clinician. A window of +/- 2 weeks for scans is allowed to accommodate subject schedules.

4.3 Biomarker Assessments

A variety of factors that could potentially predict clinical response to the protocol regimen will be investigated in peripheral blood and in tumor specimens taken from all subjects prior to treatment and on-treatment as outlined in the Protocol Schema (Section 1.0 PROTOCOL SUMMARY AND/OR SCHEMA). Data from these investigations will be evaluated for associations with safety (AE), treatment components (RO7198457 composition), and/or survival (OS, RFS). A window of +/- 2 weeks for research blood draws is allowed to accommodate subject clinical schedules as long as it is does not compromise the scientific integrity of the study.

All collected blood samples at MSKCC will be processed by the following protocol:

- Blood is stored at 20-25°C up to 4 hours before processing
- Phosphate-buffered saline (PBS) is added to blood samples to achieve a total volume of 50 mL
- 15 mL of Ficoll-Paque PLUS is added to the blood samples
- Samples are centrifuged at 1000g for 20 minutes at 21°C
- The layer containing PBMCs is harvested
- BEAD solution containing 500 mL of PBS and 5 mL of fetal bovine serum is added to the PBMCs to achieve a final volume of 50 mL
- Samples are centrifuged at 650g for 5 minutes at 4°C, and the supernatant is discarded
- 1-2 mL of BEAD is added to the remaining PBMCs, and samples are centrifuged at 250g for 10 minutes at 4°C
- The supernatant is discarded, and cells are resuspended in freezing media containing 90% fetal bovine serum and 10% dimethyl sulfoxide
- Cells are frozen in a -80°C freezer for 24 hours and then transferred to liquid nitrogen for long-term storage

Samples collected at MSKCC will be processed and subsequently stored in liquid nitrogen in the Vinod Balachandran Laboratory MSKCC, located on the 4th floor of the MSKCC Mortimer B. Zuckerman Research Center, 417 East 68th Street, New York, NY.

All samples will be labeled with the unique subject study identification number provided at registration, sample time point, and the date of the specimen.

Please see Appendix 5: Biomarker Management Plan (BMP) for imCORE ISRs for further details on correlative sample collection details, timepoints, and processing locations.

4.3.1 Assessment of vaccine-specific immune responses

Vaccine-specific immunity will be assessed by comparing the frequency of vaccine-reactive CD4+ and $CD8^+$ T-cells in pre-, during-, and post-vaccination blood specimens using cellular immunologic assays. Modulation of vaccine-specific immunity with chemotherapy will be assessed by comparing these readouts in pre- and post-chemotherapy blood samples. BioNTech/Genentech and the Vinod Balachandran Laboratory, MSKCC have extensive expertise in these assays.[33]

Neoantigen-specific immunity will be assessed by comparing the frequency of individual neoantigen-reactive T-cell clones (identified using T-cell receptor sequencing) in pre-, during-, and post-vaccination blood specimens. The Vinod Balachandran Laboratory, MSKCC has extensive expertise in these assays.[33]

4.4 Tumor Tissue Specimen Assessments

WES will be performed on the resected tumor specimens, and computationally predicted immunodominant tumor neoantigens will be determined (proprietary algorithm, BioNTech). These tumor samples may also be assessed for the expression of other immune or PDAC-related genes, RNAs and/or proteins, as well as the presence of immune cell populations using a variety of methodologies inclusive of, but not limited to, immunohistochemistry, flow cytometry, RNA, TCR, and single-cell sequencing. Various molecular markers with potential predictive value for the treatment of PDAC with atezolizumab and other immunotherapies are currently under investigation and may be assessed in this study. These tumor tissue biomarkers include, but are not limited to, PD-1, PD-L1, programmed death 1 ligand 2 (PD-L2), tumor infiltrating lymphocytes (TILs) or subpopulations of TILs and a Th1 immune mRNA expression signature. In addition, other methods of measuring tumor PD-L1 expression may also be assessed. Tissue from the resected tumors may be assessed for residual tumor cells and for markers expected to accompany tumor shrinkage in this study, including, but not limited to, TILs and subsets thereof. Tissue will be sent to Adaptive Biotechnologies for TCR Vb Sequencing on a quarterly basis.

5.0 THERAPEUTIC/DIAGNOSTIC AGENTS

5.1 Oxaliplatin (Eloxatin)

Oxaliplatin is a platinum-based drug used in combination with infusional 5-fluorouracil /leucovorin. Oxaliplatin undergoes nonenzymatic conversion in physiologic solutions to active derivatives via displacement of the labile oxalate ligand. Several transient reactive species are formed, including monoaquo and diaquo DACH platinum, which covalently bind with macromolecules. Both inter- and intrastrand Pt-DNA crosslinks are formed. Crosslinks are formed between the N7 positions of two adjacent guanines (GG), adjacent adenine-guanines (AG), and guanines separated by an intervening

nucleotide (GNG). These crosslinks inhibit DNA replication and transcription. Cytotoxicity is cell-cycle nonspecific.

Oxaliplatin is commercially available as Solution for Injection: 50 mg/10 mL (10 mL); 100 mg/20 mL (20 mL); 200 mg/40 mL (40 mL) and as Lyophilized Powder for Injection: 50 mg and 100 mg.

Please refer to package insert for complete preparation and dispensing instructions.

The most common adverse events associated with Oxaliplatin include: allergic reaction, fatigue, abdominal pain, skin disorders, injection site reaction, nausea, diarrhea, vomiting, stomatitis, anorexia, fever, infection, and overall peripheral sensory neuropathy. Please refer to package insert for full toxicity information.

5.2 Irinotecan(CAMPTOSAR)

Irinotecan (irinotecan hydrochloride injection) is an antineoplastic agent of the topoisomerase I inhibitor class. CAMPTOSAR is supplied as a sterile, pale yellow, clear, aqueous solution. Each milliliter of solution contains 20 mg of irinotecan hydrochloride (on the basis of the trihydrate salt), 45 mg of sorbitol, NF, and 0.9 mg of lactic acid, USP. The pH of the solution has been adjusted to 3.5 (range, 3.0 to 3.8) with sodium hydroxide or hydrochloric acid. CAMPTOSAR is intended for dilution with 5% Dextrose Injection, USP (D5W), or 0.9% Sodium Chloride Injection, USP, prior to intravenous infusion. The preferred diluent is 5% Dextrose Injection, USP.

Irinotecan is commercially available for injection 20 mg/mL (2 mL, 5 mL) [contains sorbitol 45 mg/mL; do not use in patients with hereditary fructose intolerance].

Please refer to package insert for complete preparation and dispensing instructions.

The most clinically significant adverse events for patients receiving irinotecan-based therapy were diarrhea, nausea, vomiting, neutropenia, and alopecia. Please refer to package insert for full toxicity information

5.3 Leucovorin

Leucovorin is a mixture of the diastereoisomers of the 5-formyl derivative of tetrahydrofolic acid (THF). The biologically active compound of the mixture is the (-)-l-isomer, known as Citrovorum factor or (-)-folinic acid. Leucovorin does not require reduction by the enzyme dihydrofolate reductase in order to participate in reactions utilizing folates as a source of “one-carbon” moieties. l-Leucovorin (l-5-formyltetrahydrofolate) is rapidly metabolized (via 5, 10-methenyltetrahydrofolate then 5, 10-methylenetetrahydrofolate) to l,5 methyltetrahydrofolate. l,5-Methyltetrahydrofolate can in turn be metabolized via other pathways back to 5,10-methylenetetrahydrofolate, which is converted to 5-methyltetrahydrofolate by an irreversible, enzyme catalyzed reduction using the cofactors FADH2 and NADPH

Leucovorin is commercially available as Solution for Injection 100 mg/10mL (10mL, 30 mL) and as Lyophilized Powder for Injection 50 mg, 100 mg, 200 mg, 350 mg, 500 mg. Leucovorin Calcium Injection USP is a sterile, preservative-free solution indicated for intramuscular (IM) or intravenous (IV) administration in a 50 mL single-dose vial. Each mL contains leucovorin calcium equivalent to 10 mg Leucovorin, USP; 8 mg sodium chloride; sodium hydroxide and/or hydrochloric acid for pH adjustment pH 7.8 (6.5 to 8.5).

The most common adverse events associated with Leucovorin include: Leukopenia, thrombocytopenia, infection, nausea, vomiting, diarrhea, stomatitis, constipation, lethargy, alopecia, dermatitis, and anorexia. Please refer to package insert for full toxicity information.

5.4 5-Fluorouracil (5-FU) (Efudex, Arucil, Carac, Fluroplex)

Fluorouracil injection, a nucleoside metabolic inhibitor, is a colorless to faint yellow, aqueous, sterile, nonpyrogenic injectable solution available in a pharmacy bulk package, a sterile preparation that contains doses for multiple patients for intravenous administration. Each mL contains 50 mg fluorouracil in water for injection, USP. The pH is adjusted to approximately 9.2 with sodium hydroxide.

Fluorouracil is commercially available as Intravenous Solution: 500 mg/10 mL (10 mL); 1 g/20 mL (20 mL); 2.5 g/50 mL (50 mL); 5 g/100 mL (100 mL).

Please refer to package insert for toxicity information.

5.5 Atezolizumab

Atezolizumab is a human IgG1 monoclonal antibody that binds PD-L1, blocking its interaction with PD-1 on T-cells, abrogating PD-1 pathway-mediated inhibition of the anti-tumor immunity. As a fully human IgG1 monoclonal antibody, atezolizumab is expected to be degraded into small peptides and amino acids via catabolic pathways in the same manner as endogenous IgG. Atezolizumab is FDA approved for advanced, cisplatin-resistant urothelial carcinoma,[37] triple negative breast cancer, first line non-small cell lung cancer, first line small cell lung cancer, and advanced, platinum-resistant NSCLC.[38] Atezolizumab will be supplied by Genentech.

Atezolizumab is a colorless to slightly yellow solution. Atezolizumab is prepared by aseptically withdrawing 20 mL from the manufacturer supplied vial and diluting the drug in a 250 mL polyvinyl chloride, polyethylene (PE), or polyolefin infusion bag containing 0.9% Sodium Chloride Injection, USP, and gently inverting the bag to mix the solution. Atezolizumab should be administered immediately once prepared. Diluted atezolizumab can be stored for 6 hours at room temperature or for 24 hours at 2°C-8°C (36°F-46°F). The solution should not be shaken or frozen.

The initial infusion of diluted atezolizumab is delivered over 60 minutes through an IV line with or without a sterile, non-pyrogenic, low-protein binding in-line filter (pore size of 0.2-0.22 micron). Do not co-administer other drugs through the same IV line. If clinically indicated, vital signs should be recorded during the infusion at 15, 30, 45, and 60 minutes (± 5 minutes for all time points) during the infusion and at 30 (± 10) minutes after the infusion. Subjects should be observed for 30 minutes after the infusion. Subjects should be informed about the possibility of delayed post-infusion symptoms and instructed to contact their study physician if they develop such symptoms.

The safety of atezolizumab has been demonstrated in the OAK trial including 609 subjects with NSCLC who received atezolizumab.[38] Overall, 390 (64%) subjects experienced a treatment-related AE, with 90 (15%) subjects experiencing a Grade 3 or 4 treatment-related AE. The most common AEs (greater than 2%) of atezolizumab include fatigue (26.8%), decreased appetite (23.5%), cough (23.2%), nausea (17.7%), diarrhea (15.4%), asthenia (19%), dyspnea (19.4%), anemia (11.5%), constipation (17.6%), pyrexia (17.7%), peripheral edema (8.9%), vomiting (12.2%), arthralgia (12%), myalgia (6.4%), back pain (11%), peripheral neuropathy (3.9%), musculoskeletal pain (10.5%), stomatitis (3%), and dysgeusia (3%). The most common Grade 3 or 4 adverse reactions (greater than 2%) were fatigue (2.8%), dyspnea (2.5%), and anemia (2.3%). As the median number of doses was 4 in this

trial, we anticipate a favorable safety profile with a single dose, as proposed in our above schema.

The AEs observed with atezolizumab in combination with chemotherapy and/or targeted therapies are consistent with the known risks of each study treatment.

The percentage of patients who discontinued atezolizumab due to any AE is consistent when used as a single agent or in combination with chemotherapy (e.g., 5.4% in Study PCD4989g [NCT01375842] and 5.8% in Study GP28328 [NCT01633970], respectively). The percentage of patients with any Grade 5 AE was similar when used as a single agent or in combination with chemotherapy (e.g., 1.6% in Study PCD4989g and 1.0% in Study GP28328).

Immune-mediated AEs are consistent with the role of the PD-L1/PD-1 pathway in regulating peripheral tolerance. Given the mechanism of action of atezolizumab, events associated with inflammation and/or Immune-mediated AEs are closely monitored during the atezolizumab clinical program. As of the Atezolizumab Investigator's Brochure Version 14 update (October 2018), Immune-mediated AEs associated with atezolizumab included hepatitis, pneumonitis, colitis, pancreatitis, diabetes mellitus, hypothyroidism, hyperthyroidism, adrenal insufficiency, Guillain-Barre syndrome, myasthenic syndrome/myasthenia gravis, meningoencephalitis, myocarditis, nephritis and myositis. Additionally, systemic immune activation is a potential risk with atezolizumab.

Fatigue, decreased appetite, nausea, and cough were commonly reported AEs in both single-agent and combination-therapy studies with atezolizumab. The overall Immune-mediated AEs reported for atezolizumab were considered moderate in severity, and the majority of patients were able to continue on atezolizumab therapy. Currently, no MTD, no DLTs, and no clear dose-related trends in the incidence of AEs for atezolizumab have been determined.

5.6 RO7198457

RO7198457 (an RNA-based individual neoepitope specific theapy, iNeST) will be individually manufactured by BioNTech/Genentech (Mainz, Germany and South San Francisco, USA), which will select and prioritize computationally predicted immunodominant tumor neoantigens identified from WES of PDAC surgical specimens. BioNTech/Genentech will return unused samples to MSK following the completion of sequencing. Transient, flu-like symptoms are the most common side effects of vaccine administration. Please refer to Section 3.3 and the Investigator Brochure for details regarding the RO7198457 safety profile.

After neoantigen prediction and selection, RO7198457 will be manufactured consisting of up to 2 messenger RNA molecules, each encoding up to 10 neoantigens (for a total of up to 20 neoantigens), which are specific to the subject's tumor. A lipoplex nanoparticle formulation for the RNA (RNA-Lipoplex) is used to enable IV administration. Vaccinating against multiple neoepitopes is expected to increase the breadth and magnitude of the overall immune response to RO7198457 and may help to mitigate the risk of immune escape, which can occur when tumors are exposed to the selective pressure of an effective immune response.[53,54]

A lipoplex nanoparticle formulation for the RNA cancer vaccine comprising the synthetic cationic lipid (R)-N,N,N-trimethyl-2,3-dioleyloxy-1-propanaminium chloride (DOTMA) and the phospholipid 1,2-dioleoyl-sn-glycero-3-phosphatidylethanolamine (DOPE) is used to enable IV delivery of RO7198457. The DOTMA/DOPE liposomal component has been optimized for IV delivery and targeting of APCs in the spleen and other lymphoid organs. In vivo studies in mice demonstrated that the lipoplex formulation protects the RNA from degradation by extracellular ribonucleases and directs the RNA primarily to the spleen. The RNA-Lipoplex is internalized primarily by professional APCs including myeloid DCs, plasmacytoid DCs, and macrophages in the spleen for functional antigen presentation.[55]

Administration of RO7198457 will be performed in a setting with access to a critical care staff who are trained to monitor for and respond to medical emergencies. The electronic medical record of all subjects enrolled in the trial will be flagged with the below administration instructions.

25 μg of total liposomal RNA IV per dose will be administered as outlined:

Table 3. Administration of First and Subsequent RO7198457 Infusions

Prior to Infusion	During and After the Infusion
• Pharmacologic measures (e.g., meperidine/pethidine, etc.) per local institutional standard for the prophylaxis of shivering, chills, or rigor may be used in patients who previously experienced Grade ≥2 shivering, chills, or rigor.<br>• Vital signs (pulse rate, respiratory rate, blood pressure, and temperature) should be recorded within 60 minutes prior to the infusion.<br>• RO7198457 should be administered by IV push.<br>• Hold antihypertensive medication per investigator discretion on the day of RO7198457 infusion for any patient who experienced Grade ≥3 hypotension with prior dose of RO7198457.	• Vital signs should be recorded at 30 (±10) minutes, 90 (±10) minutes, 4 hours (±10 minutes), and 6 hours (±10 minutes) after the infusion of RO7198457. Also for the first dose of each cycle, vital signs should be recorded 1 day after the infusion (±2 hours), and an appointment with a Clinical Trial Nurse (CTN) is encouraged. The 6-hour timepoint should only be done if patients are observed for 6 hours after dosing.<br>• Prophylactic treatment with antipyretics (e.g., acetaminophen 650-1000 mg, ibuprofen 400 mg) per institutional standard will be required within approximately 1 hour following all RO7198457 infusions.<br>• Ensure adequate hydration of patients on the day of RO7198457 administration. Administer IV isotonic fluid (e.g., normal saline 500-1000 mL) within approximately 1 hour following the dose of RO7198457 per institutional standard[a]<br>• Administer repeat doses of antipyretics (per institutional standard) every 4-8 hours, while the patient is being

Prior to Infusion	During and After the Infusion
	observed following RO7198457 infusion. Daily dose not to exceed the maximum recommended daily dose per label.<br>• Consider both non-pharmacologic (e.g., warming blankets, forced air patient warming system, etc.) and pharmacologic measures (e.g., meperidine/pethidine, tramadol) PRN for the mitigation and treatment of Grade ≥2 shivering, chills, or rigor per local institutional standard.<br>• Patients should be observed for approximately 6 hours after the end of the first infusion.<br>• For the second and subsequent infusions of RO7198457, patients will be observed for approximately 4-6 hours after each administration.<br>• Patients may be hospitalized overnight if required by institutional guidelines.<br>• If infusion-related adverse events (e.g., fever, chills) are not resolved to Grade ≤1 following outpatient observation period, patients should be admitted for overnight observation<br>• Patients should be informed about the possibility of delayed post-infusion symptoms and instructed to contact their study physician if they develop such symptoms.

[a] In case IV fluids are contraindicated (e.g., in patients with peripheral edema), IV hydration may be omitted

RO7198457 will be supplied by Genentech as a sterile liquid stored frozen in 10 mL glass vials. For information on the formulation, packaging, and handling of RO7198457; see the RO7198457 pharmacy manual and/or the RO7198457 Investigator’s Brochure.

Atezolizumab will be supplied by Genentech as a sterile liquid in 20 mL glass vials. The vial contains approximately 20 mL (1200 mg) of atezolizumab solution. For information on the formulation, packaging, and handling of atezolizumab, see the atezolizumab pharmacy manual and/or the Atezolizumab Investigator’s Brochure.

5.7 Concomitant Therapy

Concomitant therapy consists of any medication (e.g., prescription drugs, over-the-counter drugs, vaccines, herbal or homeopathic remedies, nutritional supplements) used by a subject in addition to protocol-mandated treatment from 7 days prior to screening to the study completion/discontinuation visit. All such medications should be reported to the investigator.

5.7.1 Permitted therapy

Patients who experience infusion-associated symptoms in this study may be treated symptomatically with acetaminophen, ibuprofen, diphenhydramine, and/or ranitidine or another H2 receptor antagonist, as per standard practice. Serious infusion-associated events manifested by dyspnea, hypotension, wheezing, bronchospasm, tachycardia, reduced oxygen saturation, or respiratory distress should be managed with supportive therapies as clinically indicated (e.g., supplemental oxygen and β2-adrenergic agonists). Premedication with antihistamines may be administered at the discretion of the treating physician after consultation with the DMT Safety Committee.

Systemic corticosteroids and tumor necrosis factor-α (TNF-α) antagonists may attenuate potential beneficial immunologic effects of treatment with RO7198457 in combination with atezolizumab, but may be administered at the discretion of the treating physician in an emergency or after consultation with the DMT Safety Committee. If feasible, short acting or intermediate acting corticosteroids (e.g., 80-240 mg hydrocortisone or 20-60 mg methylprednisolone, or) or alternatives to corticosteroids should be considered. Premedication may be administered at the discretion of the treating physician after consultation with the DMT Safety Committee. The use of inhaled corticosteroids and mineralocorticoids (e.g., fludrocortisone for patients with orthostatic hypotension or adrenocortical insufficiency) is allowed. Physiologic doses of corticosteroids for adrenal insufficiency are allowed. Megestrol administered as an appetite stimulant is also permitted. Planned use of other medications should be discussed with the DMT Safety Committee.

Patients who use oral contraceptives, hormone-replacement therapy, prophylactic or therapeutic anticoagulation therapy (such as low-molecular-weight heparin or warfarin at a stable dose level), or other maintenance therapy for non-malignant indications should continue their use. Males and females of reproductive potential should use highly effective means of contraception.

5.7.2 Systemic corticosteroids and TNF-α inhibitors

Systemic corticosteroids and TNF-α inhibitors may attenuate potential beneficial immunologic effects of treatment with atezolizumab and RO7198457. Therefore, in situations in which systemic corticosteroids or TNF-α inhibitors would be routinely administered, alternatives, including antihistamines, should be considered. If the alternatives are not feasible, systemic corticosteroids and TNF-α inhibitors may be administered at the discretion of the investigator, after DMT Safety Committee approval has been obtained, except that systemic corticosteroids may not be given as premedication to patients with an allergy to contrast agents used for tumor scans. Systemic corticosteroids are recommended, at the discretion of the investigator, for the treatment of specific AEs when associated with atezolizumab therapy.

5.7.3 Prohibited therapy

Use of the following therapies is prohibited during the study:

- Concomitant therapy intended for the treatment of cancer (including, but not limited to, chemotherapy [other than mFOLFIRINOX per study protocol], hormonal therapy, immunotherapy, radiotherapy, and herbal therapy), whether health authority-approved or experimental, is prohibited

during study treatment, until disease progression is documented, and the patient has discontinued study treatment

- Investigational therapy (other than protocol-mandated study treatment) is prohibited within 3 weeks prior to initiation of study treatment and during study treatment.
- Live, attenuated vaccines (e.g., FluMistTM [MedImmune, Gaithersburg, MD]) are prohibited within 4 weeks prior to initiation of study treatment, during atezolizumab treatment, and for 5 months after the last dose of atezolizumab
- Systemic immunostimulatory agents (including, but not limited to, IFNs and IL-2) are prohibited within 6 weeks or 5 half-lives of the drug (whichever is longer) prior to initiation of study treatment and during study treatment because these agents could potentially increase the risk for autoimmune conditions when given in combination with atezolizumab
- MAOIs including but not limited to phenelzine, selegiline, procarbazine, and tranylcypromine, are prohibited due to possible interactions with meperidine/pethidine causing serotonin syndrome. Any use of MAOIs should be discontinued at least 3 weeks before enrolling in the study.
- Immunosuppressive medications, including but not limited to cyclophosphamide, azathioprine, methotrexate, and thalidomide. These agents could potentially alter the activity and the safety of RO7198457 combined with atezolizumab
- Granulocyte colony-stimulating factors (e.g., granulocyte macrophage colony-stimulating factor [GM-CSF], and/or pegfilgrastim—prohibited during administration of atezolizumab or RO7198457, but allowed during mFOLFIRINOX)
- Patients are not allowed to receive immunostimulatory agents, including but not limited to IFN-α, IFN-γ, or IL-2, during the entire study. These agents, in combination with RO7198457 or atezolizumab, could potentially increase the risk for autoimmune conditions
- Traditional herbal medicines, because these are typically incompletely characterized and may result in unanticipated drug-drug interactions that can cause or confound assessment of toxicity
- Concomitant use of herbal therapies, including medical marijuana prescribed by a healthcare provider, is not recommended because their pharmacokinetics, safety profiles, and potential drug-drug interactions are generally unknown. However, medical marijuana/medical cannabis prescribed by a healthcare provider and not intended for the treatment of cancer but for the treatment of cancer-related conditions, such as chronic pain, muscle spasms, nausea, or anorexia, may be used during the study at the discretion of the investigator with Principal Investigator approval.

6.0 CRITERIA FOR SUBJECT ELIGIBILITY

6.1 Subject Inclusion Criteria

- Subjects must be ≥ 18 years of age at time of informed consent
- Able to comply with the study protocol, in the investigator's judgment
- Subjects with radiographically resectable primary pancreatic tumors with radiographic features consistent with adenocarcinoma will be evaluated for surgical resection
- Tumors must be radiographically resectable, defined as:

   - A clear fat plane around the celiac and superior mesenteric arteries
   - Patent superior mesenteric and portal veins without primary tumor involvement
   - No encasement of the superior mesenteric vein or portal veins
   - No encasement of the superior mesenteric orr hepatic arteries
   - No metastatic disease
   - No extra-regional nodal disease
- Subjects with histologically confirmed resected ductal pancreatic adenocarcinoma with macroscopic complete resection (R0 and R1) will be selected for neoantigen vaccine creation. Subjects with neuroendocrine (and mixed type) tumors are excluded
- Pancreatic cancer surgical staging: T 1-3, N0-2, M0
   - Per AJCC 8th edition staging
- Performance status of 0 or 1 on Eastern Cooperative Oncology Group (ECOG) Scale of Performance Status (Section 20.0 APPENDICES, Appendix 1)
- Subjects must not have had prior chemotherapy, radiation therapy, or immunotherapy for PDAC
- Subjects must be able to read, understand, and sign informed consent
- Women of childbearing potential must have a negative serum or urine pregnancy test within 14 days prior to study initiation
- For women of childbearing potential: agreement to remain abstinent (refrain from heterosexual intercourse) or use contraceptive measures that result in a failure rate of less than (<) 1% per year during the treatment period and for at least 5 months after the last dose of atezolizumab and for at least 90 days after the last dose of RO7198457. A woman is considered to be of childbearing potential if she is postmenarcheal, has not reached a postmenopausal state (≥ 12 continuous months of amenorrhea with no identified cause other than menopause), and has not undergone surgical sterilization (removal of ovaries and/or uterus)
- For men: agreement to remain abstinent (refrain from heterosexual intercourse) or use a condom during the entire study period and up to 90 days after last administration of RO7198457. Male participants should not donate sperm for 90 days after the last dose of RO7198457
- Examples of contraceptive methods with a failure rate of < 1% per year include bilateral tubal ligation, male sterilization, and established proper use of hormonal contraceptives that inhibit ovulation, hormone-releasing intrauterine devices, and copper intrauterine devices
- Hormonal contraceptive methods must be supplemented by a barrier method plus spermicide
- The reliability of sexual abstinence should be evaluated in relation to the duration of the clinical trial and the preferred and usual lifestyle of the patient. Periodic abstinence (e.g., calendar, ovulation, symptothermal, or postovulation methods) and withdrawal are not acceptable methods of contraception.

6.2 Subject Exclusion Criteria

A subject will not be eligible for inclusion in this study if any of the following criteria apply:

- Prior neoadjuvant treatment or radiation therapy for PDAC
- Prior therapy with αPD-L1 antibody or any other immune therapy
- Borderline resectable, locally unresectable or metastatic PDAC

- Pancreas tumor histology other than PDAC
- Pregnancy, breastfeeding, or intending to become pregnant during the study or within 90 days after the last dose of study treatment
- Life expectancy less than 12 weeks
- Inability to comply with study and/or follow-up procedures
- Any other malignancy for which the patient is undergoing active treatment which will be concurrent with the investigational agent in this study.
- Patients with unresolved Clavien-Dindo ≥ Grade 3 (Section 20.0 APPENDICES, Appendix 2) postoperative complications[68]
- Active, uncontrolled bacterial, viral, or fungal infection(s) requiring systemic therapy, defined as ongoing signs/symptoms related to the infection without improvement despite appropriate antibiotics, antiviral therapy, and/or other treatment
- Active tuberculosis
- Known infection with hepatitis B or C, or history of human immunodeficiency virus (HIV) infection, or subjects receiving immunosuppressive or myelosuppressive medications that would, in the opinion of the investigator, increase the risk of serious neutropenic complications
- Known hypersensitivity or allergy to the active substance or to any of the excipients in RO7198457, atezolizumab, oxaliplatin, leucovorin, irinotecan, or fluorouracil.
- Serious medical risk factors involving any of the major organ systems, or serious psychiatric disorders, which could compromise the subject's safety or the study data integrity. These include, but are not limited to:
  - History of connective tissue disorders (e.g., lupus, scleroderma, arteritis nodosa)
  - History of interstitial lung disease, slowly progressive dyspnea and unproductive cough, sarcoidosis, silicosis, idiopathic pulmonary fibrosis, pulmonary hypersensitivity pneumonitis, or multiple allergies
  - History of the following within 6 months prior to RO7198457 administration: a myocardial infarction, severe/unstable angina pectoris, coronary/peripheral artery bypass graft, New York Heart Association (NYHA) Class III-IV heart failure, uncontrolled hypertension, clinically significant cardiac dysrhythmia, or electrocardiogram (ECG) abnormality (exceptions: atrial fibrillation, paroxysmal supraventricular tachycardia), cerebrovascular accident, transient ischemic attack, or seizure disorder
- History of autoimmune disease, including but not limited to systemic lupus erythematosus, rheumatoid arthritis, inflammatory bowel disease, vascular thrombosis associated with antiphospholipid syndrome, Wegener's granulomatosis, Sjögren's syndrome, Bell's palsy, Guillain-Barre syndrome, multiple sclerosis, vasculitis, or glomerulonephritis (see Section 20.0 APPENDICES, Appendix 3, for a more comprehensive list of autoimmune diseases) with the following caveats:
  - Patients with a history of autoimmune hypothyroidism on a stable dose of thyroid replacement hormone may be eligible
  - Patients with controlled type 1 diabetes mellitus on a stable insulin regimen may be eligible
    - Patients type 2 diabetes mellitus may be eligible
  - Patients with eczema, psoriasis, lichen simplex chronicus, or vitiligo with dermatologic manifestations only (e.g., no psoriatic arthritis) may be eligible provided that they meet the following conditions:
    - Rash must cover less than 10% of the body surface area (BSA)

  - Disease is well controlled at baseline and only requires low potency topical steroids
  - No acute exacerbations of underlying condition within the last 12 months (e.g., not requiring psoralen and ultraviolet A [PUVA] radiation, methotrexate, retinoids, biologic agents, oral calcineurin inhibitors, high potency, or oral steroids)
- Treatment with systemic immunosuppressive medications (including but not limited to prednisone > 10 mg/day, cyclophosphamide, azathioprine, methotrexate, thalidomide, and TNF-α antagonists) within 2 weeks prior to RO7198457 administration. Patients who have received acute, low-dose, systemic immunosuppressant medications (e.g., a one-time dose of dexamethasone for nausea) may be enrolled in the study after discussion with and approval by the PI and Co-PI. The use of inhaled corticosteroids (e.g., fluticasone for chronic obstructive pulmonary disease) is allowed. The use of oral mineralocorticoids (e.g., fludrocortisone for patients with orthostatic hypotension) is allowed. Physiologic doses of corticosteroids for adrenal insufficiency are allowed.
- Subjects with allergies to IV contrast agents requiring pretreatment with corticosteroids will be excluded. Corticosteroids are immunosuppressive and may interfere with RO7198457 tolerability and efficacy. Given that there are serial contrast agent-dependent follow-up imaging studies built into the study which will overlap with vaccination, subjects who require pretreatment with corticosteroids prior to IV contrast administration will be excluded.
- History of idiopathic pulmonary fibrosis, pneumonitis (including drug induced), organizing pneumonia (i.e., bronchiolitis obliterans, cryptogenic organizing pneumonia, etc.), or evidence of active pneumonitis on screening chest CT scan. History of radiation pneumonitis in the radiation field (fibrosis) is permitted
- Known primary immunodeficiencies, either cellular (e.g., DiGeorge syndrome, T-negative severe combined immunodeficiency [SCID]) or combined T- and B-cell immunodeficiencies (e.g., T- and B-negative SCID, Wiskott-Aldrich syndrome, ataxia telangiectasia, common variable immunodeficiency)
- Prior allogeneic bone marrow transplantation or prior solid organ transplantation
- Any other diseases, metabolic dysfunction, physical examination finding, or clinical laboratory finding giving reasonable suspicion of a disease or condition that would contraindicate the use of an investigational drug
- Known clinically significant liver disease, including active viral, alcoholic, or other hepatitis, cirrhosis, and inherited liver disease or current alcohol abuse
- Previous splenectomy
- Administration of a live, attenuated vaccine within 4 weeks before RO7198457 administration or anticipation that such a live attenuated vaccine will be required during the study. Influenza vaccination should be given during influenza season only. Patients must not receive live, attenuated influenza vaccine (e.g., FluMist™) within 4 weeks prior to RO7198457 administration or at any time during the study, and for 90 days following the last study treatment

7.0 RECRUITMENT PLAN

All subjects meeting the eligibility requirements will be considered for enrollment regardless of sex, race, or religion. Subjects will be accrued from the HPB Service, GI Oncology Service, and Gastric/Mixed Tumor Service, and from both the MSKCC Department of Surgery and the MSKCC Department of Medicine. Eligibility criteria may not be waived by the investigator. Discussions regarding protocol enrollment and subject eligibility will begin with any of the investigators named on the consenting professionals list. Subjects will be made aware of the

protocol, its specific aims and objectives, and the potential risks and benefits the subjects may incur. Subjects will be required to read, agree to, and sign an institutional review board (IRB)-approved informed consent form prior to registration for this trial. Subjects will be consented prior to treatment initiation. There will be no financial compensation for subjects enrolling on this protocol. Our target accrual is 20 eligible subjects. Approximately 125 patients undergo resection for PDAC at MSKCC each year. Based on our experience with previous trials of this type, we expect an accrual time of approximately 1 year.

Potential patients will be screened, consented, and registered prior to surgery. Patients who cannot receive adjuvant therapy due to post-operative complications will be removed from the study in order to avoid unnecessary vaccine manufacture.

8.0 PRETREATMENT EVALUATION

Prior to treatment initiation, all subjects will undergo the following procedures:

- Cross-sectional imaging: pancreas-protocol CT abdomen/pelvis or MRI abdomen/pelvis and chest CT within 6 weeks of registration and 4 weeks of planned surgery. Separate CT scans of the chest, abdomen, and pelvis are acceptable, provided they are done within 6 weeks of registration and 4 weeks of surgery
- Research blood draw
- Hematology: complete blood count (CBC), including red blood cell (RBC) count, Hgb, hematocrit, platelet count, and WBC count with differential (neutrophils, eosinophils, lymphocytes, monocytes, basophils, and other cells)
- Chemistry panel (serum or plasma): sodium, potassium, chloride, bicarbonate, blood urea nitrogen (BUN) or urea, creatinine, glucose, calcium, magnesium, phosphorus, total bilirubin, ALT, AST, alkaline phosphatase, lactate dehydrogenase (LDH), total protein, albumin, amylase, and lipase
- Serum ferritin
- C-reactive protein (CRP)
- Coagulation: prothrombin time (PT), activated partial thromboplastin time (aPTT), and international normalized ratio (INR)
- Pregnancy test*:* All women of childbearing potential *(*including those who have had a tubal ligation). If a urine pregnancy test result is positive, dosing will be delayed until the patient's status is determined by a serum pregnancy test
- Urinalysis: dipstick (pH, specific gravity, glucose, protein, ketones, blood) and microscopic examination (sediment, RBCs, WBCs, casts, crystals, epithelial cells, bacteria) if warranted by dipstick results
- Thyroid function testing: thyroid-stimulating hormone (TSH), free T3, and free T4
- Serology:
    - Epstein-Barr nuclear antigen (EBNA) IgG
    - Cytomegalovirus (CMV) IgG
        - Hepatitis B surface antigen (HBsAg), antibodies against HBsAg, and hepatitis B core antigen. Hepatitis B virus (HBV) DNA test is to be obtained prior to Cycle 1, Day 1 if the patient has positive serology for total hepatitis B core antibody (anti-HBc)
        - Anti-hepatitis C virus (HCV). HCV RNA test is required prior to Cycle 1, Day 1 for consideration of eligibility if the patient has positive serology for anti-HCV
    - HIV antibodies

In addition, the following evaluations will be required at the times indicated in Section 10.0 Table 4.

9.0 TREATMENT/INTERVENTION PLAN

9.1 Administration

Adjuvant therapy will be administered to subjects as outlined in Table 2 Treatment Schema. Approximately 6 weeks after tumor resection, subjects will receive a single dose of atezolizumab 1200 mg IV. RO7198457 will be administered approximately 9 weeks after tumor resection once WES, antigen selection, and RO7198457 manufacturing is completed. RO7198457 will be administered weekly for 7 doses and then an 8th dose 2 weeks later, on RO7198457 Days 1, 8, 15, 22, 29, 36, 43, 57, and a final dose 46 weeks after surgery. Approximately 3 weeks after the 8th dose of RO7198457, subjects will commence 12 Cycles of mFOLFIRINOX. The mFOLFIRINOX regimen will consist of oxaliplatin, at a dose of 85 mg per square meter delivered as a 2-hour intravenous infusion, followed by leucovorin, at a dose of 400 mg per square meter given as a 2-hour intravenous infusion, and after 30 minutes, the addition of irinotecan at a dose of 150 mg per square meter administered as a 30-90 minute intravenous infusion, immediately followed by fluorouracil at a dose of 2400 mg per square meter administered by continuous intravenous infusion over a period of 46 hours, every 14 days for 24 weeks (12 cycles). Of note, for patients 76 years of age or older, there is an option to use adjusted started dosing (dose level -1) of mFOLFIRINOX. Dose administration logistics are per MSK guidelines.

The following parameters must be acceptable prior to initiation of adjuvant therapy:

- Acceptable hematology parameters:
  - White blood cell (WBC) count ≥ 2,500/µL
  - Absolute neutrophil count (ANC) ≥ 1500 cell/mm^3
  - Lymphocyte count ≥ 500/µL
  - Platelet count ≥ 100,000/mm^3
  - Hemoglobin (Hgb) ≥ 9 g/dL (Patients may be transfused or may receive erythropoietic treatment)
- Acceptable blood chemistry levels:
  - AST/Serum glutamic oxaloacetic transaminase (SGOT) and ALT/Serum glutamic pyruvic transaminase (SGPT) ≤ 2.5 × upper limit of normal range (ULN)
  - Serum albumin ≥ 2.5 g/dL
  - Serum creatinine within upper limits of normal or calculated clearance ≥ 50 mL/min/1.73 m^2. If using creatinine clearance, actual body weight should be used for calculating creatinine clearance (e.g., using the Cockcroft-Gault formula). For subjects with a body mass index (BMI) > 30 kg/m^2, lean body weight should be used instead
- Acceptable tumor sample criteria
  - Patients with confirmed availability of representative resection tumor specimens in formalin-fixed, paraffin-embedded (FFPE) blocks, or sectioned tissue (as described in the laboratory manual) with an associated pathology report
  - Multiple samples may be collected for a given patient, on the basis of availability; however, the requirement for a block or sectioned tissue should be satisfied by a single resection specimen.
  - A patient with insufficient or unavailable resection tumor tissue will not be evaluable due to the need for evaluable tumor tissue to manufacture RO7198457

- Enrollment will be limited to patients with at least five identified tumor neoantigens and sufficient tumor material (both quality and quantity) to manufacture vaccine.

9.2 Treatment Duration

Subjects will receive treatment until recurrence is noted, the subject develops unacceptable toxicity, there is a change in diagnosis, or until all treatment is administered (see Section 13.0 CRITERIA FOR REMOVAL FROM STUDY).

If oxaliplatin, leucovorin, irinotecan, or fluorouracil, atezolizumab, or RO7198457 is permanently discontinued due to disease recurrence, then the subject will be discontinued from the study, and may pursue alternative therapies.

If oxaliplatin, leucovorin, irinotecan, or fluorouracil, atezolizumab, or RO7198457 is held for reasons other than recurrence, subjects may continue on study protocol at the treating physician's discretion.

All subjects who consent to this study will be followed until their death, either in person at a clinic visit or over the phone.

9.3 Treatment Schedule

All reasonable efforts will be made to adhere to treatment and evaluation schedules; however, variations to accommodate holidays, transportation issues, or subjects' personal schedules will be permitted, provided they do not, in the opinion of the investigator, constitute major safety or compliance issues. Such variations, assuming they do not occur with unreasonable frequency or regularity, will not be considered protocol violations. A window of +/- 2 weeks is allowed for systemic treatments. If the subject requires a scan early for medical reasons, this will count as their protocol scan at the discretion of the investigator. A window of +/- 2 weeks is allowed for scans. If adjuvant therapy cannot be received because of postoperative complications, the subject will be removed from the study. If a subject does not undergo resection, the subject will be removed from the study.

10.0 EVALUATION DURING TREATMENT/INTERVENTION

Patients will be closely monitored for safety and tolerability and will be assessed for toxicity prior to each dose of RO7198457. Dosing will occur only if the clinical assessment and local laboratory test results are acceptable.

All assessments will be performed on the day of the scheduled visit date unless a time window is specified. Assessments scheduled on the days of study treatment should be performed before the infusion of study drug(s) unless otherwise noted. If the timing of a study visit coincides with a holiday, weekend, or other administrative disruption that precludes the visit, the visit should be scheduled on the nearest following feasible date, with subsequent visits rescheduled accordingly.

Evaluations during treatment are detailed in Table 4 below

Table 4. Evaluations During Treatment

	Prior to Surgery	2 weeks Post-op	Prior to αPD-L1	Approx. 6-9 weeks Post-op (after atezo and before R07198457)	Prior to Each RO7198457 Dose[1]	1 Day After RO7198457 Dose #1	Approx. 1 Week After RO7198457 Dose #8 (before C1D1 chemo)	Within 72 Hours prior to Day 1 of Cycles 1 & 2 and every even Cycle (beginning Cycle 4)[2]	Approx. 3 Weeks following completion of chemo (before R07198457 dose #9)	Every 3 Months After Resection Years 1-2	Every 6 Months Years 3-5, Then Annually
Toxicity Assessment			X		X			X		X	
Assessment of Tumor Resectability	X[8]										
Review of History Targeted Physical Exam	X		X[9]		X[14]			X		X	
Pregnancy test[10]	X[8]		X[9]		X			X			
ECOG Performance Status	X[8]		X[9]		X			X		X	
Ht/Wt[11]	X[8]		X[9]		X[11]						
PT	X[8]										
CBC with differential	X[8]		X[9]		X			X			
CMP	X[8]		X[9]		X			X			
Imaging[3]	X									X	X
CA 19-9	X[8]		X[9]		X			X		X	X[5]
cfDNA	X	X						X[6]		x	

	Prior to Surgery	2 weeks Post-op	Prior to αPD-L1	Approx. 6-9 weeks Post-op (after atezo and before R07198457)	Prior to Each RO7198457 Dose[1]	1 Day After RO7198457 Dose #1	Approx. 1 Week After RO7198457 Dose #8 (before C1D1 chemo)	Within 72 Hours prior to Day 1 of Cycles 1 & 2 and every even Cycle (beginning Cycle 4)[2]	Approx. 3 Weeks following completion of chemo (before R07198457 dose #9)	Every 3 Months After Resection Years 1-2	Every 6 Months Years 3-5, Then Annually
ELISPOT				X[7]			X[7]		X[7]		
PBMC-M	X										
Research Blood Draw[4]	X		X		X			X		X	X[5]
Vital sign collection	X		X		X	X		X		X	
Survival Status by telephone										X	X
TSH, free T3 (or total T3), free T4	X[8]				Vaccine day 22 (4th dose) only						
Amylase, lipase, LDH	X										
C-Reactive protein (CRP)	X										
Serum ferritin	X										
Urinalysis	X										
Serology: HBsAg, HBcAg, HBV DNA[12], HCV, HCV RNA[13]	X										

ECOG, Eastern Cooperative Oncology Group (See appendix 1); CA 19-9, cancer antigen 19-9; αPD-L1, anti-programmed death-ligand; CMP, Comprehensive metabolic panel

[1]Within 48 hours prior to each vaccine dose
[2]Cycles 1-12 of mFOLFIRINOX
[3]Imaging of chest/abdomen/pelvis with contrast-enhanced CT scan or MRI
[4]Research blood draws will be done prior to treatment and according to schedule as detailed in Appendix 5: Biomarker Management Plan (BMP) for imCORE ISRs
[5]Blood tests after year 2 may be done at the discretion of the treating investigator, to be collected at the time of imaging.
[6]cfDNA collected only Day 1 Cycle 1 prior to treatment
[7]If missed or unable to be analyzed, blood can be collected at the next potential clinic visit at the physician's discretion provided it does not exceed prespecified volume collections. At the physician's discretion, blood may also be collected ad-hoc for patients not following planned study procedures (e.g. early treatment discontinuation).
[8]Within 14 days of surgery
[9]Within 7 days of αPD-L1
[10]Pregnany tests will be performed on female subjects of child-bearing potential
[11]Height will only be assessed at initial visit; weight will be assessed at every MD visit
[12]HBV DNA only required for patients with positive serology for total hepatitis B core antibody (anti-HBc)
[13]HCV RNA only required for patients with positive serology for HCV
[14]Review of History and Targeted Physical Exam will be conducted by treating physicians or licensed independent practitioners (LIPs) prior to RO7198457 dose 1, 2, 6, and 9. Clinical Trial Nurses (CTN) or appropriate designees will see patient prior to RO7198457 dose 3, 4, 5, 7, and 8.

11.0 TOXICITIES/SIDE EFFECTS

All toxicities will be rated per the NCI Common Toxicity Criteria (version 5.0).

A number of measures will be taken to ensure the safety of subjects participating in this trial. These measures are addressed through exclusion criteria (see Section 6.2 Subject Exclusion Criteria) and routine monitoring as follows:

Subjects enrolled in this study will be evaluated clinically and with standard laboratory tests before and at regular intervals during their participation in this study. Safety evaluations will consist of medical interviews, recording of AEs, physical examinations, blood pressure, and laboratory measurements. Subjects will be evaluated for AEs (all grades), SAEs, and AEs requiring study drug interruption or discontinuation at each study visit for the duration of their participation in this study. Safety will be assessed through summaries of drug-limiting toxicities, AEs, changes in laboratory test results, changes in vital signs and ECGs, and exposure to study treatment. All subjects who receive any amount of study treatment (atezolizumab or RO7198457) will be included in the safety analyses.

AEs leading to treatment discontinuation will be listed. Subjects who withdraw from the study prior to completing the drug-limiting toxicity assessment window for reasons other than a drug-limiting toxicity will be considered non-evaluable for drug-limiting toxicity assessments.

Relevant laboratory, vital signs, and ECG data will be displayed by time, with NCI CTCAE Grade 3 and Grade 4 values identified, where appropriate.
Subjects who permanently discontinue RO7198457 will return to the clinic for a treatment discontinuation visit within 30 days after the last dose of study treatment. Further monitoring and recording of AEs will occur for up to 90 days after the last dose of study treatment or until initiation of another systemic anti-cancer therapy, whichever occurs first. All subjects in the study will be followed for survival and subsequent anti-cancer therapy information approximately every 3 months until death, loss to follow-up, or study termination, unless the subject requests to be withdrawn from follow-up.

11.1 Toxicity Related to mFOLFIRINOX

In a Phase 3 trial comparing mFOLFIRINOX to gemcitabine monotherapy in 493 patients with resected pancreatic cancer[79], 247 patients received mFOLFIRINOX (oxaliplatin 85 mg per square meter of BSA, irinotecan 150 mg per square meter, leucovorin 400 mg per square meter, fluorouracil 2400 mg per square meter) every 2 weeks for 24 weeks. The median number of cycles was 12, the median duration of treatment was 24.6 weeks, 66% of patients received all planned cycles. 75.9% of patients had grade 3 or higher adverse events (compared to 52.9% with gemcitabine alone), with no treatment related deaths.

The most common AEs of Grade 3 or higher were neutropenia (28%), diarrhea (18%), paresthesia (12%), fatigue (11%), neuropathy (9%), nausea (5%), vomiting (5%), hyperleukocytosis (4%), anemia (3%), and febrile neutropenia (3%).

11.2 Toxicity Related to Atezolizumab

Atezolizumab has been associated with risks such as the following: IRRs and Immune-mediated hepatitis, pneumonitis, colitis, pancreatitis, diabetes mellitus, hypothyroidism, hyperthyroidism, adrenal insufficiency, hypophysitis, Guillain-Barré syndrome, myasthenic syndrome or myasthenia gravis, meningoencephalitis, myocarditis, nephritis and myositis . In addition, systemic immune activation is considered a potential risk for atezolizumab. Please refer to the atezolizumab Investigator's Brochure (IB) for the most up to date information.

Refer to Appendix 4 for Risks Associated with Atezolizumab and Guidelines for Management of Adverse Events Associated with Atezolizumab.

11.3 Potential Risks Associated with RO7198457

Please refer to the RO7198457 Investigator's Brochure (IB) for the most up to date information.

11.3.1 Systemic reactions during or after infusion of RO7198457

The majority of patients in clinical trials with RO7198457 experienced systemic reactions often described as FLS/ILI, CRS, or IRRs, which most commonly manifest as chills (rigors) and fever within hours after the infusion, and some patients also report nausea, tachycardia, dyspnea/hypoxia, hypertension, and/or hypotension (refer to the RO7198457 Investigator's Brochure). These reactions could be potentiated when RO7198457 is administered in combination with a checkpoint inhibitor such as atezolizumab. These systemic reactions are most often manageable with supportive care and typically resolve within 24 hours.

Clinical signs and symptoms can be indistinguishable between the different types of reactions, which could range from mild to severe, life-threatening, or even fatal. Therefore, administration of RO7198457 plus atezolizumab will be performed in a setting with available emergency medical facilities with access to a critical care unit and staff who are trained to monitor for and respond to medical emergencies. Patients should be monitored closely for signs and symptoms including, but not limited to, fever, chills, rigor, headache, and myalgia, or, in more severe cases, hypotension, tachycardia, dyspnea/hypoxia, chest discomfort, wheezing, angioedema, urticaria, and rash.

To minimize the risk of systemic reactions after study drug infusion, post-infusion prophylactic treatment with antipyretics and hydration with isotonic IV fluids are required with each RO7198457 infusion (see Section 5.6 for details). Mild-to-moderate systemic reactions may occur despite post-infusion prophylactic treatment as required per protocol and/or despite optional prophylactic supportive medications administered based on prior experience, which are outlined in Table 3.

Severe or life-threatening presentations of systemic reactions, which could include adverse events such as hypotension, tachycardia, dyspnea/hypoxia, chest discomfort, rash, and other organ dysfunction should be treated aggressively with supportive and resuscitative measures as indicated, including the use of high-dose corticosteroids and

IV fluids. It should be noted that the proposed mechanism of cytokine release with RO7198457 via TLR7/8 activation is distinct from that of other molecules with risk of CRS—such as T-cell engaging bispecific antibodies or chimeric antigen receptor T-cell therapy,—and neurological toxicity has not been observed in the context of systemic reactions after infusion of RO7198457.

In case of hypersensitivity, allergic reaction or suspected anaphylaxis, when clinical symptoms may include dyspnea, bronchospasm, chest tightness, urticaria, angioedema, hypotension and other cardiovascular symptoms, and (pre-)syncope, prompt assessment and treatment, according to institutional practice, are critical.

11.3.2 Immune-mediated adverse events

Clinical experience with therapeutics intended to enhance anti-tumor T-cell responses has demonstrated that development of autoimmune inflammatory conditions is a general risk. Such Immune-mediated AEs have been described for virtually all organ systems and include, but are not limited to, colitis, hepatitis, pneumonitis, endocrinopathy, ocular toxicity, pancreatic toxicity, neurologic toxicity (myasthenic syndrome/myasthenia gravis, Guillain-Barré syndrome, and meningitis), myocarditis and rash.

In melanoma patients treated with mRNA-based vaccines, cases of vitiligo were reported and may indicate an immune-mediated reaction due to cross reactivity with wild-type antigens in normal tissue. No other AEs suggestive of an immune-mediated nature were reported in trials with mRNA-based vaccines from the same platform.

Due to this potential risk of RO7198457 to induce autoimmune conditions, patients with a history of autoimmune disease (other than autoimmune thyroid disease managed with thyroid hormone replacement or vitiligo) will be excluded from this trial.

In addition to patient selection and management guidelines, the bioinformatics workflow for creating the vaccine includes a process for excluding peptides associated with a high risk of autoimmunity. The mutation discovery, prioritization, and confirmation processes are complemented by a database that provides comprehensive information about expression levels of respective wild-type genes in healthy tissues.

This information enables the development of a personalized risk mitigation strategy by removing target candidates with an unfavorable risk profile. Mutations occurring in proteins with a possible higher auto-immunity risk in critical organs are filtered out and not considered for vaccine production.

11.3.3 Lymphopenia

Given the anticipated pharmacological vaccination effect of RO7198457 leading to homing of lymphocytes to the spleen as a primary target organ, reduction in lymphocyte count is a potential risk. Patients who received RNA-based vaccines in clinical trials conducted by BioNTech experienced transient decreases in

lymphocyte and leukocyte counts, including Grade 3 events. These events were reversible within 72 hours and did not have clinically significant sequelae.

Due to this potential risk of RO7198457 to induce lymphopenia, patients with a lymphocyte count less than 500 cells/µL will be excluded from this trial and CBCs will be monitored regularly during the trial. Additionally, mFOLFIRINOX will be started 3 weeks after the last dose of RO7198457, and CBC will be monitored to ensure resolution of lymphopenia.

11.4 Potential for Overlapping Toxicities with RO7198457 and Atezolizumab

Based on nonclinical and/or clinical studies with each molecule as a single agent and experience with molecules with similar mechanisms of action, there is a potential for overlapping toxicity in patients treated with RO7198457 plus atezolizumab. Because the expected pharmacological activity of these two molecules is to increase adaptive T-cell immune responses via complementary mechanisms, the combination may be associated with heightened immune-mediated toxicity relative to either agent alone.

Based on clinical experience to date (see the RO7198457 Investigator's Brochure), it is anticipated that any potential immune-mediated adverse events following treatment with RO7198457 plus atezolizumab will likewise be amenable to monitoring and manageable in the setting of this combination study. The extensive experience with immune checkpoint inhibitors to date was incorporated into the design and safety management plan (see Section 5.1), with the goal of reducing the risks to participating patients. Patients who were treated previously with approved or experimental cancer immunotherapy (CIT) will be excluded from this study.

11.5 Management of Patients Who Experience Specific Adverse Events Associated with RO7198457

11.5.1 Dose modifications with RO7198457

Dose modification guidance is detailed in Tables 5-7 below and Appendix 6 for dose modification administration and volume guidance.

Table 5. Dose Modification Guidance for Flu-Like Symptoms/Influenza-Like-Illness with RO7198457

CTCAE v5.0	Guidance		
Grade 2-3	• Does patient have any significant comorbidities?<br>• Was adverse event complicated by other events (e.g., Grade ≥ 3 hypotension,,dyspnea, hypoxia, azotemia, rash, urticaria, or angioedema)?<br>• Did event require hospitalization?<br>• Was time to resolution of hypotension or hypoxia ≥ 4 hours?<br>• Did associated hypotension or hypoxia occur outside the health care setting (i.e., after discharged to home from infusion area)?	"No" to all	• Current dose level of RO7198457 may be maintained in subsequent infusions.
		"Yes" to any	• RO1798457 dose level will be reduced from 25 µg to 15 µg in case of poor tolerability (e.g. prolonged or recurring rigors and chills despite supportive care with meperidine, including prophylactic meperidine) or need for corticosteroids<br>• If Grade 2 or Grade 3 AE reoccurs at the reduced dose level, a second dose reduction may occur, in consultation with the PI/Co-PI.

CTCAE v5.0 = Common Terminology Criteria for Adverse Events, Version 5.0.

Table 6. Dose Modification Guidance for Infusion-Related Reactions with RO7198457

<table>
<tr><th>CTCAE v5.0</th><th colspan="3">Guidance</th></tr>
<tr><td rowspan="2">Grade 2</td><td rowspan="2">Does patient have any significant comorbidities?
Was infusion-related reaction complicated by other events (e.g., Grade ≥ 3 dyspnea, hypoxia, azotemia, rash, urticaria, or angioedema)?
Did event require hospitalization?
Was time to resolution of hypotension or hypoxia ≥ 4 hours?
Did hypotension or hypoxia occur outside the health care setting (i.e., after discharged to home from infusion area)?</td><td>"No" to all</td><td>Current dose level of RO7198457 may be maintained in subsequent infusions.</td></tr>
<tr><td>"Yes" to any</td><td>RO1798457 dose level will be reduced from 25 µg to 15 µg in case of poor tolerability (e.g. prolonged or recurring rigors and chills despite supportive care with meperidine, including prophylactic meperidine) or need for corticosteroids
If Grade 2 infusion-related reaction reoccurs at the reduced dose level, a second dose reduction may occur, in consultation with the Principal Investigator.</td></tr>
<tr><td>Grade 3</td><td colspan="3">Continued dosing must be approved by the Principal Investigator in consultation with the treating investigator.
The dose of RO7198457 will be reduced by at least one dose level (e.g., 25 µg reduced to 15 µg) in consultation with the Principal Investigator.
In case Grade 3 infusion-related reaction reoccurs at the reduced dose level, a second dose reduction will not be allowed and RO7198457 will be permanently discontinued.</td></tr>
<tr><td>Grade 4</td><td colspan="3">No dose reductions are allowed
RO7198457 will be permanently discontinued</td></tr>
</table>

Table 7. Dose Modification Guidance for Cytokine Release Syndrome with RO7198457

CTCAE v5.0	Guidance		
Grade 1	• Does patient have any significant comorbidities?<br>• Was CRS complicated by other events (e.g., Grade ≥ 3 dyspnea, hypoxia, azotemia, rash, urticaria, or angioedema)?<br>• Did event require hospitalization?<br>• Was time to resolution of hypotension or hypoxia ≥4 hours?<br>• Did hypotension or hypoxia occur outside the health care setting (i.e., after discharged to home from infusion area)?	"No" to all	• Current dose level of RO7198457 may be maintained in subsequent infusions.
		"Yes" to any	• RO1798457 dose level will be reduced from 25 µg to 15 µg in case of poor tolerability (e.g. prolonged or recurring rigors and chills despite supportive care with meperidine, including prophylactic meperidine) or need for corticosteroids<br>• If Grade 2 infusion-related reaction reoccurs at the reduced dose level, a second dose reduction may occur, in consultation with the Principal Investigator
Grade 2	• Continued dosing must be approved by the Principal Investigator in consultation with the treating investigator.<br>• The dose of RO7198457 will be reduced by at least one dose level (e.g., 25 µg reduced to 15 µg) in consultation with the Principal Investigator.<br>• In case Grade 2 cytokine release syndrome reoccurs at the reduced dose level, a second dose reduction will not be allowed and RO7198457 will be permanently discontinued.		
Grade 3-4	• No dose reductions are allowed<br>• RO7198457 will be permanently discontinued		

11.5.2 Treatment interruption **with RO7198457**

Patients may temporarily suspend study treatment as appropriate for management of toxicity. Based on the available characterization of mechanism of action, RO7198457 in combination with atezolizumab may cause AEs similar to but independent of atezolizumab, may exacerbate the frequency or severity of atezolizumab-related AEs, or may have non-overlapping toxicities with atezolizumab. Because these scenarios cannot be distinguished from one another in the clinical setting, Immune-mediated toxicities should generally be attributed to both agents, and dose interruptions or treatment discontinuation in response to Immune-mediated AEs should be applied to RO7198457 and atezolizumab. If a planned administration of RO7198457 is delayed for ≥ 42 days for management of toxicity, then the patient will discontinue study treatment and will be followed for safety and efficacy. If, in the judgment of the investigator, the subject is likely to derive clinical benefit from resuming RO7198457 after a hold ≥ 42 days beyond when the next dose would have been given, study treatment may be restarted with the approval of the DMT Safety Committee. For example, if subjects must be

tapered off steroids used to treat Immune-mediated AEs, study treatment may be held for ≥ 42 days beyond when the next dose of RO7198457 would have been given. The acceptable length of interruption will depend on agreement between the investigator and the DMT Safety Committee.

11.5.3 Management guidelines for specific adverse events **with RO7198457**

To minimize the risk of study-emergent autoimmune inflammation, most patients with prior diagnoses of autoimmune disease will be excluded from study participation. Examples of excluded pre-existing autoimmune diseases are listed in Section 6.2 Subject Exclusion Criteria. Autoimmune thyroid disease that is managed with a stable dose of thyroid hormone replacement, type I diabetes mellitus on a stable regimen, and vitiligo are not exclusionary (see Section 6.2 Subject Exclusion Criteria). During the study, patients will be closely monitored for the development of any signs or symptoms of autoimmune conditions.

Autoimmunity is a potential risk associated with RO7198457, yet the risk is assumed to be similar to other immunotherapy agents already approved or being tested in clinical trials. Risks of autoimmunity with atezolizumab are summarized in Section 11.2 Toxicity Related to Atezolizumab and described in further detail in the Atezolizumab Investigator's Brochure. Risks of autoimmunity associated with the combination of RO7198457 and atezolizumab are unknown. Should such events occur, please refer to the Guidelines for Management of Adverse Events Associated with Atezolizumab in Appendix 4.

Events associated or possibly associated with RO7198457 or atezolizumab should be managed according to standard medical practice (e.g., thyroid hormone replacement for autoimmune hypothyroidism). Additional tests, such as autoimmune serology or biopsies, should be used to determine a possible immunogenic etiology. Although most Immune-mediated AEs observed with immune modulatory agents have been mild and self-limiting, such events should be recognized early and treated promptly to avoid potential major complications.[74,75]

Table 8. Guidelines for Management of Infusion-Related Reactions Related to Atezolizumab

CTCAE v5.0	Management
**Grade 1**	• Reduce infusion rate to half the rate being given at the time of event onset.<br>• After the event has resolved, the investigator should wait for 30 minutes while delivering the infusion at the reduced rate.<br>• If the infusion is tolerated at the reduced rate for 30 minutes after symptoms have resolved, the infusion rate may be increased to the original rate.
**Grade 2**	• Interrupt atezolizumab infusion.<br>• Administer aggressive symptomatic treatment (e.g., oral or IV antihistamine, anti-pyretic medication, glucocorticoids, epinephrine, bronchodilators, oxygen, IV fluids).<br>• After symptoms have resolved to baseline, resume infusion at half the rate being given at the time of event onset.
**Grade 3 or 4**	• Stop infusion.<br>• Administer aggressive symptomatic treatment (e.g., oral or IV antihistamine, anti-pyretic medication, glucocorticoids, epinephrine, bronchodilators, oxygen, IV fluids).<br>• Permanently discontinue atezolizumab and contact Principal Investigator.

Table 9. Guidelines for Management of Flu-Like Symptoms/influenza-Like Illness related to RO7198457

CTCAE v5.0	Guidance
**Grade 1-3**	• Treat symptomatically as indicated, including antipyretics, antihistamines, and/or analgesics as needed<br>• Treat fever and neutropenia if present<br>• Monitor fluid balance; administer IV fluids as clinically indicated<br>• Observe patients as specified in Table 3<br>• For subsequent dosing and dose reduction, please refer to Section 11.5.1

Table 10. Guidelines for Management of Infusion-Related Reactions Related to RO7198457

CTCAE v5.0	Guidance
**Grade 1-2**	• Treat symptomatically as indicated, including antipyretics, antihistamines, and/or analgesics as needed<br>• Treat fever and neutropenia if present<br>• Monitor fluid balance; administer IV fluids as clinically indicated<br>• Observe patients as specified in Table 3<br>• For subsequent dosing and dose reduction, please refer to Section 11.5.1
**Grade 3**	• Withhold further treatment with RO7198457<br>• Strongly consider cardiopulmonary and organ function monitoring in Intensive Care Unit<br>• Closely monitor and maintain fluid balance; administer IV fluids as clinically indicated<br>• Oxygen for hypoxia<br>• Vasopressor support for hypotension refractory to IV fluids<br>• Other supportive care as clinically indicated (e.g., fever and neutropenia, infection)<br>• Consider administration of corticosteroids (e.g., methylprednisolone or dexamethasone), in addition to antihistamines, antipyretics<br>• May receive the next dose of RO7198457 if symptoms resolve to Grade ≤1 for 3 consecutive days with approval of Principal Investigator<br>• For subsequent dosing and dose reduction, please refer to Section 11.5.1
**Grade 4**	• Cardiopulmonary and organ function monitoring in Intensive Care Unit<br>• Aggressive supportive treatment as described for Grade 3 IRRs (e.g., monitor/maintain fluid balance, treatment of fever and neutropenia)<br>• Mechanical ventilator support for respiratory failure<br>• Aggressive vasopressor support for hypotension<br>• Other supportive care as clinically indicated (e.g., fever and neutropenia, infection)<br>• Administer corticosteroids (e.g., methylprednisolone or dexamethasone), in addition to antihistamines, antipyretics, and/or analgesics; consider other immunosuppressive agents<br>• Permanently discontinue RO7198457

Table 11. Guidelines for Management of Cytokine-Release Syndrome Related to RO7198457

CTCAE v5.0[a]	Guidance[b]
**Grade 1**	• Treat symptomatically as indicated, including antipyretics, antihistamines, and/or analgesics as needed<br>• Treat fever and neutropenia if present<br>• Monitor fluid balance; administer IV fluids as clinically indicated<br>• Observe patients as specified in Table 3<br>• For subsequent dosing and dose reduction, please refer to Section 11.5.1
**Grade 2-3**	• Withhold further treatment with RO7198457<br>• Strongly consider cardiopulmonary and organ function monitoring in Intensive Care Unit<br>• Closely monitor and maintain fluid balance; administer IV fluids as clinically indicated<br>• Oxygen for hypoxia<br>• Vasopressor support for hypotension refractory to IV fluids<br>• Other supportive care as clinically indicated (e.g., fever and neutropenia, infection)<br>• Consider administration of corticosteroids (e.g., methylprednisolone or dexamethasone) and tocilizumab,[c] in addition to antihistamines, antipyretics<br>• May receive the next dose of RO7198457 if symptoms resolve to<br>• If Grade 2 CRS, patient may receive the next dose of RO7198457 if symptoms resolve to Grade ≤1 for 3 consecutive days with approval of Principal Investigator<br>• For subsequent dosing and dose reduction, please refer to Section 11.5.1<br>• If grade 3 CRS, permanently discontinue RO7198457
**Grade 4**	• Cardiopulmonary and organ function monitoring in Intensive Care Unit<br>• Aggressive supportive treatment as described for Grade 3 CRS (e.g., monitor/maintain fluid balance, treatment of fever and neutropenia)<br>• Mechanical ventilator support for respiratory failure<br>• Aggressive vasopressor support for hypotension<br>• Other supportive care as clinically indicated (e.g., fever and neutropenia, infection)<br>• Administer corticosteroids (e.g., methylprednisolone or dexamethasone) and tocilizumab[c], in addition to antihistamines, antipyretics, and/or analgesics; consider other immunosuppressive agents<br>• Permanently discontinue RO7198457

[a] Cytokine-release syndrome is a disorder characterized by fever, tachypnea, headache, tachycardia, hypotension, rash, and/or hypoxia caused by the release of cytokines (NCI CTCAE v5.0).
[b] Guidance for cytokine-release syndrome management adopted from Lee et al. 2014.
[c] Use of tocilizumab may be considered for the treatment of severe CRS as clinically indicated. Consider administering tocilizumab 8 mg/kg IV as a 60-minute infusion. Consider up to 3 repeat doses if no clinical improvement. Interval between doses should be at least 8 hours and dose should not exceed 800 mg per infusion. (based on the clinical experience of tocilizumab for the treatment of chimeric antigen receptor T-cell–induced severe or life-threatening CRS).

Hemophagocytic lymphohistiocytosis and Macrophage Activation Syndrome
Immune-mediated reactions may involve any organ system and may lead to hemophagocytic lymphohistiocytosis (HLH) and macrophage activation syndrome (MAS). While severe CRS and MAS/HLH have overlapping presentation and symptoms, MAS/HLH may be precipitated by other conditions including infections, autoimmune disease and malignancies (Ramos-Casals 2014).

Patients with suspected HLH should be diagnosed according to published criteria by McClain and Eckstein (2014). A patient should be classified as having HLH if five of the following eight criteria are met:

- Fever ≥ 38.5°C
- Peripheral blood cytopenia consisting of at least two of the following:
  - Hemoglobin < 90 g/L (9 g/dL) (< 100 g/L [10 g/dL] for infants < 4 weeks old)
  - Platelet count < 100 × 109/L (100,000/μL)
  - ANC < 1.0 × 109/L (1000/μL)
- Fasting triglycerides > 2.992 mmol/L (265 mg/dL) and/or fibrinogen < 1.5 g/L (150 mg/dL)
- Hemophagocytosis in bone marrow, spleen, lymph node, or liver
- Low or absent natural killer cell activity
- Ferritin > 500 mg/L (500 ng/mL)
- Soluble interleukin 2 (IL-2) receptor (soluble CD25) elevated ≥ 2 standard deviations above age-adjusted laboratory-specific norms

In all cases of suspected MAS/HLH, the Medical Monitor should be immediately notified.

Patients should be hospitalized with the following diagnostic and monitoring measures initiated:

- Frequent (e.g., every 4 hours) vital signs and physical examination including evaluation for splenomegaly;
- Serial (at least daily) monitoring of serum chemistries, complete blood counts, liver function tests (LFTs), ferritin, PT/PTT, fibrinogen, D-dimer and triglycerides;
- Consideration of bone marrow and/or lymph node biopsy to assess for hemophagocytosis and active infection, including assessment of EBV protein localization in T/B/NK cells;

- Complete infectious disease work-up including:
  - Blood cultures (bacterial and fungal)
  - Urine cultures and urinalysis
  - Radiographic assessments (e.g., chest X-ray or CT scan)
  - Assessment for active viral infections, including but not limited to EBV and CMV
- If available, assessment for soluble CD25 and assessment of NK cell function
- If available, DNA for exploratory genetic testing of mutations potentially associated with HLH (e.g., PRF1, MUNC13-4, STXBP2) should be considered (Zhang et al. 2011)

Patients with suspected MAS should be diagnosed according to published criteria for systemic juvenile idiopathic arthritis by Ravelli et al. (2016). A febrile patient should be classified as having MAS if the following criteria are met:

- Ferritin $>$ 684 mg/L (684 ng/mL)
- At least two of the following:
  - Platelet count $\leq$ 181 $\times$ 109/L (181,000/μL)
  - AST $\geq$ 48 U/L
  - Triglycerides $>$ 1.761 mmol/L (156 mg/dL)
  - Fibrinogen $\leq$ 3.6 g/L (360 mg/dL)

Patients with suspected HLH or MAS should be treated according to the guidelines in Table 17.

Table 17 Management Guidelines for Suspected Hemophagocytic Lymphohistiocytosis or Macrophage Activation Syndrome

Event	Management
Suspected HLH or MAS	• Permanently discontinue atezolizumab and contact Medical Monitor.<br>• Consider patient referral to hematologist.<br>• Initiate supportive care, including intensive care monitoring if indicated per institutional guidelines.<br>• Consider initiation of IV corticosteroids and/or an immunosuppressive agent.<br>• If event does not improve within 48 hours after initiating corticosteroids, consider adding an immunosuppressive agent.<br>• If event resolves to Grade 1 or better, taper corticosteroids over ≥ 1 month.

HLH = hemophagocytic lymphohistiocytosis; MAS = macrophage activation syndrome.

11.5.4 Discontinuation criteria

RO7198457 or atezolizumab may not have an immediate therapeutic effect, and there is no available antidote for either of these experimental agents.

The primary approach to mild-to-moderate Immune-mediated AEs (Grades 1-2) is supportive and symptomatic care. In severe cases, Immune-mediated AEs may be acutely managed with systemic corticosteroids, mycophenolate, or TNF-α antagonists[75], and either interrupting or permanently discontinuing therapy may be appropriate. Persistent or recurrent Grade 2 Immune-mediated AEs may also mandate interruption of RO7198457 or may mandate the use of steroids.

If, with continued treatment, the event should be seen to worsen such that the benefit/risk balance for a given patient is deemed unfavorable, RO7198457 should be permanently discontinued. RO7198457 must be permanently discontinued in patients with life-threatening Immune-mediated AEs or life-threatening systemic reactions during or after infusion (e.g. IRR, CRS).

11.5.5 Infusion-related reactions and guidance on premedication

Infusion-related reactions have been described with therapies administered by IV infusion, including monoclonal antibodies (mAbs). Infusion-related reactions are an identified risk of atezolizumab. Systemic reactions during or after infusion (e.g. IRR, CRS, FLS) are a potential risk with RO7198457.

Prophylactic treatment with antipyretics (e.g., acetaminophen 650-1000 mg or per institutional standard) will be required within approximately 1 hour following all RO7198457 infusions. Detailed instructions for infusion of

RO7198457, including prophylactic medication and other measures required or recommended, are included in Table 3. In addition, the investigator might decide that a patient should receive one additional post-treatment prophylactic dose of antipyretics (4-6 hours post) e.g. if a more severe reaction had been observed at the previous vaccination.

11.6 Definition of Drug-Limiting Toxicity of RO7198457 and Atezolizumab

The drug-limiting toxicity assessment window will begin following receipt of atezolizumab until 30 days following completion of following completion of RO7198457 administration. Any one of the following events will be considered a drug-limiting toxicity if it occurs during the drug-limiting toxicity assessment window and is assessed by the investigator to be related to study treatment:

- Grade ≥ 3 non-hematologic, non-hepatic AE, with the following exceptions:
  - Grade 3 fever (defined as > 40°C [104°F]) that resolves to Grade ≤ 2 in ≤ 3 days
  - Grade 3 nausea, vomiting, or diarrhea that resolves to Grade ≤ 2 with standard-of-care therapy in ≤ 3 days and does not require total parenteral nutrition or hospitalization
  - Grade 3 fatigue that resolves to Grade ≤ 2 in ≤ 7 days
  - Grade 3 AE of tumor flare (defined as local pain, irritation, or rash localized at sites of known or suspected tumor) that resolves to Grade ≤ 2 in ≤ 7 days
  - Grade 3 laboratory abnormalities that are asymptomatic and considered by the DMT Safety Committee not to be clinically significant
  - Grade 3 rash that resolves to Grade ≤ 2 in ≤ 7 days with therapy equivalent to prednisone 10 mg/day or less
  - Grade 3 arthralgia that can be adequately managed with supportive care or that resolves to Grade ≤ 2 within 7 days
  - Grade 3 autoimmune thyroiditis or other endocrine abnormality that can be managed by endocrine therapy that would not necessitate initiation of systemic corticosteroids (with the exception of replacement steroids for adrenal insufficiency)
  - Grade 3 flu-like symptoms lasting ≤ 3 days
- Grade ≥ 4 lymphopenia (absolute lymphocyte count [ALC] < 200/μL) lasting > 7 days
- Grade ≥ 4 neutropenia (ANC < 500/μL) lasting > 7 days
- Grade ≥ 3 Cytokine release syndrome that dose not resolve to Grade 1 within 5 days
- Grade ≥ 2 allergic reaction
- Grade ≥ 3 febrile neutropenia
- Grade ≥ 4 anemia
- Grade ≥ 4 thrombocytopenia or Grade 3 thrombocytopenia associated with clinically significant bleeding
- Grade ≥ 3 elevation of serum hepatic transaminase (ALT or AST) lasting > 7 days with the following exceptions:
  - Grade ≥ 3 ALT or AST elevation that is < 3 × baseline in patients with Grade 1 ALT or AST elevation at baseline as a result of liver metastases
  - Grade 3 elevation of ALT or AST lasting < 3 days in the context of Grade ≥ 2 cytokine-release syndrome
- Grade ≥ 3 elevation of serum total bilirubin

- ALT or AST > 3 × ULN in combination with total bilirubin > 2 × ULN or clinical jaundice in the absence of cholestasis or other causes of hyperbilirubinemia

  The drug-limiting toxicity assessment window will begin following receipt of atezolizumab until 30 days following completion of Cycle 12 of mFOLFIRINOX.

11.7 Treatment Discontinuation Criteria

Discontinuation criteria apply for all drug-related AEs attributed to mFOLFIRINOX, atezolizumab, or tumor vaccine, or any combination thereof.

Treatment should be discontinued for the following:

- Pregnancy
- Any Grade 2 drug-related uveitis, eye pain, or blurred vision that does not respond to topical therapy and does not improve to Grade 1 severity within the re-treatment period, or that requires systemic treatment.
- Any Grade 3 non-skin, drug-related AE lasting > 7 days, with the following exceptions:
  - Grade 3 drug-related uveitis, pneumonitis, bronchospasm, diarrhea, colitis, neurologic toxicity, hypersensitivity reaction, or infusion reaction of any duration requires discontinuation
  - Grade 3 drug-related laboratory abnormalities do not require treatment discontinuation except:
    - Grade 3 drug-related thrombocytopenia > 7 days or associated with bleeding requires discontinuation
    - Any drug-related liver function test (LFT) abnormality that meets the following criteria requires discontinuation:
      - AST or ALT > 8 x ULN or
      - Total bilirubin > 5 x ULN or
      - Concurrent AST or ALT > 3 x ULN and total bilirubin > 2 x ULN
- Any Grade 4 drug-related AE or laboratory abnormality, except for the following events which do not require discontinuation:
  - Isolated Grade 4 amylase or lipase abnormalities that are not associated with symptoms or clinical manifestations of pancreatitis and decrease to < Grade 4 within 1 week of onset
  - Isolated Grade 4 electrolyte imbalances/abnormalities that are not associated with clinical sequelae and are corrected with supplementation/appropriate management within 72 hours of their onset
- Any dosing interruption lasting > 6 weeks with the following exceptions:
  - Dosing interruptions to allow for prolonged steroid tapers to manage drug-related AEs are allowed for up to 12 weeks. Prior to re-initiating treatment in a subject with a dosing interruption lasting > 6 weeks, the Principal Investigator must be consulted. Tumor assessments should continue as per protocol even if dosing is interrupted
  - Dosing interruptions > 6 weeks that occur for non-drug-related reasons may be allowed if approved by the Principal Investigator. Prior to re-initiating treatment in a subject with a dosing interruption lasting > 6 weeks, the Principal Investigator must be consulted

Tumor assessments should continue as per protocol even if dosing is interrupted. If treatment is discontinued due to any of the above, with the exception of pregnancy, it will be considered a DLT.

11.8 Study Treatment Discontinuation

Subjects must discontinue RO7198457 and atezolizumab treatment if they experience any of the following:

- Symptomatic deterioration (e.g., uncontrollable pain secondary to disease, unmanageable ascites) attributed to disease progression as determined by the investigator after an integrated assessment of all the radiographic data, biopsy results, and clinical status
- Intolerable toxicity related to RO7198457 and atezolizumab treatment, including development of an Immune-mediated AE determined by the investigator to be unacceptable given the individual subject's potential response to therapy and the severity of the event
- Any medical condition that the investigator and/or DMT Safety Committee determines may jeopardize the subject's safety if he or she continues on study treatment

- Use of a non-protocol systemic anti-cancer therapy
- Pregnant subjects will be withdrawn from study treatment
- In addition, the investigator has the right to withdraw a subject from study treatment at any time. Reasons for withdrawal from study treatment may include, but are not limited to, the following:
  - Investigator determines it is in the best interest of the subject
  - Non-compliance

- The primary reason for study treatment discontinuation must be documented. Subjects who discontinue study treatment primarily for reasons other than disease progression will continue tumor assessments, and all subjects who discontinue study treatment will continue to be followed for survival every 3 months in years 1-2 after surgery, every 6 months in years 3-5, and then once a year unless consent is withdrawn
- Subjects who discontinue chemotherapy if deemed medically appropriate by the investigator will received the final dose of the tumor vaccine approximately 3 Weeks following completion of chemo (+/- 2 weeks), and will continue to be followed for survival every 3 months in years 1-2 after surgery, every 6 months in years 3-5, and then once a year unless consent is withdrawn.

11.8.1 Study treatment discontinuation visit

Subjects who discontinue study treatment will be asked to return to the clinic for a treatment discontinuation visit at ≤ 30 days after the last administration of study treatment.

The visit at which a response assessment shows disease recurrence, which results in discontinuation of RO7198457 and atezolizumab, may be used as the treatment discontinuation visit as applicable, in which case, all assessments associated with the treatment discontinuation visit should be performed at that time.

11.8.2 Survival and subsequent anti-cancer therapy follow-up

Following study treatment discontinuation, all subjects will be followed for survival and subsequent anti-cancer therapy. Survival and subsequent anti-cancer therapy follow-up information will be collected via telephone calls, patient medical records, and/or clinic visits approximately every 6 months until death, loss to follow-up, or study termination unless the subject requests to be withdrawn from follow-up.

Information on subsequent anti-cancer therapies will include systemic therapies (e.g., chemotherapy, targeted therapy, hormonal therapy, or immunotherapy), surgery (e.g., resection of metastatic disease), and radiation procedures (e.g., radiotherapy to a tumor lesion).

If the subject withdraws from the study, the site's staff may use a public information source (e.g., county records) to obtain information about survival status only.

11.9 mFOLFIRINOX Dose Modification Schedule

Subjects should be assessed for toxicity before each dose. Dose modification should be performed according to the schedule of toxicity assessments as outlined in Table 4 in Section 10.0 EVALUATION DURING TREATMENT/INTERVENTION. Subjects with ongoing Clavien-Dindo Grade 1-2 postoperative complications (Section 20.0 APPENDICES, Appendix 2) who, according to the DMT Safety Committee, require delay of initiation of adjuvant therapy, may delay atezolizumab up to 3 weeks and RO7198457 doses up to 2 weeks to allow recovery. All atezolizumab and RO7198457 -related toxicities will be treated using dose interruptions. All non-hematologic chemotherapy-related toxicities will be treated using dose reductions detailed in the dose level table below. Chemotherapy-related toxicities will be treated with dose interruptions detailed in Tables 12-

mFOLFIRINOX Dose Levels:

Dose Level	5-FU infusion (mg/m^2)	Leucovorin (mg/m^2)	Irinotecan (mg/m^2)	Oxaliplatin (mg/m^2)
0*	2400	400	150	85
-1	1920	400	120	65
-2	1600	400	90	50
-3	1360	400	50	40

*Dose level 0 refers to the starting dose.

*If clinically indicated, dose adjustment in one or more drugs by more than 1 level is permissible at the discretion of the PI and/or treating physician. Details and rationale must be supported in the medical record.

Subjects who are 76-years-of age or older may receive dose-adjusted mFOLFIRINOX starting at DL -1 (recommended, but not mandatory) at the discretion of the PI and/or treating physician (dose levels table below). DL 0 is the recommended standard starting dose level for patients aged <=75 years.

Table 12: Dose Modifications for Neutropenia and/or Thrombocytopenia at the Start of a Cycle or Within a Cycle

**Toxicity and Grade**	**Suggested Dose Modification**				
	**Occurrence**	**5-Fluorouracil**	**Irinotecan**	**Oxaliplatin**	**Leucovorin**
Neutropenia* Grade 3/4<br><br>Hold treatment and check weekly until ANC ≥ 1500/mm^3<br><br>Either GCSF or Peg-GCSF is permitted per institutional/national guidelines.<br>1. Prophylactic use of G-CSF is permitted for high risk patients.	First	Maintain the dose	Reduce to DL -1	Maintain the dose	Maintain the dose
	Second	Reduce to DL -1	Maintain the dose	Maintain the dose	Reduce to DL-1
	Third	Maintain the dose	Maintain the dose	Reduce to DL -1	Maintain the dose
	Fourth	Discuss with PI/Co-PI			
Thrombocytopenia Grade 2 (1st Event)	Hold treatment and check weekly until platelets are ≥ 75,000/mm^3. Resume treatment at same dose level.				
Thrombocytopenia Grade 2 (2nd or subsequent Event) or Grade 3/4<br><br>Hold treatment and check weekly until platelets are ≥ 75,000/mm^3.	First	Maintain the dose	Maintain the dose	Reduce to DL -1	Maintain the dose
	Second	Maintain the dose	Reduce to DL-1	Maintain the dose	Maintain the dose
	Third	Maintain the dose	Maintain the dose	Reduce to DL -2	Maintain the dose
	Fourth	Discuss with PI/Co-PI			
*If a participant had febrile neutropenia, the ANC must have resolved to ≥ 1500/mm^3 and the participant must have recovered from infection to permit treatment.<br><br>Other hematologic toxicities do not require dose modification. However, red blood cell transfusion should be considered for hemoglobin < 7 g/dL or significant symptoms of anemia or per institutional guidelines.					

Table 13: Dose Modifications for Diarrhea

Toxicity and Grade	Suggested Dose Modification				
	Occurrence	5-Fluorouracil	Irinotecan	Oxaliplatin	Leucovorin
Diarrhea Grade 1-2	No Dose modification; Initiate/optimize supportive care				
Diarrhea Grade 3-4<br><br>Hold treatment until resolves ≤ Grade 1<br><br>Optimize supportive care	First	Maintain the dose	Reduce to DL -1	Maintain the dose	Maintain the dose
	Second	Reduce to DL-1	Maintain the dose	Maintain the dose	Reduce to DL -1
	Third	Maintain the dose	Reduce to DL -2	Maintain the dose	Maintain the dose
	Fourth	Discuss with PI/Co-PI			
For symptoms of diarrhea (and/or abdominal cramping) that occur at any time during a treatment cycle, it is suggested that participants should be instructed to take an anti-diarrheal, such as loperamide (2 mg every 2 hours until diarrhea resolves for 12 hours; 4 mg q 4 hours at night is allowed) or diphenoxylate/atropine (Lomotil) as treatment for diarrhea.<br><br>Acute diarrhea and abdominal cramps, developing during or within 24 hours after irinotecan administration, may occur as part of a cholinergic syndrome. For irinotecan-related cholinergic reactions, the infusion time may be increased to mitigate these symptoms and prophylactic atropine per institutional guidelines is permitted.					

Dose Modifications for Drug Related Hepatic Toxicity

For all hepatobiliary toxicity, hold treatment and evaluate for non-drug causes, e.g., biliary obstruction/stent malfunction. Once underlying etiology is corrected and improving, resume therapy (5-Fluoruracil and Oxaliplatin only without irinotecan) at the previous dose level and add Irinotecan once toxicity improves to < grade 1.

Use the following dose modification guidelines for hyperbilirubinemia

1. Grade 2 and Grade 3 hyperbilirubinemia – omit irinotecan until grade <u><</u>1 and resume at the same dose level
2. Grade 4 hyperbilirubinemia – hold therapy until <u><</u>1 and resume at the next dose level once underlying etiology is corrected*

*<u>Note:</u> If the etiology of hyperbilirubinemia is from biliary obstruction (ie. reversible and non-therapy related), discussion with the PI/Co-PI may permit the option to continue irinotecan at original levels once liver function tests are resolved to < Grade 1.

For known Gilbert's Disease:

When administered in combination with other agents or as a single-agent, a reduction in the starting dose of irinotecan by at least one level should be considered for participants known to be homozygous for the UGT1A1*228 allele. However, the precise dose reduction in this patient population is not known and subsequent dose modifications should be considered based on individual participant tolerance to treatment after discussion with the PI/Co-PI.

Table 14: Dose Modifications for Mucositis

Toxicity and Grace	Suggested Dose Modification				
	Occurrence	5-Fluorouracil	Irinotecan	Oxaliplatin	Leucovorin
Mucositis Grade 3<br><br>Hold 5-FU, Oxaliplatin, and Irinotecan until recovery to grade ≤ 1	First	Reduce to DL -1	Maintain the dose	Reduce to DL -1	Reduce to DL -1
	Second	Reduce DL -2	Maintain the dose	Reduce to DL -2	Reduce to DL -2
	Third	Reduce to DL-3	Maintain the dose	Reduce to DL -3	Reduce to DL -3
	Fourth	Discuss with PI/Co-PI			
Mucositis Grade 4<br><br>Hold 5-FU, Oxaliplatin, and Irinotecan until recovery to grade ≤ 1	First	Reduce to DL -1	Reduce to DL -1	Reduce to DL -1	Reduce to DL -1
	Second	Reduce to DL -2	Reduce to DL -2	Reduce to DL -2	Reduce to DL -2
	Third	Reduce to DL -3	Reduce to DL -3	Reduce to DL -3	Reduce to DL -3
	Fourth	Discuss with PI/Co-PI			

Dose Modifications for Persistent Neuropathy

1. Grade 1 neurotoxicity – continue monitoring and same dose level
2. Grade 2 neurotoxicity - decrease oxaliplatin by one dose level and continue 5-FU, Irinotecan and Leucovorin at the same dose level
3. Grade 3 neurotoxicity - hold Oxaliplatin until recovery to < grade 1, continue 5-FU, Irinotecan and Leucovorin at same dose level, once resolved oxaliplatin can be resumed at next dose level after discussion with PI/Co-PI
4. Grade 4 neurotoxicity – discontinue oxaliplatin, continue 5-FU, Irinotecan and Leucovorin at same dose level, if resolved to < grade 1, therapy can be resumed on case by case basis after discussion with PI/Co-PI

Table 15: Dose Modifications for Hand Foot Syndrome

Toxicity and Grade	Suggested Dose Modification				
	Occurrence	5-Fluorouracil	Irinotecan	Oxaliplatin	Leucovorin
Hand Foot Syndrome Grade 3-4	First	Reduce to DL -1	Maintain the dose	Maintain the dose	Reduce to DL -1

Hold 5-FU until recovery to ≤ Grade 1, continue Irinotecan, Oxaliplatin and Leucovorin	Second	Reduce to DL -2	Maintain the dose	Reduce to DL -1	Reduce to DL -2
	Third	Reduce to DL -3	Maintain the dose	Reduce to DL -2	Reduce to DL -3
	Fourth	Discuss with PI/Co-PI			

Table 16: Dose Modifications for Other Clinically Significant Non-Hematologic* Toxicities (except alopecia and Grade 3 nausea and vomiting responding to medical treatment within 72 hours)

mFOLFIRINOX is considered a moderately emetogenic regimen. Anti-emetic therapy is as per institutional guidelines and a suggested regimen pre-treatment includes the following agents:

5. 5-HT3 antagonist
6. Dexamethasone
7. Fosaprepitant or equivalent substance P/neurokinin 1 receptor antagonist
8. Lorazepam

**Grade**	**Suggested Dose Modification**				
	**Occurrence**	**5-Fluorouracil**	**Irinotecan**	**Oxaliplatin**	**Leucovorin**
Grade 3<br><br>Hold treatment until AE resolves to ≤ Grade 1.	First	Reduce the suspected offending agent by one dose level			
	Second	Reduce the suspected offending agent by one more dose level			
	Third	Discontinue the suspected offending agent			
	Fourth	Discuss with PI/Co-PI			
Grade 4<br><br>Hold treatment until AE resolves to ≤ Grade 1.	First	Discontinue the suspected offending agent			
	Second	Discuss with PI/Co-PI			
*Determination of "clinically significant" AEs and "offending drug" is at the discretion of the treating physician and/or PI/Co-PI.					

Table 17: Dose Modifications for Infusion Reactions

Either institutional guidelines or those described below should be followed in case of infusion reactions to any study treatment given per protocol (e.g. nal-IRI, oxaliplatin etc.). Infusion reactions will be defined according to the National Cancer Institute CTCAE (version 5) definitions of an allergic reaction or anaphylaxis as noted below.

**Grade**	**Suggested Dose Modification**

Grade 1:<br>Transient flushing or rash, drug fever <38° C (<100.4°F); intervention no indicated	• Slow infusion rate of offending agent by 50%.<br>• Monitor patient every 15 minutes for worsening of condition.<br>• Future infusions may be administered at a reduced rate (e.g. over 60-120 minutes for irinotecan), at the discretion of the Investigator.<br><br>NOTE: Pre-medication with a combination of diphenhydramine hydrochloride 25-50 mg IV, dexamethasone 10- 20 mg IV, and acetaminophen 650 mg orally or per institutional guidelines may be provided as part of subsequent treatments.
Grade 2:<br>Intervention or infusion interruption indicated; responds promptly to symptomatic treatment (e.g., antihistamines, NSAIDS, narcotics); prophylactic medications indicated for ≤ 24 hours.	• Stop infusion of offending agent.<br>• Administer diphenhydramine hydrochloride 25-50 mg IV, acetaminophen 650 mg orally, and oxygen.<br>• Resume infusion at 50% of the prior rate once infusion reaction has resolved.<br>• Monitor subject every 15 minutes for worsening of condition.<br>• For all subsequent infusions, pre-medicate with diphenhydramine hydrochloride 25-50 mg IV, dexamethasone 10-20 mg IV, and acetaminophen 650 mg orally.<br>• Future infusions may be administered at a reduced rate (e.g. over 60-120 minutes for irinotecan), at the discretion of the Investigator.
Grade 3:<br>Prolonged (e.g., not rapidly responsive to symptomatic medication and/or brief interruption of infusion); recurrence of symptoms following initial improvement; hospitalization indicated for clinical sequelae (e.g., renal impairment, pulmonary infiltrates).	• Stop infusion and disconnect infusion tubing from patient.<br>• Administer diphenhydramine hydrochloride 25-50 mg IV, dexamethasone 10-20 mg IV, bronchodilators for bronchospasm, and other medications or oxygen as medically necessary.<br>• No further treatment will be permitted during this visit.<br>• Subsequent treatment is permitted after consultation with allergist; de-sensitization to oxaliplatin as per institutional guidelines with premedication with a combination of diphenhydramine hydrochloride 25-50 mg IV, dexamethasone 10-20 mg IV, monteleukast 10 mg and acetaminophen 650 mg orally
Grade 4:<br>Life-threatening consequences; urgent intervention indicated.	• Stop the infusion and disconnect infusion tubing from subject.<br>• Administer epinephrine, bronchodilators or oxygen as indicated for bronchospasm.<br>• Administer diphenhydramine hydrochloride 50 mg IV, dexamethasone 10-20 mg IV and other medications as medically necessary.<br>• Consider hospital admission for observation.<br>• No further treatment will be permitted during this visit.

11.10 Prescribed Therapy During Study Period

Subjects must be withdrawn from the study if they receive any other investigational agents, αPD-1 or αPD-L1 agents, experimental or approved anti-tumor therapies, chemotherapy, or radiotherapy (with the exception of use for pain control).

Subjects should not schedule any elective surgeries (excluding primary tumor resection at MSKCC or central venous catheter placement) during their participation in the study, or until 7 days after their last administration of study treatment. If a subject undergoes any unexpected surgery during the course of the study, that subject must discontinue all study treatment immediately, and the treating physician should be notified as soon as possible. A subject may be allowed to resume study treatment after each surgical case is reviewed by the study team and the investigator to determine the appropriateness of treatment resumption.

12.0 CRITERIA FOR THERAPEUTIC RESPONSE/OUTCOME ASSESSMENT

The primary objective of this Phase 1 study will be to assess the safety of a personalized tumor RO7198457 in combination with mFOLFIRINOX, and atezolizumab.

Secondary objectives include assessment of RFS and OS.

Exploratory scientific correlates include baseline TCR profiling and the effect of checkpoint inhibition immunotherapy on the TCR profile, and to identify blood and tissue-specific transcriptomic and immunologic changes with RO7198457 and immune checkpoint inhibition therapy. Additional scientific correlates include assessing changes in CA 19-9 serum levels throughout the course of treatment.

12.1 Toxicity Assessment

Toxicity assessments will be performed every 2 weeks, and drug-related AEs of all grades will be recorded. Descriptive statistics of safety will be presented using NCI CTCAE version 5.0. All on-study AEs, Grade 3-4 AEs, treatment-related AEs, Grade 3-4 treatment-related AEs, SAEs, treatment-related SAEs, and AEs leading to discontinuation will be tabulated using worst grade per NCI CTCAE version 5.0 criteria by system organ class and preferred term. On-study lab parameters, including hematology, chemistry, liver function, and renal function and Grade 3-4 lab abnormalities will be summarized using worst-grade NCI CTCAE version 5.0 criteria. The safety of tumor RO7198457/mFOLFIRINOX/atezolizumab will be summarized as the percentage of subjects with Grade 3 or 4 drug-related AE or treatment termination due to a drug-related AE.

The drug-limiting toxicities of combination tumor RO7198457/mFOLFIRINOX/atezolizumab will be assessed. A drug-limiting toxicity is defined as a ≥ Grade 3 drug-related AE occurring during treatment (excluding tumor flare defined as local pain, irritation, or rash localized at sites of known or suspected tumor or a transient Grade 3 infusion AE). A drug-limiting toxicity will be considered related to the study regimen unless there is a clear, well-documented alternative explanation for the AEs. See Section 11.6 (Definition of Drug-Limiting Toxicity of RO7198457 and Atezolizumab) for the full definition of drug-limiting toxicity.

12.2 Determination of Recurrence-Free Survival

For purposes of this study, subjects will undergo surveillance imaging of the chest, abdomen, and pelvis with CT or MRI 3 months after tumor resection and will be re-evaluated every 3 months during Years 1 and 2, every 6 months during Years 3-5, and then annually. Disease recurrence will be determined by the appearance of a new lesion on follow-up cross-sectional imaging. RFS will be calculated from the date of tumor resection to the date of the appearance of a new lesion on cross-sectional imaging. New lesions will be biopsied to confirm disease recurrence and for WES.

12.3 Definitions of Measurable and Non-Measurable Disease (per RECIST v.1.1)

12.3.1 Measurable disease

Measurable disease is defined as at least 1 lesion whose longest diameter can be accurately measured as ≥ 2 cm with chest X-ray or as ≥ 1 cm with CT scan, CT component of a PET/CT, or MRI. Clinical lesions will only be considered measurable when they are superficial (e.g., skin nodules, palpable lymph nodes). Lesions on chest x-ray are acceptable as measurable lesions when they are clearly defined and surrounded by aerated lung; however, CT is preferable.

12.3.2 Non-measurable disease

All lesions or sites of disease, including small lesions (longest diameters < 2 cm with chest X-ray or < 1 cm with CT scan, CT component of a PET/CT, or MRI) are considered non-measurable disease. Bone lesions, leptomeningeal disease, ascites, pleural/pericardial effusions, lymphangitis cutis/pulmonis, inflammatory breast disease, abdominal masses (not followed by CT or MRI), and cystic lesions are all non-measurable.

12.4 Guidelines for Evaluation of Measurable Disease

Acceptable imaging modalities for measurable disease: CT scan (conventional and spiral), MRI, chest X-ray, and physical examination.

- Conventional CT and MRI should be performed with cuts of 1.0 cm or less in slice thickness contiguously
- Spiral CT must be performed using a 5 mm contiguous reconstruction algorithm
- The cytological confirmation of the neoplastic origin of any effusion that appears or worsens during treatment is mandatory to differentiate between an effusion as a side effect of the treatment and recurrent disease
- Cytologic and histologic techniques can be used to confirm disease recurrence in rare cases

All identified sites of disease recurrence must be followed on re-evaluation. Specifically, a new lesion cannot be confirmed as recurrent disease without re-checking on follow-up imaging.

12.5 Determination of Overall Survival

OS will be calculated from date of surgical resection of the primary tumor to date of death of any cause. For subjects who do not die before the end of the study or who are lost to follow-up, OS will be censored at the date of last contact.

12.6 Determination of RO7198457 -Specific Immunologic Changes at Baseline and After Therapy

RO7198457 -specific immunity will be assessed by comparing the frequency of RO7198457-reactive $CD8^{+}$ T-cells in blood specimens before, during, and after vaccination using cellular immunologic assays. Modulation of RO7198457-specific immunity with chemotherapy will be assessed by comparing these readouts in pre- and post-chemotherapy blood samples. The Vinod Balachandran Laboratory, MSKCC, has extensive expertise in these assays.[33]

12.7 Identify Blood and Tissue-Specific Genomic, Transcriptomic, and Immunologic Changes with RO7198457 and Immune Checkpoint Inhibition Therapy

Neoantigen-specific immunity will be assessed by comparing the frequency of individual neoantigen-reactive T-cell clones (identified using TCR sequencing) in pre-, during-, and post-vaccination blood specimens. The Vinod Balachandran Laboratory, MSKCC, has extensive expertise in these assays.[33] Additional exploratory correlates may be performed as outlined in Section 4.3 Biomarker Assessments and Section 4.4 Tumor Tissue Specimen Assessments.

13.0 CRITERIA FOR REMOVAL FROM STUDY

1. If, at any time, recurrent disease is identified, the subject will be taken off study and referred for alternative therapy. Subjects with otherwise stable or improved performance and clinical status may receive RO7198457 and chemotherapy according to the protocol schema for 1 additional cycle. At the next scheduled imaging evaluation, if there is further increase in the sum of the longest diameter or development of additional new lesions, then the subject will stop treatment and complete off-trial treatment.
2. If, at any time, unacceptable toxicity is identified, the subject will be removed from the study. If, at any time, protocol ineligibility is identified, as designated in Section 6.0 CRITERIA FOR SUBJECT ELIGIBILITY (i.e., change in diagnosis), the subject will be removed from the study.
3. If, at any time, a subject elects to discontinue treatment.
4. Changes in a subject’s condition which render the subject unacceptable for further treatment in the judgment of the DMT Safety Committee.
5. Inability of the subject to comply with study requirements.
6. Determination by the DMT Safety Committee that it is no longer safe for the subject to continue therapy.

All subjects removed from the study during the DLT assessment window for reasons 3, 4, 5, and 6 will be replaced. The DLT assessment period begins from administration of atezolizumab until 30 days after completion of Cycle 12 (or the final cycle) of mFOLFIRINOX.

14.0 BIOSTATISTICS

The primary objective of this trial is to evaluate the safety of a personalized tumor vaccine combined with atezolizumab and mFOLFIRINOX. The primary endpoint is Grade 3 or higher drug-related AE or treatment termination due to any-grade drug-related AE. See Section 11.6 (Definition of Drug-Limiting Toxicity of RO7198457 and Atezolizumab) for the full definition of drug-limiting toxicity. Unlike most other Phase 1 trials in oncology, there is no dose escalation; instead, we will monitor toxicity through the following series of stopping rules:

Number of Patients	Number of Drug-Limiting Toxicities* Required to Stop the Trial
3-10	3 or More
11-16	4 or More
17-20	5 or More

*A drug-limiting toxicity is defined as a ≥ Grade 3 immune- or RO7198457 or drug-related AE occurring from receipt of atezolizumab until 30 days after completion of Cycle 12 or the final cycle of mFOLFIRINOX, excluding a transient Grade 3 infusion AE, using the National Cancer Institute (NCI) Common Terminology Criteria for Adverse Events (CTCAE) version 5.0. A drug-limiting toxicity will be considered related to the study regimen unless there is a clear, well-documented alternative explanation for the AEs. AEs ascribed to mFOLFIRINOX will not be considered a drug-limiting toxicity. See Section 11.6 (Definition of Drug-Limiting Toxicity of RO7198457 and Atezolizumab) for the full definition of drug-limiting toxicity.

Enrollment of the first 2 patients will be staggered by 2 weeks.

If the trial stops because these boundaries are crossed, we will conclude that the treatment is unacceptably toxic. This rule has Type I error of 9.4% and Type II error of 10.6% if the unacceptable and acceptable toxicity rates are 34% and 10%, respectively. Probability of stopping the trial is given for other configurations of toxicity rate in Table 2. Expected sample sizes confirm that this design will allow a tolerable treatment to go to full accrual while exposing less than half of the planned patients on average if the treatment is unacceptably toxic.

Probability of Toxicity	Probability of Stopping	Expected Sample Size
0.10	0.106	19.0
0.16	0.324	17.0
0.22	0.578	14.4
0.28	0.782	12.0
0.34	0.906	9.9
0.40	0.967	8.5

Secondary objectives:

- To assess RFS, defined as time from surgical resection to date of recurrence. This will be accomplished using Kaplan-Meier methods using deaths without recurrence as events.
- To assess OS, defined as time from surgical resection to date of death. This will be accomplished using Kaplan-Meier methods

Exploratory scientific correlates:

- To assess induction of tumor-specific immune responses with RO7198457 and immune checkpoint inhibition therapy: Interferon gamma (IFNg) expression will be measured at 10 time points (pre-vaccination, 8 times during vaccination, and post-vaccination). Summary statistics will be presented by each time point. Changes from pre- to post-vaccination will be tested using the signed ranks test. A longitudinal analysis method, such as generalized estimating equations, will be used to analyze the full set of data
- To evaluate cancer antigen (CA) 19-9 biomarker changes over time: The purpose of this analysis is to associate the biomarker changes with immune response (binary). At each time point where they are measured (every 3 months) we will use Kendall's tau to report the correlation between change from baseline in the biomarker value with immune response at that time point. We will also evaluate correlations between change in biomarker with the next time point's immune response to see if biomarker changes might be an early signal of immune response/failure. Similar to immune response, CA 19-9 will be classified as a continuous variable.
- To identify the most immunogenic neoantigens for clinical vaccines: Each neoantigen consists of 20 epitopes (targets). We will determine by T cell sequencing the number of T cells and number of clones specific to each target. We will rank the targets by the average number of responding T cells and average number of responding clones per patient. We will compare post hoc the immunogenicity of neoantigens for clinical vaccines as predicted by the BioNTech/Genentech pipeline and our previously published antigen selection strategy.[33]
- To evaluate patterns of failure / disease recurrence: To evaluate patterns of failure (disease recurrence), sites of metastatic disease will be recorded for each subject based on findings from surveillance cross-sectional imaging. We will group failures in four categories: liver only, lung only, liver and lung, and others. We will use RFS outcome as binary for the purposes of this analysis, dichotomized at 18 months. We will report the multinomial proportions along with 95% confidence intervals.

15.0 RESEARCH PARTICIPANT REGISTRATION AND RANDOMIZATION PROCEDURES

15.1 Research Participant Registration

Confirm eligibility as defined in the section entitled Inclusion/Exclusion Criteria. Obtain informed consent, by following procedures defined in section entitled Informed Consent Procedures. During the registration process, registering individuals will be required to complete a protocol-specific Eligibility Checklist. The individual signing the Eligibility Checklist is confirming whether or not the participant is eligible to enroll in the study. Study staff are responsible for ensuring that all institutional requirements necessary to enroll a participant to the study have been completed. See related Clinical Research Policy and Procedure #401 (Protocol Participant Registration).

16.0 DATA MANAGEMENT ISSUES

A Clinical Research Coordinator (CRC) will be assigned to the study. The responsibilities of the CRC include project compliance, data collection, abstraction and entry, data reporting, regulatory monitoring, problem resolution and prioritization, and coordination of the activities of the protocol study team.

The data collected for this study will be entered into the study database (Medidata). Source documentation will be available to support the computerized patient record. Grade 3 and 4 toxicities will be captured.

16.1 Quality Assurance

Weekly registration reports will be generated to monitor patient accruals and completeness of registration data. Routine data quality reports will be generated to assess missing data and inconsistencies. Accrual rates and extent and accuracy of evaluations and follow-up will be monitored periodically throughout the study period, and potential problems will be brought to the attention of the study team for discussion and action. Random-sample data quality and protocol compliance audits will be conducted by the study team, at a minimum of 2 times per year; more frequently if indicated.

16.2 Data and Safety Monitoring

The Data and Safety Monitoring (DSM) Plans at MSKCC were approved by the NCI in September 2001. The plans address the new policies set forth by the NCI in the document entitled "Policy of the National Cancer Institute for Data and Safety Monitoring of Clinical Trials," which can be found at http://cancertrials.nci.nih.gov/researchers/dms/index.html. The DSM Plans at MSKCC were established and are monitored by the Office of Clinical Research. The MSKCC DSM Plans can be found on the MSKCC Intranet at: https://one.mskcc.org/sites/pub/clinresearch/Documents/MSKCC%20Data%20and%20Safety%20Monitoring%20Plans.pdf.

There are several different mechanisms by which clinical trials are monitored for data, safety, and quality. There are institutional processes in place for quality assurance (QA) (e.g., protocol monitoring, compliance and data verification audits, therapeutic response, and staff education on clinical research QA) and departmental procedures for quality control, and there are 2 institutional committees that are responsible for monitoring the activities of our clinical trials programs. The committees—the Data Safety Monitoring Committee (DSMC) for Phase 1 and 2 clinical trials, and the Data and Safety Monitoring Board (DSMB) for Phase 3 clinical trials—report to the MSKCC Research Council and IRB.

During the protocol development and review process, each protocol will be assessed for its level of risk and degree of monitoring required. Every type of protocol (e.g., National Institutes of Health [NIH] sponsored, in-house sponsored, industrial sponsored, NCI cooperative group, etc.) will be addressed, and the monitoring procedures will be established at the time of protocol activation.

16.3 Regulatory Documentation

Prior to implementing this protocol at MSKCC, the protocol, informed consent form, HIPAA authorization, and any other information pertaining to participants must be approved by the MSKCC Institutional Review Board/Privacy Board (IRB/PB).

16.3.1 Amendments

Each change to the protocol document must be organized and documented by MSKCC and first approved by the MSKCC IRB/PB.

16.3.2 Additional IRB correspondence

Deviations and Violations: A protocol deviation on this study is defined as a request to treat a research participant who does not meet all eligibility criteria, pretreatment evaluation, or who requires alteration in their study plan. If a deviation from this protocol is proposed for a potential or existing participant, approved from the MSKCC IRB/PB is required prior to the action.

A protocol violation is any change or departure from the research protocol that occurred without prior approval from the MSKCC IRB/PB. The MSKCC PI will report violations to the MSKCC IRB/PB.

16.4 Document Maintenance

The MSKCC PI will maintain adequate and accurate records to enable the implementation of the protocol to be fully documented and the data to be subsequently verified.

17.0 PROTECTION OF HUMAN SUBJECTS

Participation in this trial is voluntary. All subjects will be required to a sign a statement of informed consent, which must conform to IRB guidelines. The study will protect the rights of all human subjects, and an informed consent will clearly define the risks, benefits, toxicities, and side effects of treatment. The subject will also be informed of the alternative options for treatment.

Inclusion of Women and Minorities: MSKCC has filed forms HHS 441 (Civil Rights), HHS (Handicapped Individual), 639-A (Sex Discrimination), and 680 (Age Discrimination); we also take due notice of the NIH policy concerning inclusion of women and minorities in clinical research populations. Patients of all races, both male and female, will be accepted into the protocol. The proposed study population is as described in Section 7.0 RECRUITMENT PLAN.

Exclusion of Lactating or Pregnant Women: Lactating and pregnant women are also excluded because of potential teratogenic effects of treatment that may be harmful to the developing fetus or nursing infant.

Exclusion of Children: Children have been excluded from this study. PDAC is an adult cancer. Thus, the relevance of this drug to the pediatric population has not been established.

Benefits: It is possible that this treatment will result in prevention of recurrence of PDAC. It is not known whether these or any other favorable events will occur. It is not known whether this treatment will affect the OS of the patients.

Incentives: No incentives will be offered to patients/subjects for study participation.

Alternatives: For patients with PDAC, alternative treatments may include other chemotherapy regimens as well as proceeding to surgery or research adjuvant chemotherapy. Patients may be eligible for other investigational studies.

Confidentiality: Every effort will be made to maintain patient confidentiality. Research and hospital records are confidential. Patients' names and any other personally identifying information will not be used in reports or publications resulting from this study. The FDA or other authorized agencies (e.g., qualified monitors from MSKCC or the NCI, etc.) may review patient records and pathology slides, as required.

17.1 Privacy

MSKCC's Privacy Office may allow the use and disclosure of protected health information pursuant to a completed and signed Research Authorization form. The use and disclosure of protected health information will be limited to the individuals described in the Research Authorization form. A Research Authorization form must be completed by the Principal Investigator and approved by the IRB/PB.

The consent indicates that individualized de identified information collected for the purposes of this study may be shared with other qualified researchers. Only researchers who have received approval from MSK will be allowed to access this information which will not include protected health information, such as the participant's name, except for dates. It is also stated in the Research Authorization that their research data may be shared with other qualified researchers.

The consent indicates that samples and genetic information collected may be shared with other qualified researchers and placed in online databases. An example of an online database is the NIH dbGAP database, which is monitored by the National Institutes of Health, and may be made accessible to investigators approved by the U.S. government. Such information will not include identifying information such as name. It is also stated in the Research Authorization that research data (e.g. genomic sequence) may be shared with regulators.

The requirements for submission of genotype/phenotype data into the NIH dbGAP or any other public database will be followed as per the IRB SOP for Genomic Data Sharing.

17.2 Serious Adverse Event (SAE) Reporting

ASSESSMENT OF SAFETY

Specification of Safety Variables

Safety assessments will consist of monitoring and reporting adverse events (AEs) and serious
adverse events (SAEs) per protocol. This includes all events of death, and any study specific
issue of concern.

Adverse Events

An AE is any unfavorable and unintended sign (including an abnormal laboratory

finding),
symptom, or disease temporally associated with the use of an investigational medicinal product
(IMP) or other protocol-imposed intervention, regardless of attribution.

This includes the following:

- AEs not previously observed in the subject that emerge during the protocol-specified
AE reporting period, including signs or symptoms associated with
Pancreatic cancer that were not present prior to the AE reporting period.
- Complications that occur as a result of protocol-mandated interventions (e.g., invasive procedures such as cardiac catheterizations)
- If applicable, AEs that occur prior to assignment of study treatment associated with medication washout, no treatment run-in, or other protocol-mandated intervention.
- Preexisting medical conditions (other than the condition being studied) judged by the investigator to have worsened in severity or frequency or changed in character during the protocol-specified AE reporting period.

Serious Adverse Events

An adverse event is considered serious if it results in ANY of the following outcomes:

- Death
- A life-threatening adverse event
- An adverse event that results in inpatient hospitalization or prolongation of existing hospitalization
- A persistent or significant incapacity or substantial disruption of the ability to conduct normal life functions
- A congenital anomaly/birth defect
- Important Medical Events (IME) that may not result in death, be life threatening, or require hospitalization may be considered serious when, based upon medical judgment, they may jeopardize the patient or participant and may require medical or surgical intervention to prevent one of the outcomes listed in this definition

Note: Hospital admission for a planned procedure/disease treatment is not considered an SAE.

Please note: Any SAE that occurs prior to the start of investigational treatment/intervention and is related to a screening test or procedure (i.e., a screening biopsy) must be reported.

All SAEs must be submitted in PIMS. If an SAE requires submission to the HRPP office per IRB SOP RR-408 ‘Reporting of Serious Adverse Events’, the SAE report must be submitted within 5 calendar days of the event. All other SAEs must be submitted within 30 calendar days of the event.

The report should contain the following information:

- The date the adverse event occurred
- The adverse event

- The grade of the event
- Relationship of the adverse event to the treatment(s)
- If the AE was expected
- Detailed text that includes the following
    - An explanation of how the AE was handled
    - A description of the participant's condition
    - Indication if the participant remains on the study
- If an amendment will need to be made to the protocol and/or consent form
- If the SAE is an Unanticipated Problem

For IND/IDE protocols:
The SAE report should be completed as per above instructions. If appropriate, the report will be forwarded to the FDA by the IND Office

To determine reporting requirements for single adverse event cases, the expectedness of these events will be assessed by using the following reference documents:

- RO7198457 Investigator's Brochure
- Atezolizumab Investigator's Brochure

Pancreatic surgery is a major operation that is associated with a 1.6% mortality and a 30-40% morbidity. All planned treatments under evaluation will not overlap with the recovery period from surgery. Therefore any mortality and morbidity related to pancreatic surgery will not be considered an SAE, and only SAE's after initiation of the investigational treatment will be reported.

Adverse Events of Special Interest (AESI)

<u>Non-Drug Specific Adverse Events of Special Interest (AESI):</u>

- Cases of potential drug-induced liver injury that include an elevated ALT or AST in combination with either an elevated bilirubin or clinical jaundice, as defined by Hy's law
    - Treatment-emergent ALT or AST > 3 x baseline value in combination with total bilirubin > 2 x ULN (of which ≥ 35% is direct bilirubin)
    - Treatment-emergent ALT or AST > 3 x baseline value in combination with clinical jaundice
- Suspected transmission of an infectious agent by the study drug, defined as follows:
    - Any organism, virus, or infectious particle (e.g., prion protein transmitting transmissible spongiform encephalopathy), pathogenic or non-pathogenic, is considered an infectious agent. A transmission of an infectious agent may be suspected from clinical symptoms or laboratory findings that indicate an infection in a patient exposed to a medicinal product. This term applies only when a contamination of the study drug is suspected.
- Any Dose Limiting Toxicity as defined in the protocol

Adverse Events of Special Interest Specific to RO7198457 and Atezolizumab:

- Systemic lupus erythematosus
- Nephritis
- Ocular toxicities (e.g., uveitis, retinitis, optic neuritis)
- Grade ≥ 2 cardiac disorders (e.g., atrial fibrillation, myocarditis, pericarditis)
- Vasculitis
- Autoimmune hemolytic anemia
- Severe cutaneous reactions (e.g., Stevens-Johnson syndrome, dermatitis bullous, toxic epidermal necrolysis)
- Events suggestive of hypersensitivity, infusion-related reactions, cytokine release syndrome, macrophage activating syndrome and hemophagocytic lymphohistiocytosis

Adverse Events of Special Interest Specific to RO7198457 (iNeST):

- Systemic reactions during or after study drug infusion if they meet the following severity criteria:
    - Grade ≥3 flu-like symptoms or Grade ≥ 3 IRR (infusion related reaction) according to NCI CTCAE v5.0
    - Grade ≥ 2 CRS according to NCI CTCAE v5.0

METHODS AND TIMING FOR ASSESSING AND RECORDING SAFETY VARIABLES

The investigator is responsible for ensuring that all AEs and SAEs that are observed or reported
during the study are collected and reported to the FDA, appropriate IRB(s), and Genentech, Inc.
in accordance with CFR 312.32 (IND Safety Reports).

Adverse Event Reporting Period

The study period during which AEs and SAEs where the patient has been exposed to Genentech and/or BioNTech product must be reported begins after informed consent is obtained and initiation of any study procedures and ends 90 days following the last administration of study treatment or study discontinuation/termination, whichever is earlier. After this period, investigators should only report SAEs that are attributed to prior study treatment

Assessment of Adverse Events

All AEs and SAEs whether volunteered by the subject, discovered by study personnel during questioning, or detected through physical examination, laboratory test, or other means will be reported appropriately. Each reported AE or SAE will be described by its duration (i.e., start and end dates), regulatory seriousness criteria if applicable, suspected relationship to atezolizumab and/or RO7198457 (see following guidance), and actions taken.

To ensure consistency of AE and SAE causality assessments, investigators should apply the following general guideline:

Yes

There is a plausible temporal relationship between the onset of the AE and administration of atezolizumab and/or RO7198457, and the AE cannot be readily explained by the subject's clinical state, intercurrent illness, or concomitant therapies; and/or the AE follows a known pattern of response to atezolizumab and/or RO7198457 or with similar treatments; and/or the AE abates or resolves upon discontinuation of atezolizumab and/or RO7198457 or dose reduction and, if applicable, reappears upon re-challenge.

No

Evidence exists that the AE has an etiology other than atezolizumab and/or RO7198457 (e.g., preexisting medical condition, underlying disease, intercurrent illness, or concomitant medication); and/or the AE has no plausible temporal relationship to atezolizumab and/or RO7198457 administration (e.g., cancer diagnosed 2 days after first dose of study drug).

Expected adverse events are those adverse events that are listed or characterized in the Package Insert (P.I)
or current Investigator Brochure (I.B).

Unexpected adverse events are those not listed in the P.I or current I.B or not identified. This includes adverse events for which the specificity or severity is not consistent with the description in the P.I. or I.B. For example, under this definition, hepatic necrosis would be unexpected if the P.I. or I.B. only referred to elevated hepatic enzymes or hepatitis.

For patients receiving combination therapy, causality will be assessed individually for each protocol-mandated therapy

PROCEDURES FOR ELICITING, RECORDING, AND REPORTING ADVERSE EVENTS

Eliciting Adverse Events

A consistent methodology for eliciting AEs at all subject evaluation time points should be adopted. Examples of non-directive questions include:

- "How have you felt since your last clinical visit?"
- "Have you had any new or changed health problems since you were last here?"

Specific Instructions for Recording Adverse Events

Investigators should use correct medical terminology/concepts when reporting AEs or SAEs. Avoid colloquialisms and abbreviations.

a. Diagnosis vs. Signs and Symptoms

If known at the time of reporting, a diagnosis should be reported rather than individual signs and symptoms (e.g., record only liver failure or hepatitis rather than jaundice, asterixis, and elevated transaminases). However, if a constellation of signs and/or symptoms cannot be medically characterized as a single diagnosis or syndrome at the time of reporting, it is acceptable to report the information that is currently available. If a diagnosis is subsequently established, it should be reported as follow-up information.

b. Deaths

All deaths that occur during the protocol-specified AE reporting period, regardless of attribution, will be reported to the appropriate parties. When recording a death, the event or condition that caused or contributed to the fatal outcome should be reported as the single medical concept. If the cause of death is unknown and cannot be ascertained at the time of reporting, report "Unexplained Death".

c. Preexisting Medical Conditions

A preexisting medical condition is one that is present at the start of the study. Such conditions should be reported as medical and surgical history. A preexisting medical condition should be re-assessed throughout the trial and reported as an AE or SAE only if the frequency, severity, or character of the condition worsens during the study. When reporting such events, it is important to convey the concept that the preexisting condition has changed by including applicable descriptors (e.g., "more frequent headaches").

d. Hospitalizations for Medical or Surgical Procedures

Any AE that results in hospitalization or prolonged hospitalization should be documented and reported as an SAE. If a subject is hospitalized to undergo a medical or surgical procedure as a result of an AE, the event responsible for the procedure, not the procedure itself, should be reported as the SAE. For example, if a subject is hospitalized to undergo coronary bypass surgery, record the heart condition that necessitated the bypass as the SAE.

Hospitalizations for the following reasons do not require reporting:

- Hospitalization or prolonged hospitalization for diagnostic or elective surgical procedures for preexisting conditions
- Hospitalization or prolonged hospitalization required to allow efficacy measurement for the study or
- Hospitalization or prolonged hospitalization for scheduled therapy of the target disease of the study

e. Assessment of Severity of Adverse Events

The adverse event severity grading scale for the NCI CTCAE (v5.0) Update current versions) will be used for assessing adverse event severity. Below Table should be used for assessing severity for adverse events that are not specifically listed in the NCI CTCAE.

Adverse Event Severity Grading Scale for Events Not Specifically Listed in NCI CTCAE

Grade	Severity
1	Mild; asymptomatic or mild symptoms; clinical or diagnostic observations only; or intervention not indicated
2	Moderate; minimal, local, or non-invasive intervention indicated; or limiting age-appropriate instrumental activities of daily living [a]
3	Severe or medically significant, but not immediately life-threatening; hospitalization or prolongation of hospitalization indicated; disabling; or limiting self-care activities of daily living [b,c]
4	Life-threatening consequences or urgent intervention indicated d
5	Death related to adverse event [d]

NCI CTCAE = National Cancer Institute Common Terminology Criteria for Adverse Events.

Note: Based on the most recent version of NCI CTCAE (v5.0), which can be found at: http://ctep.cancer.gov/protocolDevelopment/electronic_applications/ctc.htm

a. Instrumental activities of daily living refer to preparing meals, shopping for groceries or clothes, using the telephone, managing money, etc.
b. Examples of self-care activities of daily living include bathing, dressing and undressing, feeding oneself, using the toilet, and taking medications, as performed by patients who are not bedridden.
c. If an event is assessed as a "significant medical event," it must be reported as a serious adverse event
d. Grade 4 and 5 events must be reported as serious adverse events

f. Pregnancy

If a female subject becomes pregnant while receiving the study drug or within *5 months* after the last dose of study drug, a report should be completed and expeditiously submitted to Genentech, Inc and BioNTech. Follow-up to obtain the outcome of the pregnancy should also occur. Abortion, whether accidental, therapeutic, or spontaneous, should always be classified as serious, and expeditiously reported as an SAE. Similarly, any congenital anomaly/birth defect in a child born to a female subject exposed to the study drug should be reported as an SAE.

g. Post-Study Adverse Events

For studies involving collection of survival data the investigator after the end of the adverse event reporting period (defined as 90 days after the last dose of study drug) (Refer to section 11) should report all deaths, (regardless of cause), and any serious adverse event including development of cancer or a congenital anomaly in a subsequently conceived offspring of a female subject that is believed to be related to prior exposure to study drug.
Case Transmission Verification will be performed by both parties during this period to ensure successful transmission of Single case reports

17.2.1 Serious adverse reporting to Genentech and BioNTech

Case Transmission Verification of Single Case Reports

The Sponsor agrees to conduct the Case Transmission verification to ensure that all single case reports have been adequately received by Genentech via *MSKCC* emailing Genentech and BioNTech a Quarterly line-listing documenting single case reports sent by *MSKCC* to Genentech and BioNTech in the preceding time period.
The periodic line-listing will be exchanged within seven (7) calendar days of the end of the agreed time period. Confirmation of receipt should be received within the time period mutually agreed upon. This line listing will only include: SAEs, AESIs, Special Situations and pregnancy reports.

If discrepancies are identified, the Sponsor, Genentech and BioNTech will cooperate in resolving the discrepancies. The responsible individuals for each party shall handle the matter on a case-by-case basis until satisfactory resolution. The sponsor shall receive reconciliation guidance documents within the 'Activation Package'.

Following Case Transmission Verification, single case reports which have not been received by Genentech and BioNTech shall be forwarded by MSKCC to Genentech and BioNTech within five (5) calendar days from request by Genentech or BioNTech

At the end of the study, a final cumulative Case Transmission Verification report will be sent to Genentech and BioNTech

j. Exchange OF SINGLE CASE REPORTS
MSKCC will be responsible for collecting all protocol-defined Adverse Events (AEs)/Serious Adverse Events (SAEs), AEs of Special Interest (AESIs), Special Situation Reports (including pregnancy reports) and Product Complaints (with or without an AE) originating from the Study for the Product.

Investigators must report only SAEs, AEs of Special Interest (AESIs), Special Situation Reports (including pregnancies) and Product Complaints as single case reports adequately to Genentech and BioNTech within the timelines described below. The completed de-identified MSKCC SAE reporting form (with patient protocol ID) should be faxed/emailed immediately upon completion to Genentech at the following contacts:

All protocol-defined SAEs, AESIs, Special Situation Reports (including pregnancy reports) and Product Complaints *with* an AE should be sent to:

Fax: 650-238-6067
Email: usds_aereporting-d@gene.com and pharmacovigilance@biontech.de

All Product Complaints *without* an AE should call:

PC Hotline Number: (800) 334-0290 (M-F: 5 am to 5 pm PST)

It is understood and agreed that the Sponsor will be responsible for the evaluation of AEs/SAEs, AESIs, Special Situation Reports (including pregnancy reports) and Product Complaints (with or without an AE) originating from the study.
These single case reports will be exchanged between the parties as outlined below so that regulatory obligations are met.

Serious adverse events (SAEs), AEs of Special Interest (AESIs), pregnancy reports, other Special Situation Reports and Product Complaints (with or without an AE), where the patient has been exposed to the Genentech and/or BioNTech Product, will be sent on a

de-identified MSKCC SAE reporting form (with patient protocol ID) approved by Genentech to Genentech Drug Safety and BioNTech. Transmission of these reports (initial and follow-up) will be either electronically or by fax and within the timelines specified below:

- **SADRs**

  Serious AE reports that are related to the Product shall be transmitted to Genentech and BioNTech within 5 business days of the awareness date.

- **Other SAEs**

  Serious AE reports that are unrelated to the Product shall be transmitted to Genentech and BioNTech within 5 business days of the awareness date.

- **AESIs**
  AESIs requiring expedited reporting (related or possibly related to the Products or where the causality is assessed as unknown or not provided) shall be forwarded to Genentech and BioNTech within five (5) business days of the awareness date. Others (non-related to the products) shall be sent within thirty (30) calendar days. The list of AESIs is documented in the study protocol.

- **Special Situation Reports**
  Pregnancy reports
  While such reports are not serious AEs or Adverse Drug Reactions (ADRs) per se, as defined herein, any reports of pregnancy, where the fetus may have been exposed to the Product, shall be transmitted to Genentech and BioNTech within 5 business days of the awareness date. Pregnancies will be followed up until the outcome of the pregnancy is known, whenever possible, based upon due diligence taken to obtain the follow-up information.

  Pregnancies in Female Partners of Male Patients
  Male patients will be instructed through the Informed Consent Form to immediately inform the investigator if their partner becomes pregnant during the study or within 30 days after the last dose of study drug. If the partner of a male patient is willing to voluntarily provide further information-that information will be reported by a MSKCC SAE report which will be de-identified and submitted to Genentech and BioNTech within thirty (30) calendar days of the awareness date.

- **Other Special Situation Reports**
  In addition to all SAEs, pregnancy reports and AESIs, the following other Special Situations Reports should be collected even in the absence of an Adverse Event and transmitted to Genentech within thirty (30) calendar days:
  - Data related to the Product usage during breastfeeding
  - Data related to overdose, abuse, misuse or medication error (including potentially exposed or intercepted medication errors)
  - In addition, reasonable attempts should be made to obtain and submit the age or age group of the patient, in order to be able to identify potential safety signals specific to a particular population

- **Product Complaints**

All Product Complaints (with or without an AE) for Atezolizumab shall be forwarded to Genentech within fifteen (15) calendar days of the awareness date. A Product Complaint is defined as any written or oral information received from a complainant that alleges deficiencies related to identity, quality, safety, strength, purity, reliability, durability, effectiveness, or performance of a product after it has been released and distributed to the commercial market or clinical trial.

Reporting to Regulatory Authorities, Ethics Committees and Investigators

- MSKCC, as the Sponsor of the Study, will be responsible for the expedited reporting of safety reports originating from the Study to the Regulatory Authorities (FDA) where it has filed a clinical trial approval, in compliance with local regulations. (Remove if Study is an IND-exempt)
- MSKCC, will be responsible for the expedited reporting of safety reports originating from the Study to the Ethics Committees and Institutional Review Boards (IRB), where applicable.
- MSKCC, will be responsible for the distribution of safety information to its own investigators, where relevant, in accordance with local regulations.

All written IND Safety Reports submitted to the FDA by the Investigator must also be faxed to
Genentech Drug Safety:

Fax: (650) 225-4682 or (650) 225-4630

For questions related to safety reporting, please contact Genentech Drug Safety:

Tel: (888) 835-2555
Fax: (650) 225-4682 or (650) 225-4630

AGGREGATE REPORTS

MSKCC, as the Sponsor of the Study, will be responsible for the preparation of their own Development Safety Update Report (DSUR) for the Study and for the submission of the report to the regulatory authorities and Ethics Committees of the concerned Member States, where applicable. *MSKCC*, agrees to share a copy of their own DSUR with Genentech and BioNtech as soon as reasonably possible after completion.

Genentech agrees to forward to MSKCC an executive summary of the Genentech DSUR upon request from MSKCC. BioNTech agrees to disclose relevant portions of the BioNTech DSUR as shared to Genentech upon request from MSKCC. Furthermore, Genentech and BioNTech agree that MSKCC may cross-reference the executive summary of the Genentech DSUR, as applicable.

Other Reports

MSKCC will forward a copy of the Final Study Report to Genentech and BioNTech upon completion of the Study. *MSKCC* will forward a copy of the Publication to Genentech and BioNTech upon completion of the Study.

STUDY CLOSE-OUT

Any study report submitted to the FDA by the Sponsor-Investigator should be copied to Genentech. This includes all IND annual reports and the Clinical Study Report (final study report). Additionally, any literature articles that are a result of the study should be sent to Genentech. Copies of such reports should be mailed to the assigned Clinical Operations contact for the study:

Luke Passler

Clinical Study Manager

Email: Passlerl@gene.com

And to Genentech Drug Safety CTV oversight mail box at:
ctvist_drugsafety@gene.com and pharmacovigilance@biontech.de

SAFETY CRISIS MANAGEMENT

In case of a safety crisis, e.g., where safety issues have a potential impact on the indication(s), on the conduct of the Study, may lead to labeling changes or regulatory actions that limit or restrict the way in which the Product is used, or where there is media involvement, the Party where the crisis originates will contact the other Party as soon as possible.

The Parties agree that Genentech shall have the final say and control over safety crisis management issues relating to the Product. MKSCC agrees that it shall not answer such queries from media and other sources relating to the Product but shall redirect such queries to Genentech

18.0 INFORMED CONSENT PROCEDURES

Before protocol-specified procedures are carried out, consenting professionals will explain full details of the protocol and study procedures as well as the risks involved to participants prior to their inclusion in the study. Participants will also be informed that they are free to withdraw from the study at any time. All participants must sign an IRB/PB-approved consent form indicating their consent to participate. This consent form meets the requirements of the Code of Federal Regulations and the IRB/PB of this Center. The consent form will include the following:

1. The nature and objectives, potential risks and benefits of the intended study.
2. The length of study and the likely follow-up required.
3. Alternatives to the proposed study. (This will include available standard and investigational therapies. In addition, patients will be offered an option of supportive care for therapeutic studies.)
4. The name of the investigator(s) responsible for the protocol.
5. The right of the participant to accept or refuse study interventions/interactions and to withdraw from participation at any time.

Before any protocol-specific procedures can be carried out, the consenting professional will fully explain the aspects of patient privacy concerning research specific information. In addition to signing the IRB Informed Consent, all patients must agree to the Research Authorization component of the informed consent form.

Each participant and consenting professional will sign the consent form. The participant must receive a copy of the signed informed consent form.

19.0 REFERENCES

1. Siegel RL, Miller KD, Jemal A. Cancer statistics, 2016. *CA Cancer J Clin.* 2016;66(1):7-30.
2. Rahib L, Smith BD, Aizenberg R, Rosenzweig AB, Fleshman JM, Matrisian LM. Projecting cancer incidence and deaths to 2030: the unexpected burden of thyroid, liver, and pancreas cancers in the United States. *Cancer Res.* 2014;74(11):2913-2921.
3. He J, Ahuja N, Makary MA, et al. 2564 resected periampullary adenocarcinomas at a single institution: trends over three decades. *HPB (Oxford).* 2014;16(1):83-90.
4. Ferrone CR, Brennan MF, Gonen M, et al. Pancreatic adenocarcinoma: the actual 5-year survivors. *J Gastrointest Surg.* 2008;12(4):701-706.
5. Ferrone CR, Pieretti-Vanmarcke R, Bloom JP, et al. Pancreatic ductal adenocarcinoma: long-term survival does not equal cure. *Surgery.* 2012;152(3 Suppl 1):S43-49.
6. Katz MH, Wang H, Fleming JB, et al. Long-term survival after multidisciplinary management of resected pancreatic adenocarcinoma. *Ann Surg Oncol.* 2009;16(4):836-847.
7. Oettle H, Post S, Neuhaus P, et al. Adjuvant chemotherapy with gemcitabine vs observation in patients undergoing curative-intent resection of pancreatic cancer: a randomized controlled trial. *JAMA.* 2007;297(3):267-277.
8. Neoptolemos JP, Palmer DH, Ghaneh P, et al. Comparison of adjuvant gemcitabine and capecitabine with gemcitabine monotherapy in patients with resected pancreatic cancer (ESPAC-4): a multicentre, open-label, randomised, phase 3 trial. *Lancet.* 2017;389(10073):1011-1024.
9. Von Hoff DD, Ervin T, Arena FP, et al. Increased survival in pancreatic cancer with nab-paclitaxel plus gemcitabine. *N Engl J Med.* 2013;369(18):1691-1703.
10. Tempero MA, Cardin DB, Goldstein D, et al. APACT: Phase III randomized trial of adjuvant treatment with nab-paclitaxel (nab-P) plus gemcitabine (Gem) versus Gem alone in patients (pts) with resected pancreatic cancer (PC). *Journal of Clinical Oncology.* 2016;34(4_suppl):TPS473-TPS473.
11. Topalian SL, Hodi FS, Brahmer JR, et al. Safety, activity, and immune correlates of anti-PD-1 antibody in cancer. *N Engl J Med.* 2012;366(26):2443-2454.
12. El-Khoueiry AB, Sangro B, Yau T, et al. Nivolumab in patients with advanced hepatocellular carcinoma (CheckMate 040): an open-label, non-comparative, phase 1/2 dose escalation and expansion trial. *Lancet.* 2017;389(10088):2492-2502.
13. Le DT, Uram JN, Wang H, et al. PD-1 Blockade in Tumors with Mismatch-Repair Deficiency. *N Engl J Med.* 2015;372(26):2509-2520.
14. Seiwert TY, Burtness B, Weiss J, et al. Inflamed-phenotype gene expression signatures to predict benefit from the anti-PD-1 antibody pembrolizumab in PD-L1+ head and neck cancer patients. *Journal of Clinical Oncology.* 2015;33(15_suppl):6017-6017.
15. Muro K, Bang Y, Shankaran V, et al. LBA15A PHASE 1B STUDY OF PEMBROLIZUMAB (PEMBRO; MK-3475) IN PATIENTS (PTS) WITH ADVANCED GASTRIC CANCER. *Annals of Oncology.* 2014;25(suppl_4):mdu438.415-mdu438.415.
16. Kang YK, Boku N, Satoh T, et al. Nivolumab in patients with advanced gastric or gastro-oesophageal junction cancer refractory to, or intolerant of, at least two previous chemotherapy regimens (ONO-4538-12, ATTRACTION-2): a randomised, double-blind, placebo-controlled, phase 3 trial. *Lancet.* 2017;390(10111):2461-2471.
17. Nanda R, Chow LQ, Dees EC, et al. Pembrolizumab in Patients With Advanced Triple-Negative Breast Cancer: Phase Ib KEYNOTE-012 Study. *J Clin Oncol.* 2016;34(21):2460-2467.
18. Topalian SL, Drake CG, Pardoll DM. Immune checkpoint blockade: a common denominator approach to cancer therapy. *Cancer Cell.* 2015;27(4):450-461.
19. Snyder A, Makarov V, Merghoub T, et al. Genetic basis for clinical response to CTLA-4 blockade in melanoma. *N Engl J Med.* 2014;371(23):2189-2199.
20. Rizvi NA, Hellmann MD, Snyder A, et al. Cancer immunology. Mutational landscape determines sensitivity to PD-1 blockade in non-small cell lung cancer. *Science.* 2015;348(6230):124-128.
21. Brahmer JR, Tykodi SS, Chow LQ, et al. Safety and activity of anti-PD-L1 antibody in patients with advanced cancer. *N Engl J Med.* 2012;366(26):2455-2465.
22. Feig C, Jones JO, Kraman M, et al. Targeting CXCL12 from FAP-expressing carcinoma-associated fibroblasts synergizes with anti-PD-L1 immunotherapy in pancreatic cancer. *Proc Natl Acad Sci U S A.* 2013;110(50):20212-20217.

23. Winograd R, Byrne KT, Evans RA, et al. Induction of T-cell Immunity Overcomes Complete Resistance to PD-1 and CTLA-4 Blockade and Improves Survival in Pancreatic Carcinoma. *Cancer Immunol Res.* 2015;3(4):399-411.
24. Schumacher TN, Schreiber RD. Neoantigens in cancer immunotherapy. *Science.* 2015;348(6230):69-74.
25. Carreno BM, Magrini V, Becker-Hapak M, et al. Cancer immunotherapy. A dendritic cell vaccine increases the breadth and diversity of melanoma neoantigen-specific T cells. *Science.* 2015;348(6236):803-808.
26. Sahin U, Derhovanessian E, Miller M, et al. Personalized RNA mutanome vaccines mobilize poly-specific therapeutic immunity against cancer. *Nature.* 2017;547(7662):222-226.
27. Ott PA, Hu Z, Keskin DB, et al. An immunogenic personal neoantigen vaccine for patients with melanoma. *Nature.* 2017;547(7662):217-221.
28. Gubin MM, Zhang X, Schuster H, et al. Checkpoint blockade cancer immunotherapy targets tumour-specific mutant antigens. *Nature.* 2014;515(7528):577-581.
29. Yadav M, Jhunjhunwala S, Phung QT, et al. Predicting immunogenic tumour mutations by combining mass spectrometry and exome sequencing. *Nature.* 2014;515(7528):572-576.
30. Kreiter S, Vormehr M, van de Roemer N, et al. Mutant MHC class II epitopes drive therapeutic immune responses to cancer. *Nature.* 2015;520(7549):692-696.
31. Bailey P, Chang DK, Forget MA, et al. Exploiting the neoantigen landscape for immunotherapy of pancreatic ductal adenocarcinoma. *Sci Rep.* 2016;6:35848.
32. Balli D, Rech AJ, Stanger BZ, Vonderheide RH. Immune Cytolytic Activity Stratifies Molecular Subsets of Human Pancreatic Cancer. *Clin Cancer Res.* 2017;23(12):3129-3138.
33. Balachandran VP, Luksza M, Zhao JN, et al. Identification of unique neoantigen qualities in long-term survivors of pancreatic cancer. *Nature.* 2017;551(7681):512-516.
34. Johansson H, Andersson R, Bauden M, Hammes S, Holdenrieder S, Ansari D. Immune checkpoint therapy for pancreatic cancer. *World J Gastroenterol.* 2016;22(43):9457-9476.
35. Ribas A, Hamid O, Daud A, et al. Association of Pembrolizumab With Tumor Response and Survival Among Patients With Advanced Melanoma. *JAMA.* 2016;315(15):1600-1609.
36. Hutcheson J, Balaji U, Porembka MR, et al. Immunologic and Metabolic Features of Pancreatic Ductal Adenocarcinoma Define Prognostic Subtypes of Disease. *Clin Cancer Res.* 2016;22(14):3606-3617.
37. Balar AV, Galsky MD, Rosenberg JE, et al. Atezolizumab as first-line treatment in cisplatin-ineligible patients with locally advanced and metastatic urothelial carcinoma: a single-arm, multicentre, phase 2 trial. *Lancet.* 2017;389(10064):67-76.
38. Rittmeyer A, Barlesi F, Waterkamp D, et al. Atezolizumab versus docetaxel in patients with previously treated non-small-cell lung cancer (OAK): a phase 3, open-label, multicentre randomised controlled trial. *Lancet.* 2017;389(10066):255-265.
39. Conroy T, Desseigne F, Ychou M, et al. FOLFIRINOX versus gemcitabine for metastatic pancreatic cancer. *N Engl J Med.* 2011;364(19):1817-1825.
40. Okada KI, Shimokawa T, Hirono S, et al. Effect of Neoadjuvant Nab-Paclitaxel plus Gemcitabine Therapy on Overall Survival in Patients with Borderline Resectable Pancreatic Cancer: A Prospective Multicenter Phase II Trial (NAC-GA Trial). *Oncology.* 2017.
41. Ielpo B, Duran H, Diaz E, et al. Preoperative treatment with gemcitabine plus nab-paclitaxel is a safe and effective chemotherapy for pancreatic adenocarcinoma. *Eur J Surg Oncol.* 2016;42(9):1394-1400.
42. Heinrich S, Pestalozzi B, Lesurtel M, et al. Adjuvant gemcitabine versus NEOadjuvant gemcitabine/oxaliplatin plus adjuvant gemcitabine in resectable pancreatic cancer: a randomized multicenter phase III study (NEOPAC study). *BMC Cancer.* 2011;11:346.
43. Hiraoka N, Ino Y, Yamazaki-Itoh R, Kanai Y, Kosuge T, Shimada K. Intratumoral tertiary lymphoid organ is a favourable prognosticator in patients with pancreatic cancer. *Br J Cancer.* 2015;112(11):1782-1790.
44. Lu C, Paschall AV, Shi H, et al. The MLL1-H3K4me3 Axis-Mediated PD-L1 Expression and Pancreatic Cancer Immune Evasion. *J Natl Cancer Inst.* 2017;109(6).
45. Imai D, Yoshizumi T, Okano S, et al. The prognostic impact of programmed cell death ligand 1 and human leukocyte antigen class I in pancreatic cancer. *Cancer Med.* 2017;6(7):1614-1626.

46. Nomi T, Sho M, Akahori T, et al. Clinical significance and therapeutic potential of the programmed death-1 ligand/programmed death-1 pathway in human pancreatic cancer. *Clin Cancer Res.* 2007;13(7):2151-2157.
47. Royal RE, Levy C, Turner K, et al. Phase 2 trial of single agent Ipilimumab (anti-CTLA-4) for locally advanced or metastatic pancreatic adenocarcinoma. *J Immunother.* 2010;33(8):828-833.
48. Zitvogel L, Apetoh L, Ghiringhelli F, Kroemer G. Immunological aspects of cancer chemotherapy. *Nat Rev Immunol.* 2008;8(1):59-73.
49. Gibney GT, Kudchadkar RR, DeConti RC, et al. Safety, correlative markers, and clinical results of adjuvant nivolumab in combination with vaccine in resected high-risk metastatic melanoma. *Clin Cancer Res.* 2015;21(4):712-720.
50. Le DT, Lutz E, Uram JN, et al. Evaluation of ipilimumab in combination with allogeneic pancreatic tumor cells transfected with a GM-CSF gene in previously treated pancreatic cancer. *J Immunother.* 2013;36(7):382-389.
51. Hartmaier RJ, Charo J, Fabrizio D, et al. Genomic analysis of 63,220 tumors reveals insights into tumor uniqueness and targeted cancer immunotherapy strategies. *Genome Med.* 2017;9(1):16.
52. Luksza M, Riaz N, Makarov V, et al. A neoantigen fitness model predicts tumour response to checkpoint blockade immunotherapy. *Nature.* 2017;551(7681):517-520.
53. Tran E, Robbins PF, Lu YC, et al. T-Cell Transfer Therapy Targeting Mutant KRAS in Cancer. *N Engl J Med.* 2016;375(23):2255-2262.
54. Verdegaal EM, de Miranda NF, Visser M, et al. Neoantigen landscape dynamics during human melanoma-T cell interactions. *Nature.* 2016;536(7614):91-95.
55. Kranz LM, Diken M, Haas H, et al. Systemic RNA delivery to dendritic cells exploits antiviral defence for cancer immunotherapy. *Nature.* 2016;534(7607):396-401.
56. Zitvogel L, Galluzzi L, Kepp O, Smyth MJ, Kroemer G. Type I interferons in anticancer immunity. *Nat Rev Immunol.* 2015;15(7):405-414.
57. Wille-Reece U, Wu CY, Flynn BJ, Kedl RM, Seder RA. Immunization with HIV-1 Gag protein conjugated to a TLR7/8 agonist results in the generation of HIV-1 Gag-specific Th1 and CD8+ T cell responses. *J Immunol.* 2005;174(12):7676-7683.
58. Wille-Reece U, Flynn BJ, Lore K, et al. Toll-like receptor agonists influence the magnitude and quality of memory T cell responses after prime-boost immunization in nonhuman primates. *J Exp Med.* 2006;203(5):1249-1258.
59. Kreiter S, Selmi A, Diken M, et al. Intranodal vaccination with naked antigen-encoding RNA elicits potent prophylactic and therapeutic antitumoral immunity. *Cancer Res.* 2010;70(22):9031-9040.
60. Kreiter S, Selmi A, Diken M, et al. Increased antigen presentation efficiency by coupling antigens to MHC class I trafficking signals. *J Immunol.* 2008;180(1):309-318.
61. Herbst RS, Soria JC, Kowanetz M, et al. Predictive correlates of response to the anti-PD-L1 antibody MPDL3280A in cancer patients. *Nature.* 2014;515(7528):563-567.
62. Mirkin KA, Greenleaf EK, Hollenbeak CS, Wong J. Time to the initiation of adjuvant chemotherapy does not impact survival in patients with resected pancreatic cancer. *Cancer.* 2016;122(19):2979-2987.
63. Valle JW, Palmer D, Jackson R, et al. Optimal duration and timing of adjuvant chemotherapy after definitive surgery for ductal adenocarcinoma of the pancreas: ongoing lessons from the ESPAC-3 study. *J Clin Oncol.* 2014;32(6):504-512.
64. Xia BT, Ahmad SA, Al Humaidi AH, et al. Time to Initiation of Adjuvant Chemotherapy in Pancreas Cancer: A Multi-Institutional Experience. *Ann Surg Oncol.* 2017;24(9):2770-2776.
65. Burris HA, 3rd, Moore MJ, Andersen J, et al. Improvements in survival and clinical benefit with gemcitabine as first-line therapy for patients with advanced pancreas cancer: a randomized trial. *J Clin Oncol.* 1997;15(6):2403-2413.
66. Oettle H, Neuhaus P, Hochhaus A, et al. Adjuvant chemotherapy with gemcitabine and long-term outcomes among patients with resected pancreatic cancer: the CONKO-001 randomized trial. *JAMA.* 2013;310(14):1473-1481.
67. Cheng ML, Fong L. Effects of RANKL-Targeted Therapy in Immunity and Cancer. *Front Oncol.* 2014;3:329.
68. Dindo D, Demartines N, Clavien PA. Classification of surgical complications: a new proposal with evaluation in a cohort of 6336 patients and results of a survey. *Ann Surg.* 2004;240(2):205-213.

69. Lutz E, Yeo CJ, Lillemoe KD, et al. A lethally irradiated allogeneic granulocyte-macrophage colony stimulating factor-secreting tumor vaccine for pancreatic adenocarcinoma. A Phase II trial of safety, efficacy, and immune activation. *Ann Surg.* 2011;253(2):328-335.
70. Lee DW, Gardner R, Porter DL, et al. Current concepts in the diagnosis and management of cytokine release syndrome. *Blood.* 2014;124(2):188-195.
71. Wolchok JD, Kluger H, Callahan MK, et al. Nivolumab plus ipilimumab in advanced melanoma. *N Engl J Med.* 2013;369(2):122-133.
72. Larkin J, Chiarion-Sileni V, Gonzalez R, et al. Combined Nivolumab and Ipilimumab or Monotherapy in Untreated Melanoma. *N Engl J Med.* 2015;373(1):23-34.
73. Antonia S, Goldberg SB, Balmanoukian A, et al. Safety and antitumour activity of durvalumab plus tremelimumab in non-small cell lung cancer: a multicentre, phase 1b study. *Lancet Oncol.* 2016;17(3):299-308.
74. Di Giacomo AM, Biagioli M, Maio M. The emerging toxicity profiles of anti-CTLA-4 antibodies across clinical indications. *Semin Oncol.* 2010;37(5):499-507.
75. Weber JS, Kahler KC, Hauschild A. Management of Immune-mediated adverse events and kinetics of response with ipilimumab. *J Clin Oncol.* 2012;30(21):2691-2697.
76. Oken MM, Creech RH, Tormey DC, et al. Toxicity and response criteria of the Eastern Cooperative Oncology Group. *Am J Clin Oncol.* 1982;5(6):649-655.
77. Von Hoff DD, Ervin T, Arena FP, et al. Increased survival in pancreatic cancer with nab-paclitaxel plus gemcitabine. *N Engl J Med*. 2013;369(18):1691–1703.
78. Conroy T, Desseigne F, Ychou M, Bouché O, Guimbaud R, Bécouarn Y, et al. FOLFIRINOX versus gemcitabine for metastatic pancreatic cancer. *N Engl J Med*. 2011;364:1817–25.
79. Conroy T, Hammel P, Hebbar M, Abdelghani BM, Wei AC, Raoul JL, et al. FOLFIRINOX or Gemcitabine as Adjuvant Therapy for Pancreatic Cancer. *N Engl J Med*. 2018;379(25):2395–406
80. Aurélie Collignon, Françoise Silvy, Stéphane Robert, Malika Trad, Sébastien Germain, Jérémy Nigri, Frédéric André, Véronique Rigot, Richard Tomasini, Bernard Bonnotte, Dominique Lombardo, Eric Mas & Evelyne Beraud (2018) Dendritic cell-based vaccination: powerful resources of immature dendritic cells against pancreatic adenocarcinoma, *OncoImmunology*, 7:12

20.0 APPENDICES

Appendix 1. Eastern Cooperative Oncology Group (ECOG) Scale of Performance Status[76]

Grade	ECOG Performance Status
0	Fully active, able to carry on all pre-disease performance without restriction
1	Restricted in physically strenuous activity but ambulatory and able to carry out work of a light sedentary nature, e.g., light house work, office work
2	Ambulatory and capable of self-care but unable to carry out any work activities; up and about more than 50% of waking hours
3	Capable of only limited self-care; confined to bed or chair more than 50% of waking hours
4	Completely disabled; cannot carry on any self-care; totally confined to bed or chair
5	Dead

Appendix 2. Clavien-Dindo Classification of Postoperative Complications[68]

Grade	Definition
Grade I	Any deviation from the normal postoperative course without the need for pharmacological treatment or surgical, endoscopic, and radiological interventions<br>Allowed therapeutic regimens are: drugs as antiemetics, antipyretics, analgesics, diuretics, electrolytes, and physiotherapy. This grade also includes wound infections opened at the bedside
Grade II	Requiring pharmacological treatment with drugs other than such allowed for grade I complications<br>Blood transfusion and total parenteral nutrition are also included
Grade III<br>Grade IIIa<br>Grade IIIb	Requiring surgical, endoscopic, and radiological intervention<br>Intervention not under general anesthesia<br>Intervention under general anesthesia
Grade IV<br>Grade IVa<br>Grade IVb	Life-threatening complication (including CNS complications)* requiring ICU management<br>Single organ dysfunction (including dialysis)<br>Multiorgan dysfunction
Grade V	Death of a patient

*Brain hemorrhage, ischemic stroke, subarachnoid bleeding, but excluding transient ischemic attacks
CNS, central nervous system; ICU, intensive care unit

Appendix 3. Pre-Existing Autoimmune Diseases

Patients with a convincing history of any autoimmune disease listed in the table below are excluded from participating in the study. Possible exceptions to this exclusion could be patients with a medical history of such entities as atopic disease or childhood arthralgias where the clinical suspicion of autoimmune disease is low. Patients with a history of autoImmune-mediated hypothyroidism on a stable dose of thyroid-replacement hormone may be eligible for this study. Patients with a history of manageable, reversible Immune-mediated AEs on prior immunotherapy may be eligible after consultation with the DMT Safety Committee. In addition, transient autoimmune manifestations of an acute infectious disease that resolved upon treatment of the infectious agent are not excluded (e.g., acute Lyme arthritis).

**Examples of Autoimmune Diseases and Immune Deficiencies:**
Acute disseminated encephalomyelitis
Addison's disease
Ankylosing spondylitis
Antiphospholipid antibody syndrome
Aplastic anemia
Autoimmune hemolytic anemia
Autoimmune hepatitis
Autoimmune hypoparathyroidism
Autoimmune hypophysitis
Autoimmune myocarditis
Autoimmune oophoritis
Autoimmune orchitis
Autoimmune thrombocytopenic purpura
Behcet's disease
Bullous pemphigoid
Chronic inflammatory demyelinating polyneuropathy
Churg-Strauss syndrome
Crohn's disease
Dermatomyositis
Diabetes Mellitus (Type 1)
Type 1 Dysautonomia
Epidermolysis bullosa acquisita
Gestational pemphigoid
Giant-cell arteritis
Goodpasture's syndrome
Graves' disease
Guillain-Barre syndrome
Hashimoto's thyroiditis
IgA nephropathy
Inflammatory bowel disease
Interstitial cystitis
Kawasaki's disease
Lambert-Eaton myasthenia syndrome
Lupus erythematosus
Lyme disease
Chronic Meniere's syndrome
Mooren's ulcer
Morphea
Multiple sclerosis
Myasthenia gravis
Neuromyotonia
Opsoclonus myoclonus syndrome
Optic neuritis
Ord's thyroiditis
Pemphigus
Pernicious anemia
Polyarteritis nodosa

Polyarthritis
Polyglandular autoimmune syndrome
Primary biliary cirrhosis
Psoriasis
Reiter's syndrome
Rheumatoid arthritis
Sarcoidosis
Scleroderma
Sjögren's syndrome
Stiff-Person syndrome
Takayasu's arteritis
Ulcerative colitis
Vogt-Koyanagi-Harada disease
Wegener's granulomatosis

Appendix 4. Risks Associated with Atezolizumab and Guidelines for Management of Adverse Events Associated with Atezolizumab

Toxicities associated or possibly associated with atezolizumab treatment should be managed according to standard medical practice. Additional tests, such as autoimmune serology or biopsies, should be used to evaluate for a possible immunogenic etiology.

Although most Immune-mediated adverse events observed with immunomodulatory agents have been mild and self-limiting, such events should be recognized early and treated promptly to avoid potential major complications. Discontinuation of atezolizumab may not have an immediate therapeutic effect, and in severe cases, Immune-mediated toxicities may require acute management with topical corticosteroids, systemic corticosteroids, or other immunosuppressive agents.

The investigator should consider the benefit–risk balance a given patient may be experiencing prior to further administration of atezolizumab. In patients who have met the criteria for permanent discontinuation, resumption of atezolizumab may be considered if the patient is deriving benefit and has fully recovered from the Immune-mediated event. Patients can be re-challenged with atezolizumab only after approval has been documented by both the investigator (or an appropriate delegate) and the PI/Co-PI.

DOSE MODIFICATIONS

There will be no dose modifications for atezolizumab in this study.

TREATMENT INTERRUPTION

Atezolizumab treatment may be temporarily suspended in patients experiencing toxicity considered to be related to study treatment. If corticosteroids are initiated for treatment of the toxicity, they must be tapered over ≥1 month to the equivalent of ≤10 mg/day oral prednisone before atezolizumab can be resumed. If atezolizumab is withheld for >12 weeks after event

onset, the patient will be discontinued from atezolizumab. However, atezolizumab may be withheld for > 12 weeks to allow for patients to taper off corticosteroids prior to resuming treatment. Atezolizumab can be resumed after being withheld for > 12 weeks if the PI/Co-PI agrees that the patient is likely to derive clinical benefit. Atezolizumab treatment may be suspended for reasons other than toxicity (e.g., surgical procedures) with PI/Co-PI approval. The investigator and the PI/Co-PI will determine the acceptable length of treatment interruption.

MANAGEMENT GUIDELINES

PULMONARY EVENTS

Dyspnea, cough, fatigue, hypoxia, pneumonitis, and pulmonary infiltrates have been associated with the administration of atezolizumab. Patients will be assessed for pulmonary signs and symptoms throughout the study.

All pulmonary events should be thoroughly evaluated for other commonly reported etiologies such as pneumonia or other infection, lymphangitic carcinomatosis, pulmonary embolism, heart failure, chronic obstructive pulmonary disease, or pulmonary hypertension. Management guidelines for pulmonary events are provided in Table 1.

Table 1 Management Guidelines for Pulmonary Events, Including Pneumonitis

Event	Management
Pulmonary event, Grade 1	• Continue atezolizumab and monitor closely.<br>• Re-evaluate on serial imaging.<br>• Consider patient referral to pulmonary specialist.
Pulmonary event, Grade 2	• Withhold atezolizumab for up to 12 weeks after event onset. [a]<br>• Refer patient to pulmonary and infectious disease specialists and consider bronchoscopy or BAL.<br>• Initiate treatment with corticosteroids equivalent to 1–2 mg/kg/day oral prednisone.<br>• If event resolves to Grade 1 or better, resume atezolizumab. [b]<br>• If event does not resolve to Grade 1 or better while withholding atezolizumab, permanently discontinue atezolizumab and contact PI/Co-PI. [c]<br>• For recurrent events, treat as a Grade 3 or 4 event.
Pulmonary event, Grade 3 or 4	• Permanently discontinue atezolizumab and contact PI/Co-PI. [c]<br>• Bronchoscopy or BAL is recommended.<br>• Initiate treatment with corticosteroids equivalent to 1–2 mg/kg/day oral prednisone.<br>• If event does not improve within 48 hours after initiating corticosteroids, consider adding an immunosuppressive agent.<br>• If event resolves to Grade 1 or better, taper corticosteroids over ≥1 month.

BAL = bronchoscopic alveolar lavage.

[a] Atezolizumab may be withheld for a longer period of time (i.e., > 12 weeks after event onset) to allow for corticosteroids (if initiated) to be reduced to the equivalent of ≤10 mg/day oral prednisone. The acceptable length of the extended period of time must be agreed upon by the investigator and the PI/Co-PI.

[b] If corticosteroids have been initiated, they must be tapered over ≥1 month to the equivalent of ≤10 mg/day oral prednisone before atezolizumab can be resumed.

[c] Resumption of atezolizumab may be considered in patients who are deriving benefit and have fully recovered from the Immune-mediated event. Patients can be re-challenged with atezolizumab only after approval has been documented by both the investigator (or an appropriate delegate) and the PI/Co-PI.

HEPATIC EVENTS

Immune-mediated hepatitis has been associated with the administration of atezolizumab. Eligible patients must have adequate liver function, as manifested by measurements of total bilirubin and hepatic transaminases, and liver function will be monitored throughout study treatment. Management guidelines for hepatic events are provided in Table 2.

Patients with right upper-quadrant abdominal pain and/or unexplained nausea or vomiting should have liver function tests (LFTs) performed immediately and reviewed before administration of the next dose of study drug.

For patients with elevated LFTs, concurrent medication, viral hepatitis, and toxic or neoplastic etiologies should be considered and addressed, as appropriate.

Table 2 Management Guidelines for Hepatic Events

Event	Management
Hepatic event, Grade 1	• Continue atezolizumab.<br>• Monitor LFTs until values resolve to within normal limits or to baseline values.
Hepatic event, Grade 2	**All events:**<br>• Monitor LFTs more frequently until return to baseline values.<br>**Events of > 5 days' duration:**<br>• Withhold atezolizumab for up to 12 weeks after event onset. [a]<br>• Initiate treatment with corticosteroids equivalent to 1–2 mg/kg/day oral prednisone.<br>• If event resolves to Grade 1 or better, resume atezolizumab. [b]<br>• If event does not resolve to Grade 1 or better while withholding atezolizumab, permanently discontinue atezolizumab and contact PI/Co-PI. [c]

LFT = liver function tests.

[a] Atezolizumab may be withheld for a longer period of time (i.e., > 12 weeks after event onset) to allow for corticosteroids (if initiated) to be reduced to the equivalent of ≤ 10 mg/day oral prednisone. The acceptable length of the extended period of time must be agreed upon by the investigator and the PI/Co-PI.

[b] If corticosteroids have been initiated, they must be tapered over ≥ 1 month to the equivalent of ≤ 10 mg/day oral prednisone before atezolizumab can be resumed.

[c] Resumption of atezolizumab may be considered in patients who are deriving benefit and have fully recovered from the Immune-mediated event. Patients can be re-challenged with atezolizumab only after approval has been documented by both the investigator (or an appropriate delegate) and the PI/Co-PI.

Table 2 Management Guidelines for Hepatic Events (cont.)

Event	Management
Hepatic event, Grade 3 or 4	• Permanently discontinue atezolizumab and contact PI/Co-PI. [c]<br>• Consider patient referral to gastrointestinal specialist for evaluation and liver biopsy to establish etiology of hepatic injury.<br>• Initiate treatment with corticosteroids equivalent to 1–2 mg/kg/day oral prednisone.<br>• If event does not improve within 48 hours after initiating corticosteroids, consider adding an immunosuppressive agent.<br>• If event resolves to Grade 1 or better, taper corticosteroids over ≥1 month.

LFT = liver function tests.

[a] Atezolizumab may be withheld for a longer period of time (i.e., > 12 weeks after event onset) to allow for corticosteroids (if initiated) to be reduced to the equivalent of ≤ 10 mg/day oral prednisone. The acceptable length of the extended period of time must be agreed upon by the investigator and the PI/Co-PI.

[b] If corticosteroids have been initiated, they must be tapered over ≥ 1 month to the equivalent of ≤ 10 mg/day oral prednisone before atezolizumab can be resumed.

[c] Resumption of atezolizumab may be considered in patients who are deriving benefit and have fully recovered from the Immune-mediated event. Patients can be re-challenged with atezolizumab only after approval has been documented by both the investigator (or an appropriate delegate) and the PI/Co-PI.

GASTROINTESTINAL EVENTS

Immune-mediated colitis has been associated with the administration of atezolizumab. Management guidelines for diarrhea or colitis are provided in Table 3.

All events of diarrhea or colitis should be thoroughly evaluated for other more common etiologies. For events of significant duration or magnitude or associated with signs of systemic inflammation or acute-phase reactants (e.g., increased C-reactive protein, platelet count, or bandemia): Perform sigmoidoscopy (or colonoscopy, if appropriate) with colonic biopsy, with three to five specimens for standard paraffin block to check for inflammation and lymphocytic infiltrates to confirm colitis diagnosis.

Table 3 Management Guidelines for Gastrointestinal Events (Diarrhea or Colitis)

Event	Management
Diarrhea or colitis, Grade 1	• Continue atezolizumab.<br>• Initiate symptomatic treatment.<br>• Endoscopy is recommended if symptoms persist for > 7 days.<br>• Monitor closely.
Diarrhea or colitis, Grade 2	• Withhold atezolizumab for up to 12 weeks after event onset. [a]<br>• Initiate symptomatic treatment.<br>• Patient referral to GI specialist is recommended.<br>• For recurrent events or events that persist > 5 days, initiate treatment with corticosteroids equivalent to 1–2 mg/kg/day oral prednisone.<br>• If event resolves to Grade 1 or better, resume atezolizumab. [b]<br>• If event does not resolve to Grade 1 or better while withholding atezolizumab, permanently discontinue atezolizumab and contact PI/Co-PI. [c]
Diarrhea or colitis, Grade 3	• Withhold atezolizumab for up to 12 weeks after event onset. [a]<br>• Refer patient to GI specialist for evaluation and confirmatory biopsy.<br>• Initiate treatment with corticosteroids equivalent to 1–2 mg/kg/day IV methylprednisolone and convert to 1–2 mg/kg/day oral prednisone or equivalent upon improvement.<br>• If event resolves to Grade 1 or better, resume atezolizumab. [b]<br>• If event does not resolve to Grade 1 or better while withholding atezolizumab, permanently discontinue atezolizumab and contact PI/Co-PI. [c]

GI = gastrointestinal.

[a] Atezolizumab may be withheld for a longer period of time (i.e., > 12 weeks after event onset) to allow for corticosteroids (if initiated) to be reduced to the equivalent of ≤ 10 mg/day oral prednisone. The acceptable length of the extended period of time must be agreed upon by the investigator and the PI/Co-PI.

[b] If corticosteroids have been initiated, they must be tapered over ≥ 1 month to the equivalent of ≤ 10 mg/day oral prednisone before atezolizumab can be resumed.

[c] Resumption of atezolizumab may be considered in patients who are deriving benefit and have fully recovered from the Immune-mediated event. Patients can be re-challenged with atezolizumab only after approval has been documented by both the investigator (or an appropriate delegate) and the PI/Co-PI.

Table 3 Management Guidelines for Gastrointestinal Events (Diarrhea or Colitis) (cont.)

Event	Management
Diarrhea or colitis, Grade 4	• Permanently discontinue atezolizumab and contact PI/Co-PI. [c]<br>• Refer patient to GI specialist for evaluation and confirmation biopsy.<br>• Initiate treatment with corticosteroids equivalent to 1–2 mg/kg/day IV methylprednisolone and convert to 1–2 mg/kg/day oral prednisone or equivalent upon improvement.<br>• If event does not improve within 48 hours after initiating corticosteroids, consider adding an immunosuppressive agent.<br>• If event resolves to Grade 1 or better, taper corticosteroids over ≥1 month.

GI = gastrointestinal.

[a] Atezolizumab may be withheld for a longer period of time (i.e., > 12 weeks after event onset) to allow for corticosteroids (if initiated) to be reduced to the equivalent of ≤ 10 mg/day oral prednisone. The acceptable length of the extended period of time must be agreed upon by the investigator and the PI/Co-PI.

[b] If corticosteroids have been initiated, they must be tapered over ≥ 1 month to the equivalent of ≤ 10 mg/day oral prednisone before atezolizumab can be resumed.

[c] Resumption of atezolizumab may be considered in patients who are deriving benefit and have fully recovered from the Immune-mediated event. Patients can be re-challenged with atezolizumab only after approval has been documented by both the investigator (or an appropriate delegate) and the PI/Co-PI.

ENDOCRINE EVENTS

Thyroid disorders, adrenal insufficiency, diabetes mellitus, and pituitary disorders have been associated with the administration of atezolizumab. Management guidelines for endocrine events are provided in Table 4.

Patients with unexplained symptoms such as headache, fatigue, myalgias, impotence, constipation, or mental status changes should be investigated for the presence of thyroid, pituitary, or adrenal endocrinopathies. The patient should be referred to an endocrinologist if an endocrinopathy is suspected. Thyroid-stimulating hormone (TSH) and free triiodothyronine and thyroxine levels should be measured to determine whether thyroid abnormalities are present. Pituitary hormone levels and function tests (e.g., TSH, growth hormone, luteinizing hormone, follicle-stimulating hormone, testosterone, prolactin, adrenocorticotropic hormone [ACTH] levels, and ACTH stimulation test) and magnetic resonance imaging (MRI) of the brain (with detailed pituitary sections) may help to differentiate primary pituitary insufficiency from primary adrenal insufficiency.

Table 4 Management Guidelines for Endocrine Events

Event	Management
Asymptomatic hypothyroidism	• Continue atezolizumab.<br>• Initiate treatment with thyroid replacement hormone.<br>• Monitor TSH weekly.
Symptomatic hypothyroidism	• Withhold atezolizumab.<br>• Initiate treatment with thyroid replacement hormone.<br>• Monitor TSH weekly.<br>• Consider patient referral to endocrinologist.<br>• Resume atezolizumab when symptoms are controlled and thyroid function is improving.
Asymptomatic hyperthyroidism	**TSH ≥ 0.1 mU/L and < 0.5 mU/L:**<br>• Continue atezolizumab.<br>• Monitor TSH every 4 weeks.<br>**TSH < 0.1 mU/L:**<br>• Follow guidelines for symptomatic hyperthyroidism.
Symptomatic hyperthyroidism	• Withhold atezolizumab.<br>• Initiate treatment with anti-thyroid drug such as methimazole or carbimazole as needed.<br>• Consider patient referral to endocrinologist.<br>• Resume atezolizumab when symptoms are controlled and thyroid function is improving.<br>• Permanently discontinue atezolizumab and contact PI/Co-PI for life-threatening Immune-mediated hyperthyroidism. [c]

MRI = magnetic resonance imaging; TSH = thyroid-stimulating hormone.

[a] Atezolizumab may be withheld for a longer period of time (i.e., > 12 weeks after event onset) to allow for corticosteroids (if initiated) to be reduced to the equivalent of ≤ 10 mg/day oral prednisone. The acceptable length of the extended period of time must be agreed upon by the investigator and the PI/Co-PI.

[b] If corticosteroids have been initiated, they must be tapered over ≥ 1 month to the equivalent of ≤ 10 mg/day oral prednisone before atezolizumab can be resumed.

[c] Resumption of atezolizumab may be considered in patients who are deriving benefit and have fully recovered from the Immune-mediated event. Patients can be re-challenged with atezolizumab only after approval has been documented by both the investigator (or an appropriate delegate) and the PI/Co-PI.

Table 4 Management Guidelines for Endocrine Events (cont.)

Event	Management
Symptomatic adrenal insufficiency, Grade 2–4	• Withhold atezolizumab for up to 12 weeks after event onset.[a]<br>• Refer patient to endocrinologist.<br>• Perform appropriate imaging.<br>• Initiate treatment with corticosteroids equivalent to 1–2 mg/kg/day IV methylprednisolone and convert to 1–2 mg/kg/day oral prednisone or equivalent upon improvement.<br>• If event resolves to Grade 1 or better and patient is stable on replacement therapy, resume atezolizumab.[b]<br>• If event does not resolve to Grade 1 or better or patient is not stable on replacement therapy while withholding atezolizumab, permanently discontinue atezolizumab and contact PI/Co-PI.[c]
Hyperglycemia, Grade 1 or 2	• Continue atezolizumab.<br>• Investigate for diabetes. If patient has Type 1 diabetes, treat as a Grade 3 event. If patient does not have Type 1 diabetes, treat as per institutional guidelines.<br>• Monitor for glucose control.
Hyperglycemia, Grade 3 or 4	• Withhold atezolizumab.<br>• Initiate treatment with insulin.<br>• Monitor for glucose control.<br>• Resume atezolizumab when symptoms resolve and glucose levels are stable.

MRI = magnetic resonance imaging; TSH = thyroid-stimulating hormone.

[a] Atezolizumab may be withheld for a longer period of time (i.e., > 12 weeks after event onset) to allow for corticosteroids (if initiated) to be reduced to the equivalent of ≤ 10 mg/day oral prednisone. The acceptable length of the extended period of time must be agreed upon by the investigator and the PI/Co-PI.

[b] If corticosteroids have been initiated, they must be tapered over ≥ 1 month to the equivalent of ≤ 10 mg/day oral prednisone before atezolizumab can be resumed.

[c] Resumption of atezolizumab may be considered in patients who are deriving benefit and have fully recovered from the Immune-mediated event. Patients can be re-challenged with atezolizumab only after approval has been documented by both the investigator (or an appropriate delegate) and the PI/Co-PI.

Table 4 Management Guidelines for Endocrine Events (cont.)

Event	Management
Hypophysitis (pan-hypopituitarism), Grade 2 or 3	• Withhold atezolizumab for up to 12 weeks after event onset. [a]<br>• Refer patient to endocrinologist.<br>• Perform brain MRI (pituitary protocol).<br>• Initiate treatment with corticosteroids equivalent to 1–2 mg/kg/day IV methylprednisolone and convert to 1–2 mg/kg/day oral prednisone or equivalent upon improvement.<br>• Initiate hormone replacement if clinically indicated.<br>• If event resolves to Grade 1 or better, resume atezolizumab. [b]<br>• If event does not resolve to Grade 1 or better while withholding atezolizumab, permanently discontinue atezolizumab and contact PI/Co-PI. [c]<br>• For recurrent hypophysitis, treat as a Grade 4 event.
Hypophysitis (pan-hypopituitarism), Grade 4	• Permanently discontinue atezolizumab and contact PI/Co-PI. [c]<br>• Refer patient to endocrinologist.<br>• Perform brain MRI (pituitary protocol).<br>• Initiate treatment with corticosteroids equivalent to 1–2 mg/kg/day IV methylprednisolone and convert to 1–2 mg/kg/day oral prednisone or equivalent upon improvement.<br>• Initiate hormone replacement if clinically indicated.

MRI = magnetic resonance imaging; TSH = thyroid-stimulating hormone.

[a] Atezolizumab may be withheld for a longer period of time (i.e., > 12 weeks after event onset) to allow for corticosteroids (if initiated) to be reduced to the equivalent of ≤ 10 mg/day oral prednisone. The acceptable length of the extended period of time must be agreed upon by the investigator and the PI/Co-PI.

[b] If corticosteroids have been initiated, they must be tapered over ≥ 1 month to the equivalent of ≤ 10 mg/day oral prednisone before atezolizumab can be resumed.

[c] Resumption of atezolizumab may be considered in patients who are deriving benefit and have fully recovered from the Immune-mediated event. Patients can be re-challenged with atezolizumab only after approval has been documented by both the investigator (or an appropriate delegate) and the PI/Co-PI.

OCULAR EVENTS

An ophthalmologist should evaluate visual complaints (e.g., uveitis, retinal events). Management guidelines for ocular events are provided in Table 5.

Table 5 Management Guidelines for Ocular Events

Event	Management
Ocular event, Grade 1	• Continue atezolizumab.<br>• Patient referral to ophthalmologist is strongly recommended.<br>• Initiate treatment with topical corticosteroid eye drops and topical immunosuppressive therapy.<br>• If symptoms persist, treat as a Grade 2 event.
Ocular event, Grade 2	• Withhold atezolizumab for up to 12 weeks after event onset. [a]<br>• Patient referral to ophthalmologist is strongly recommended.<br>• Initiate treatment with topical corticosteroid eye drops and topical immunosuppressive therapy.<br>• If event resolves to Grade 1 or better, resume atezolizumab. [b]<br>• If event does not resolve to Grade 1 or better while withholding atezolizumab, permanently discontinue atezolizumab and contact PI/Co-PI. [c]
Ocular event, Grade 3 or 4	• Permanently discontinue atezolizumab and contact PI/Co-PI. [c]<br>• Refer patient to ophthalmologist.<br>• Initiate treatment with corticosteroids equivalent to 1–2 mg/kg/day oral prednisone.<br>• If event resolves to Grade 1 or better, taper corticosteroids over $\geq$1 month.

[a] Atezolizumab may be withheld for a longer period of time (i.e., $>$12 weeks after event onset) to allow for corticosteroids (if initiated) to be reduced to the equivalent of $\leq$10 mg/day oral prednisone. The acceptable length of the extended period of time must be agreed upon by the investigator and the PI/Co-PI.

[b] If corticosteroids have been initiated, they must be tapered over $\geq$1 month to the equivalent of $\leq$10 mg/day oral prednisone before atezolizumab can be resumed.

[c] Resumption of atezolizumab may be considered in patients who are deriving benefit and have fully recovered from the Immune-mediated event. Patients can be re-challenged with atezolizumab only after approval has been documented by both the investigator (or an appropriate delegate) and the PI/Co-PI.

IMMUNE-MEDIATED MYOCARDITIS

Immune-mediated myocarditis has been associated with the administration of atezolizumab. Immune-mediated myocarditis should be suspected in any patient presenting with signs or symptoms suggestive of myocarditis, including, but not limited to, dyspnea, chest pain, palpitations, fatigue, decreased exercise tolerance, or syncope. Immune-mediated myocarditis needs to be distinguished from myocarditis resulting from infection (commonly viral, e.g., in a patient who reports a recent history of gastrointestinal illness), ischemic events, underlying arrhythmias, exacerbation of preexisting cardiac conditions, or progression of malignancy.

All patients with possible myocarditis should be urgently evaluated by performing cardiac enzyme assessment, an ECG, a chest X-ray, an echocardiogram, and a cardiac MRI as appropriate per institutional guidelines. A cardiologist should be consulted. An endomyocardial biopsy may be considered to enable a definitive diagnosis and appropriate treatment, if clinically indicated.

Patients with signs and symptoms of myocarditis, in the absence of an identified alternate etiology, should be treated according to the guidelines in Table 6.

Table 6 Management Guidelines for Immune-mediated Myocarditis

Event	Management
Immune-mediated myocarditis, Grade 2	• Withhold atezolizumab for up to 12 weeks after event onset [a] and contact PI/Co-PI.<br>• Refer patient to cardiologist.<br>• Initiate treatment as per institutional guidelines and consider antiarrhythmic drugs, temporary pacemaker, ECMO, or VAD as appropriate.<br>• Consider treatment with corticosteroids equivalent to 1–2 mg/kg/day IV methylprednisolone and convert to 1–2 mg/kg/day oral prednisone or equivalent upon improvement.<br>• If event resolves to Grade 1 or better, resume atezolizumab. [b]<br>• If event does not resolve to Grade 1 or better while withholding atezolizumab, permanently discontinue atezolizumab and contact PI/Co-PI. [c]
Immune-mediated myocarditis, Grade 3-4	• Permanently discontinue atezolizumab and contact PI/Co-PI. [c]<br>• Refer patient to cardiologist.<br>• Initiate treatment as per institutional guidelines and consider antiarrhythmic drugs, temporary pacemaker, ECMO, or VAD as appropriate.<br>• Initiate treatment with corticosteroids equivalent to 1–2 mg/kg/day IV methylprednisolone and convert to 1–2 mg/kg/day oral prednisone or equivalent upon improvement.<br>• If event does not improve within 48 hours after initiating corticosteroids, consider adding an immunosuppressive agent.<br>• If event resolves to Grade 1 or better, taper corticosteroids over $\geq$1 month.

ECMO = extracorporeal membrane oxygenation; VAD = ventricular assist device.

[a] Atezolizumab may be withheld for a longer period of time (i.e., > 12 weeks after event onset) to allow for corticosteroids (if initiated) to be reduced to the equivalent of $\leq$10 mg/day oral prednisone. The acceptable length of the extended period of time must be agreed upon by the investigator and the PI/Co-PI.

[b] If corticosteroids have been initiated, they must be tapered over $\geq$1 month to the equivalent of $\leq$10 mg/day oral prednisone before atezolizumab can be resumed.

[c] Resumption of atezolizumab may be considered in patients who are deriving benefit and have fully recovered from the Immune-mediated event. Patients can be re-challenged with atezolizumab only after approval has been documented by both the investigator (or an appropriate delegate) and the PI/Co-PI.

INFUSION-RELATED REACTIONS

No premedication is indicated for the administration of Cycle 1 of atezolizumab. However, patients who experience an infusion-related reaction (IRR) with Cycle 1 of atezolizumab may receive premedication with antihistamines or antipyretics/analgesics (e.g., acetaminophen) for subsequent infusions. Metamizole (dipyrone) is prohibited in treating atezolizumab-associated IRRs because of its potential for causing agranulocytosis.

Guidelines for medical management of IRRs during Cycle 1 are provided in Table 7. For subsequent cycles, IRRs should be managed according to institutional guidelines.

Table 7 Management Guidelines for Infusion-Related Reactions

Event	Management
IRR, Grade 1	• Reduce infusion rate to half the rate being given at the time of event onset.<br>• After the event has resolved, the investigator should wait for 30 minutes while delivering the infusion at the reduced rate.<br>• If the infusion is tolerated at the reduced rate for 30 minutes after symptoms have resolved, the infusion rate may be increased to the original rate.
IRR, Grade 2	• Interrupt atezolizumab infusion.<br>• Administer aggressive symptomatic treatment (e.g., oral or IV antihistamine, anti-pyretic medication, glucocorticoids, epinephrine, bronchodilators, oxygen, IV fluids).<br>• After symptoms have resolved to baseline, resume infusion at half the rate being given at the time of event onset.<br>• For subsequent infusions, consider administration of oral premedication with antihistamines, anti-pyretics, and/or analgesics and monitor closely for IRRs.
IRR, Grade 3 or 4	• Stop infusion.<br>• Administer aggressive symptomatic treatment (e.g., oral or IV antihistamine, anti-pyretic medication, glucocorticoids, epinephrine, bronchodilators, oxygen, IV fluids).<br>• Permanently discontinue atezolizumab and contact PI/Co-PI. [a]

IRR = infusion-related reaction.

[a] Resumption of atezolizumab may be considered in patients who are deriving benefit and have fully recovered from the Immune-mediated event. Patients can be re-challenged with atezolizumab only after approval has been documented by both the investigator (or an appropriate delegate) and the PI/Co-PI.

PANCREATIC EVENTS

Symptoms of abdominal pain associated with elevations of amylase and lipase, suggestive of pancreatitis, have been associated with the administration of atezolizumab. The differential diagnosis of acute abdominal pain should include pancreatitis. Appropriate workup should include an evaluation for ductal obstruction, as well as serum amylase and lipase tests. Management guidelines for pancreatic events, including pancreatitis, are provided in Table 8.

Table 8 Management Guidelines for Pancreatic Events, Including Pancreatitis

Event	Management
Amylase and/or lipase elevation, Grade 2	**Amylase and/or lipase > 1.5–2.0 × ULN:**<br>• Continue atezolizumab.<br>• Monitor amylase and lipase weekly.<br>• For prolonged elevation (e.g., > 3 weeks), consider treatment with corticosteroids equivalent to 10 mg/day oral prednisone.<br>**Asymptomatic with amylase and/or lipase > 2.0–5.0 × ULN:**<br>• Treat as a Grade 3 event.
Amylase and/or lipase elevation, Grade 3 or 4	• Withhold atezolizumab for up to 12 weeks after event onset. [a]<br>• Refer patient to GI specialist.<br>• Monitor amylase and lipase every other day.<br>• If no improvement, consider treatment with corticosteroids equivalent to 1–2 mg/kg/day oral prednisone.<br>• If event resolves to Grade 1 or better, resume atezolizumab. [b]<br>• If event does not resolve to Grade 1 or better while withholding atezolizumab, permanently discontinue atezolizumab and contact PI/Co-PI. [c]<br>• For recurrent events, permanently discontinue atezolizumab and contact PI/Co-PI. [c]

GI = gastrointestinal.

[a] Atezolizumab may be withheld for a longer period of time (i.e., > 12 weeks after event onset) to allow for corticosteroids (if initiated) to be reduced to the equivalent of ≤ 10 mg/day oral prednisone. The acceptable length of the extended period of time must be agreed upon by the investigator and the PI/Co-PI.

[b] If corticosteroids have been initiated, they must be tapered over ≥ 1 month to the equivalent of ≤ 10 mg/day oral prednisone before atezolizumab can be resumed.

[c] Resumption of atezolizumab may be considered in patients who are deriving benefit and have fully recovered from the Immune-mediated event. Patients can be re-challenged with atezolizumab only after approval has been documented by both the investigator (or an appropriate delegate) and the PI/Co-PI.

Table 8 Management Guidelines for Pancreatic Events, Including Pancreatitis (cont.)

Event	Management
Immune-mediated pancreatitis, Grade 2 or 3	• Withhold atezolizumab for up to 12 weeks after event onset. [a]<br>• Refer patient to GI specialist.<br>• Initiate treatment with corticosteroids equivalent to 1–2 mg/kg/day IV methylprednisolone and convert to 1–2 mg/kg/day oral prednisone or equivalent upon improvement.<br>• If event resolves to Grade 1 or better, resume atezolizumab. [b]<br>• If event does not resolve to Grade 1 or better while withholding atezolizumab, permanently discontinue atezolizumab and contact PI/Co-PI. [c]<br>• For recurrent events, permanently discontinue atezolizumab and contact PI/Co-PI. [c]
Immune-mediated pancreatitis, Grade 4	• Permanently discontinue atezolizumab and contact PI/Co-PI. [c]<br>• Refer patient to GI specialist.<br>• Initiate treatment with corticosteroids equivalent to 1–2 mg/kg/day IV methylprednisolone and convert to 1–2 mg/kg/day oral prednisone or equivalent upon improvement.<br>• If event does not improve within 48 hours after initiating corticosteroids, consider adding an immunosuppressive agent.<br>• If event resolves to Grade 1 or better, taper corticosteroids over ≥1 month.

GI = gastrointestinal.

[a] Atezolizumab may be withheld for a longer period of time (i.e., > 12 weeks after event onset) to allow for corticosteroids (if initiated) to be reduced to the equivalent of ≤10 mg/day oral prednisone. The acceptable length of the extended period of time must be agreed upon by the investigator and the PI/Co-PI.

[b] If corticosteroids have been initiated, they must be tapered over ≥1 month to the equivalent of ≤10 mg/day oral prednisone before atezolizumab can be resumed.

[c] Resumption of atezolizumab may be considered in patients who are deriving benefit and have fully recovered from the Immune-mediated event. Patients can be re-challenged with atezolizumab only after approval has been documented by both the investigator (or an appropriate delegate) and the PI/Co-PI.

DERMATOLOGIC EVENTS

Treatment-emergent rash has been associated with atezolizumab. The majority of cases of rash were mild in severity and self limited, with or without pruritus. A dermatologist should evaluate persistent and/or severe rash or pruritus. A biopsy should be considered unless contraindicated. Management guidelines for dermatologic events are provided in Table 9.

Table 9 Management Guidelines for Dermatologic Events

Event	Management
Dermatologic event, Grade 1	• Continue atezolizumab.<br>• Consider treatment with topical corticosteroids and/or other symptomatic therapy (e.g., antihistamines).
Dermatologic event, Grade 2	• Continue atezolizumab.<br>• Consider patient referral to dermatologist.<br>• Initiate treatment with topical corticosteroids.<br>• Consider treatment with higher-potency topical corticosteroids if event does not improve.
Dermatologic event, Grade 3	• Withhold atezolizumab for up to 12 weeks after event onset. [a]<br>• Refer patient to dermatologist.<br>• Initiate treatment with corticosteroids equivalent to 10 mg/day oral prednisone, increasing dose to 1–2 mg/kg/day if event does not improve within 48–72 hours.<br>• If event resolves to Grade 1 or better, resume atezolizumab. [b]<br>• If event does not resolve to Grade 1 or better while withholding atezolizumab, permanently discontinue atezolizumab and contact PI/Co-PI. [c]
Dermatologic event, Grade 4	• Permanently discontinue atezolizumab and contact PI/Co-PI. [c]

[a] Atezolizumab may be withheld for a longer period of time (i.e., $>$12 weeks after event onset) to allow for corticosteroids (if initiated) to be reduced to the equivalent of $\leq$10 mg/day oral prednisone. The acceptable length of the extended period of time must be agreed upon by the investigator and the PI/Co-PI.

[b] If corticosteroids have been initiated, they must be tapered over $\geq$1 month to the equivalent of $\leq$10 mg/day oral prednisone before atezolizumab can be resumed.

[c] Resumption of atezolizumab may be considered in patients who are deriving benefit and have fully recovered from the Immune-mediated event. Patients can be re-challenged with atezolizumab only after approval has been documented by both the investigator (or an appropriate delegate) and the PI/Co-PI.

NEUROLOGIC DISORDERS

Myasthenia gravis and Guillain-Barré syndrome have been observed with single-agent atezolizumab. Patients may present with signs and symptoms of sensory and/or motor neuropathy. Diagnostic workup is essential for an accurate characterization to differentiate

between alternative etiologies. Management guidelines for neurologic disorders are provided in Table 10.

Table 10 Management Guidelines for Neurologic Disorders

Event	Management
Immune-mediated neuropathy, Grade 1	• Continue atezolizumab.<br>• Investigate etiology.
Immune-mediated neuropathy, Grade 2	• Withhold atezolizumab for up to 12 weeks after event onset. [a]<br>• Investigate etiology.<br>• Initiate treatment as per institutional guidelines.<br>• If event resolves to Grade 1 or better, resume atezolizumab. [b]<br>• If event does not resolve to Grade 1 or better while withholding atezolizumab, permanently discontinue atezolizumab and contact PI/Co-PI. [c]
Immune-mediated neuropathy, Grade 3 or 4	• Permanently discontinue atezolizumab and contact PI/Co-PI. [c]<br>• Initiate treatment as per institutional guidelines.
Myasthenia gravis and Guillain-Barré syndrome (any grade)	• Permanently discontinue atezolizumab and contact PI/Co-PI. [c]<br>• Refer patient to neurologist.<br>• Initiate treatment as per institutional guidelines.<br>• Consider initiation of corticosteroids equivalent to 1–2 mg/kg/day oral or IV prednisone.

[a] Atezolizumab may be withheld for a longer period of time (i.e., $>$12 weeks after event onset) to allow for corticosteroids (if initiated) to be reduced to the equivalent of $\leq$10 mg/day oral prednisone. The acceptable length of the extended period of time must be agreed upon by the investigator and the PI/Co-PI.

[b] If corticosteroids have been initiated, they must be tapered over $\geq$1 month to the equivalent of $\leq$10 mg/day oral prednisone before atezolizumab can be resumed.

[c] Resumption of atezolizumab may be considered in patients who are deriving benefit and have fully recovered from the Immune-mediated event. Patients can be re-challenged with atezolizumab only after approval has been documented by both the investigator (or an appropriate delegate) and the PI/Co-PI.

IMMUNE-MEDIATED MENINGOENCEPHALITIS

Immune-mediated meningoencephalitis is an identified risk associated with the administration of atezolizumab. Immune-mediated meningoencephalitis should be suspected in any patient presenting with signs or symptoms suggestive of meningitis or encephalitis, including, but not limited to, headache, neck pain, confusion, seizure, motor or sensory dysfunction, and altered or depressed level of consciousness. Encephalopathy from metabolic or electrolyte imbalances needs to be distinguished from potential meningoencephalitis resulting from infection (bacterial, viral, or fungal) or progression of malignancy, or secondary to a paraneoplastic process.

All patients being considered for meningoencephalitis should be urgently evaluated with a CT scan and/or MRI scan of the brain to evaluate for metastasis, inflammation, or edema. If deemed safe by the treating physician, a lumbar puncture should be performed and a neurologist should be consulted.

Patients with signs and symptoms of meningoencephalitis, in the absence of an identified alternate etiology, should be treated according to the guidelines in Table 11.

Table 11 Management Guidelines for Immune-mediated Meningoencephalitis

Event	Management
Immune-mediated meningoencephalitis, all grades	• Permanently discontinue atezolizumab and contact PI/Co-PI. [a]<br>• Refer patient to neurologist.<br>• Initiate treatment with corticosteroids equivalent to 1–2 mg/kg/day IV methylprednisolone and convert to 1–2 mg/kg/day oral prednisone or equivalent upon improvement.<br>• If event does not improve within 48 hours after initiating corticosteroids, consider adding an immunosuppressive agent.<br>• If event resolves to Grade 1 or better, taper corticosteroids over ≥1 month.

[a] Resumption of atezolizumab may be considered in patients who are deriving benefit and have fully recovered from the Immune-mediated event. Patients can be re-challenged with atezolizumab only after approval has been documented by both the investigator (or an appropriate delegate) and the PI/Co-PI.

RENAL EVENTS

Immune-mediated nephritis has been associated with the administration of atezolizumab. Eligible patients must have adequate renal function, and renal function, including serum creatinine, should be monitored throughout study treatment. Patients with abnormal renal function should be evaluated and treated for other more common etiologies (including prerenal and postrenal causes, and concomitant medications such as non-steroidal anti-inflammatory drugs). Refer the patient to a renal specialist if clinically indicated. A renal biopsy may be required to enable a definitive diagnosis and appropriate treatment.

Patients with signs and symptoms of nephritis, in the absence of an identified alternate etiology, should be treated according to the guidelines in Table 12.

Table 12 Management Guidelines for Renal Events

Event	Management
Renal event, Grade 1	• Continue atezolizumab.<br>• Monitor kidney function, including creatinine, closely until values resolve to within normal limits or to baseline values.

Renal event, Grade 2	• Withhold atezolizumab for up to 12 weeks after event onset. [a]<br>• Refer patient to renal specialist.<br>• Initiate treatment with corticosteroids equivalent to 1–2 mg/kg/day oral prednisone.<br>• If event resolves to Grade 1 or better, resume atezolizumab. [b]<br>• If event does not resolve to Grade 1 or better while withholding atezolizumab, permanently discontinue atezolizumab and contact PI/Co-PI. [c]
Renal event, Grade 3 or 4	• Permanently discontinue atezolizumab and contact PI/Co-PI.<br>• Refer patient to renal specialist and consider renal biopsy.<br>• Initiate treatment with corticosteroids equivalent to 1–2 mg/kg/day oral prednisone.<br>• If event does not improve within 48 hours after initiating corticosteroids, consider adding an immunosuppressive agent.<br>• If event resolves to Grade 1 or better, taper corticosteroids over ≥1 month.

[a] Atezolizumab may be withheld for a longer period of time (i.e., >12 weeks after event onset) to allow for corticosteroids (if initiated) to be reduced to the equivalent of ≤10 mg/day oral prednisone. The acceptable length of the extended period of time must be agreed upon by the investigator and the PI/Co-PI.

[b] If corticosteroids have been initiated, they must be tapered over ≥1 month to the equivalent of ≤10 mg/day oral prednisone before atezolizumab can be resumed.

[c] Resumption of atezolizumab may be considered in patients who are deriving benefit and have fully recovered from the Immune-mediated event. Patients can be re-challenged with atezolizumab only after approval has been documented by both the investigator (or an appropriate delegate) and the PI/Co-PI.

IMMUNE-MEDIATED MYOSITIS

Immune-mediated myositis has been associated with the administration of atezolizumab. Myositis or inflammatory myopathies are a group of disorders sharing the common feature of inflammatory muscle injury; dermatomyositis and polymyositis are among the most common disorders. Initial diagnosis is based on clinical (muscle weakness, muscle pain, skin rash in dermatomyositis), biochemical (serum creatine kinase increase), and imaging (electromyography/MRI) features, and is confirmed with a muscle biopsy.

Patients with signs and symptoms of myositis, in the absence of an identified alternate etiology, should be treated according to the guidelines in Table 13.

Table 13 Management Guidelines for Immune-mediated Myositis

Event	Management
Immune-mediated myositis, Grade 1	• Continue atezolizumab.<br>• Refer patient to rheumatologist or neurologist.<br>• Initiate treatment as per institutional guidelines.

Immune-mediated myositis, Grade 2	• Withhold atezolizumab for up to 12 weeks after event onset [a] and contact PI/Co-PI.<br>• Refer patient to rheumatologist or neurologist.<br>• Initiate treatment as per institutional guidelines.<br>• Consider treatment with corticosteroids equivalent to 1–2 mg/kg/day IV methylprednisolone and convert to 1–2 mg/kg/day oral prednisone or equivalent upon improvement.<br>• If corticosteroids are initiated and event does not improve within 48 hours after initiating corticosteroids, consider adding an immunosuppressive agent.<br>• If event resolves to Grade 1 or better, resume atezolizumab. [b]<br>• If event does not resolve to Grade 1 or better while withholding atezolizumab, permanently discontinue atezolizumab and contact PI/Co-PI. [c]

[a] Atezolizumab may be withheld for a longer period of time (i.e., > 12 weeks after event onset) to allow for corticosteroids (if initiated) to be reduced to the equivalent of ≤ 10 mg/day oral prednisone. The acceptable length of the extended period of time must be agreed upon by the investigator and the PI/Co-PI.

[b] If corticosteroids have been initiated, they must be tapered over ≥ 1 month to the equivalent of ≤ 10 mg/day oral prednisone before atezolizumab can be resumed.

[c] Resumption of atezolizumab may be considered in patients who are deriving benefit and have fully recovered from the Immune-mediated event. Patients can be re-challenged with atezolizumab only after approval has been documented by both the investigator (or an appropriate delegate) and the PI/Co-PI.

Table 13 Management Guidelines for Immune-mediated Myositis (cont.)

Immune-mediated myositis, Grade 3	• Withhold atezolizumab for up to 12 weeks after event onset [a] and contact PI/Co-PI.<br>• Refer patient to rheumatologist or neurologist.<br>• Initiate treatment as per institutional guidelines. Respiratory support may be required in more severe cases.<br>• Initiate treatment with corticosteroids equivalent to 1–2 mg/kg/day IV methylprednisolone, or higher-dose bolus if patient is severely compromised (e.g., cardiac or respiratory symptoms, dysphagia, or weakness that severely limits mobility); convert to 1–2 mg/kg/day oral prednisone or equivalent upon improvement.<br>• If event does not improve within 48 hours after initiating corticosteroids, consider adding an immunosuppressive agent.<br>• If event resolves to Grade 1 or better, resume atezolizumab. [b]<br>• If event does not resolve to Grade 1 or better while withholding atezolizumab, permanently discontinue atezolizumab and contact PI/Co-PI. [c]<br>• For recurrent events, treat as a Grade 4 event.
Immune-mediated myositis, Grade 4	• Permanently discontinue atezolizumab and contact PI/Co-PI. [c]<br>• Refer patient to rheumatologist or neurologist.<br>• Initiate treatment as per institutional guidelines. Respiratory support may be required in more severe cases.<br>• Initiate treatment with corticosteroids equivalent to 1–2 mg/kg/day IV methylprednisolone, or higher-dose bolus if patient is severely compromised (e.g., cardiac or respiratory symptoms, dysphagia, or weakness that severely limits mobility); convert to 1–2 mg/kg/day oral prednisone or equivalent upon improvement.<br>• If event does not improve within 48 hours after initiating corticosteroids, consider adding an immunosuppressive agent.<br>• If event resolves to Grade 1 or better, taper corticosteroids over ≥ 1 month.

[a] Atezolizumab may be withheld for a longer period of time (i.e., >12 weeks after event onset) to allow for corticosteroids (if initiated) to be reduced to the equivalent of ≤ 10 mg/day oral prednisone. The acceptable length of the extended period of time must be agreed upon by the investigator and the PI/Co-PI.

[b] If corticosteroids have been initiated, they must be tapered over ≥ 1 month to the equivalent of ≤ 10 mg/day oral prednisone before atezolizumab can be resumed.

[c] Resumption of atezolizumab may be considered in patients who are deriving benefit and have fully recovered from the Immune-mediated event. Patients can be re-challenged with atezolizumab only after approval has been documented by both the investigator (or an appropriate delegate) and the PI/Co-PI.

REFERENCES

Hejblum G, Lambotte O, Galicier L, et al. A web-based Delphi study for eliciting helpful criteria in the positive diagnosis of hemophagocytic syndrome in adult patients. PLoS ONE 2014;9(4):e94024. doi: 10.1371/journal.pone.0094024.

Henter JI, Horne A, Aricó M, et al. HLH-2004: diagnostic and therapeutic guidelines for hemophagocytic lymphohistiocytosis. Pediatr Blood Cancer 2007;48:124–31.

Appendix 5. Biomarker Management Plan (BMP) for imCORE ISRs

Please see attached document.

Appendix 6. Intravenous Administration Volumes and Time of RO7198457 in Dose Modification

**Intravenous Administration Volumes of RO7198457 Study Drug (RO7198457-A and RO7198457-B)**		
Dose (mcg)	Total volume of RO7198457-A dose solution to administer to patient (mL)	Total volume of RO7198457-B dose solution to administer to patient (mL)
**15**	**0.75 (7.5 mcg)**	**0.75 (7.5 mcg)**
**25**	**1.25 (12.5 mcg)**	**1.25 (12.5 mcg)**
If a dose different from those provided in table above is planned, calculate the total dose solution volume as follows: Total Dose Solution volume mL = Dose (mcg) / (10 mcg/ml)<br><br>Divide the total dose solution volume (mL) by two to calculate the volume of RO7198457-A dose solution and RO7198457-B dose solution.		

RO7198457 Administration using a manual IV Push in Dose Modification

The infusion time for doses delivered via IV push is calculated based on a 2.5 mL/minute (25 mcg/minute) infusion rate (although the infusion may be slowed or interrupted for patients who experience infusion-associated symptoms).

The infusion volumes and times are specified in the table below for RO7198457-A and RO7198457-B.

The infusion line should be primed with normal saline (0.9% Sodium Chloride Injection). Administer the amount of RO7198457-A indicated over the time specified in Table below. To ensure the complete dose of RO7198457-A is administered, flush the infusion line with normal saline at the same infusion rate as the dose solution.

After a 5-minute wait period, administer the amount of RO7198457-B indicated over the time specified in the table below. To ensure the complete dose of RO7198457-B is administered, flush the infusion line with normal saline at the same infusion rate as the dose solution.

Intravenous Infusion Volume and Time of RO7198457 Study Drug (RO7198457-A and RO7198457-B)

Dose (mcg)	Total volume of RO7198457-A dose solution to administer to patient (mL)	Administration time of RO7198457 A dose solution (min)	Wait Period	Total volume of RO7198457-B dose solution to administer to patient (mL)	Administration time of RO7198457 B dose solution (min)
**15**	**0.75 (7.5mcg)**	**0.3 (18 sec)**	**5 minutes**	**0.75 (7.5mcg)**	**0.3 (18 sec)**
**25**	**1.25 (12.5 mcg)**	**0.5 (30 sec)**		**1.25 (12.5 mcg)**	**0.5 (30 sec)**

<u>Appendix 7</u>. Safety Reporting of COVID-19 in Study Patients (Refer to Appendix 7)

The purpose for this guidance is to provide direction to Investigators/Study Coordinators about reporting events experienced by trial subjects diagnosed with coronavirus disease (COVID-19) or who may be at risk for developing COVID-19 due to recent travel or possible exposure to someone with COVID-19. Please refer to the Appendix 7 document for the most up to date information.
