# Supplementary material for: Personalized RNA neoantigen vaccines stimulate T cells in pancreatic cancer
Source: Nature. 2023 May 10;618(7963):144–50. doi: 10.1038/s41586-023-06063-y (PMC10171177; doi:10.1038/s41586-023-06063-y)
Supplement: Supplementary file 2 — Reporting Summary [file 41586_2023_6063_MOESM2_ESM.pdf]

nature portfolio

Corresponding author(s): Vinod Balachandran

Last updated by author(s): Mar 23, 2023

Reporting Summary

Nature Portfolio wishes to improve the reproducibility of the work that we publish. This form provides structure for consistency and transparency in reporting. For further information on Nature Portfolio policies, see our Editorial Policies and the Editorial Policy Checklist.

Statistics

For all statistical analyses, confirm that the following items are present in the figure legend, table legend, main text, or Methods section.

n/a	Confirmed	
☐	☒	The exact sample size (*n*) for each experimental group/condition, given as a discrete number and unit of measurement
☐	☒	A statement on whether measurements were taken from distinct samples or whether the same sample was measured repeatedly
☐	☒	The statistical test(s) used AND whether they are one- or two-sided<br>*Only common tests should be described solely by name; describe more complex techniques in the Methods section.*
☐	☒	A description of all covariates tested
☐	☒	A description of any assumptions or corrections, such as tests of normality and adjustment for multiple comparisons
☐	☒	A full description of the statistical parameters including central tendency (e.g. means) or other basic estimates (e.g. regression coefficient) AND variation (e.g. standard deviation) or associated estimates of uncertainty (e.g. confidence intervals)
☐	☒	For null hypothesis testing, the test statistic (e.g. *F*, *t*, *r*) with confidence intervals, effect sizes, degrees of freedom and *P* value noted<br>*Give P values as exact values whenever suitable.*
☐	☒	For Bayesian analysis, information on the choice of priors and Markov chain Monte Carlo settings
☒	☐	For hierarchical and complex designs, identification of the appropriate level for tests and full reporting of outcomes
☒	☐	Estimates of effect sizes (e.g. Cohen's *d*, Pearson's *r*), indicating how they were calculated

Our web collection on statistics for biologists contains articles on many of the points above.

Software and code

Policy information about availability of computer code

Data collection	Flow cytometric data were collected using FACSDiva (BD Biosciences, version 8.0.1).<br><br>Whole exome sequence reads of tumor-normal paired samples of patients were aligned to the reference human genome (hg19) using the Burrows-Wheeler Alignment tool (bwa mem v0.7.17) and samtools (v1.6). Duplicates were marked with picard-2.11.0 MarkDuplicates (http://broadinstitute.github.io/picard). Indel realignments were done with the Genome Analysis toolkit (GenomeAnalysisTK-3.8-1-0-gf15c1c3ef) RealignerTargetCreator and IndelRealigner (ref #60) using 1000 genome phase1 indel (1000G_phase1.indels.b37.vcf) and Mills indel calls (Mills_and_1000G_gold_standard.indels.b37.vcf) as references. MuTect 1.1.7 and Strelka 1.0.15 were used to call SNVs and indels on pre-processed sequencing data. For the MuTect calls, dbSNP 138 and CosmicCodingMuts.vcf version 86 (ref #61) were used as reference files. Unbiased normal/tumor read counts for each SNV and indel call were then assigned with the bam-readcount software 0.8.0-unstable-6-963acab-dirty (commit 963acab-dirty)) (https://github.com/genome/bam-readcount). In addition to point mutations, we called tumor somatic copy number variations in tumor using FACETS (https://github.com/mskcc/facets).<br><br>Code used to infer clonality is available at https://github.com/genome/bam-readcount, https://github.com/mskcc/facets, and https://github.com/morrislab/phylowgs, with details outlined in the Methods.<br><br>Code used to construct and apply the Neoantigen Quality model is available at GitHub https://github.com/LukszaLab/NeoantigenEditing.<br><br>Code used to track T cell clones is available at https://github.com/zsethna/TREP.
Data analysis	All analyses were performed using GraphPad Prism (version 9.3.1) or Python (version 3.4).

Data analysis

Exome sequencing, RNA sequencing, identification of neoantigens, and neoantigen selection strategies for vaccines have been previously described (Ref # 16).

In vivo T cell clonal expansion was determined as described in the methods with a Fisher exact test implemented from scipy.stats.fisher_exact (Python 3.4).

In vitro T cell activation was determined as described in the methods with a one-tailed binomial test P value (implementing the scipy.stats.binom_test) with a 0.2 threshold (significance with respect to at least 20% of a clone being CD107a+ as opposed to CD107a-), with adjusted P values using a Bonferroni correction and significance determined at a p_adj<0.001 threshold (Python 3.4).

Tumor clones were reconstructed with the PhyloWGS algorithm (https://github.com/morrislab/phylowgs). For clonality, SNVs and indels were called using MuTect 1.1.7 and Strelka 1.0.15 (http://software.broadinstitute.org/gatk/download/mutect). For neoantigen quality assessment, wild-type and mutant genomic sequences corresponding to coding mutations were translated to an amino acid sequence consistent with the GRCh37 reference genome (GRCh37.75) using snpEff.v4.3t software. Predictions of MHC class-I binding for both the wild type and mutant peptides were estimated using the NetMHC 3.4 software.

For manuscripts utilizing custom algorithms or software that are central to the research but not yet described in published literature, software must be made available to editors and reviewers. We strongly encourage code deposition in a community repository (e.g. GitHub). See the Nature Portfolio guidelines for submitting code & software for further information.

Data

Policy information about availability of data

All manuscripts must include a data availability statement. This statement should provide the following information, where applicable:

- Accession codes, unique identifiers, or web links for publicly available datasets
- A description of any restrictions on data availability
- For clinical datasets or third party data, please ensure that the statement adheres to our policy

The IRB-approved clinical protocol is provided in the Supplementary Materials. All single-cell sequencing data are available at GEO (accession number GSE222011). All experimental source data are provided. De-identified individual participant data reported in the manuscript will be shared under data use agreements upon reasonable request. Requests must be made to balachav@mskcc.org.

Databases/datasets used in this study:
TCR Vβ sequencing – IMGT database (www.imgt.org)
Neoantigen Quality – Immune Epitope Data Base (IEDB; www.iedb.org)

Human research participants

Policy information about studies involving human research participants and Sex and Gender in Research.

Reporting on sex and gender

We report sex of all patients enrolled in the trial in Extended Data Figure 1A, and Extended Data Figure 1B.

Population characteristics

All patients had clinical characteristics typical of resectable PDAC patients. Detailed patient characteristics are provided in Extended Data Figure 1A, and Extended Data Figure 1B.

Recruitment

Full details of inclusion/enrollment and recruitment are provided in the clinical trial protocol. All subjects meeting the eligibility requirements were considered for enrollment regardless of sex, race, or religion. Subjects will be accrued from the HPB Service, GI Oncology Service, and Gastric/Mixed Tumor Service, and from both the MSK Department of Surgery and the MSK Department of Medicine.

We enrolled patients of ECOG performance status 0-1 with single, radiographically suspicious, surgically resectable PDACs, no distant metastases, and ≥5 neoantigens. We excluded patients with metastatic, borderline, or locally unresectable PDACs, and patients who received neoadjuvant therapy. After surgery, we included patients with pathologically confirmed PDAC with R0/R1 margins. Additional eligibility criteria and ethical study conduct information are in the protocol (Supplementary File 1). We targeted to accrue 20 evaluable patients.

Potential bias: Our study successfully enrolled only Caucasian patients. This potential bias and the implications for future studies is discussed in the manuscript

Ethics oversight

We conducted the study in accordance with the Declaration of Helsinki and good clinical practice guidelines. The study was approved by the institutional review board (IRB) at Memorial Sloan Kettering Cancer Center (MSK), the United States Federal Drug Administration (FDA), and was registered on clinicaltrials.gov (NCT04161755). All participants provided written informed consent.

Note that full information on the approval of the study protocol must also be provided in the manuscript.

Field-specific reporting

Please select the one below that is the best fit for your research. If you are not sure, read the appropriate sections before making your selection.

☒ Life sciences ☐ Behavioural & social sciences ☐ Ecological, evolutionary & environmental sciences

For a reference copy of the document with all sections, see nature.com/documents/nr-reporting-summary-flat.pdf

Life sciences study design

All studies must disclose on these points even when the disclosure is negative.

Sample size	We targeted to accrue a total of 20 evaluable patients based on estimated sample size needed to evaluate our primary endpoint of safety. Additional detailed sample size estimation and statistical calculations are in the manuscript (Table 1) and protocol (Supplementary File 1).
Data exclusions	No data were excluded.
Replication	All findings were reproducible. All experimental replicates, performed as independent experiments in individual patient samples, are outlined in the figures and figure legends. Technical replicates are also indicated in the figure legends when appropriate.
Randomization	Randomization is not applicable to this single-arm, phase-I clinical trial.
Blinding	Investigators were blinded to survival events during immunologic response determination. Other experiments: no blinding.

Reporting for specific materials, systems and methods

We require information from authors about some types of materials, experimental systems and methods used in many studies. Here, indicate whether each material, system or method listed is relevant to your study. If you are not sure if a list item applies to your research, read the appropriate section before selecting a response.

Materials & experimental systems

n/a	Involved in the study	
☐	☒	Antibodies
☐	☒	Eukaryotic cell lines
☒	☐	Palaeontology and archaeology
☒	☐	Animals and other organisms
☐	☒	Clinical data
☒	☐	Dual use research of concern

Methods

n/a	Involved in the study	
☒	☐	ChIP-seq
☐	☒	Flow cytometry
☒	☐	MRI-based neuroimaging

Antibodies

Antibodies used

CD62L - clone DREG-56, BV510 (Biolegend Cat# 304844); 2 µl/sample
CD56 - clone HCD56, BV605 (Biolegend Cat# 318334); 2 µl/sample
CD4 - clone OKT4, BV650 (Biolegend Cat# 317436); 2 µl/sample
CD19 - clone HIB19, BV711 (Biolegend Cat# 302246); 2 µl/sample
FoxP3 - clone 206D, PE (Biolegend Cat# 320108); 5 µl/sample
CD3 - clone SK-7, PE-Cy7 (Biolegend Cat# 344816); 2µl/sample
CD8 - clone SK1, FITC (Biolegend Cat# 344704); 2 µl/sample
CD8 - clone SK1, Alexa Fluor 700 (Biolegend Cat# 344724); 2 µl/sample
CD45RA -clone HI100, APC (Biolegend Cat# 304112); 2µl/sample
CD45 - clone 2D1, Alexa Fluor 700 (Biolegend Cat# 368514); 5 µl/sample
CD39 - clone A1, BV421 (Biolegend Cat# 328214); 5 µl/sample
LAG-3 - clone 11C3C65, PerCP-Cy5.5 (Biolegend Cat# 369312); 5 µl/sample
CD366 - clone F38-2E2, APC-Cy7 (Biolegend Cat# 345026); 5µl/sample
CD11c - clone S-HCL-3, BV421 (Biolegend Cat# 371512); 2 µl/sample
HLA-DR - clone L243, BV785 (Biolegend Cat# 307642); 5 µl/sample
CD14 - clone HCD14, PE (Biolegend Cat# 325606); 2 µl/sample
CD11b - clone ICRF44, APC (Biolegend Cat# 301310); 5 µl/sample
IFNγ - clone 4S.B3, BV421 (Biolegend Cat# 502532); 5 µl/sample
mTRB - clone H57-597, PE-Cy5 (Biolegend Cat# 109210); 0.5 µl/sample
CD137 - clone 4B4-1, PE (Biolegend Cat# 309808); 3µl/sample
HLA-A,B,C - clone W6/23, APC) (Biolegend Cat# 311409); 5 µl/sample
PD-1 - clone EH12.1, BV786 (BD Biosciences Cat# 563789); 5 µl/sample
TNFα - clone MAb11, APC (BD Biosciences Cat# 554514); 2.5 µl/sample

CD107a - clone H4A3, PE (BD Biosciences Cat# 555801); 20 µl/ sample
CD56 - clone NCAM16.2, BV786 (BD Biosciences Cat# 564058); 5 µl/sample
Ki-67 - clone SolA15, PE-Cy5 (ThermoFisher Scientific Cat# 15-5698-82); 2.5 µl/sample
anti-Rabbit HRP secondary antibody (Leica Biosystems, Catalog # DS9800); the antibody is ready-to-use, so no dilution needed

Validation

All antibodies were validated by the manufacturer and used per their instructions. In our experiments, isotype and/or FMO control samples were included. Additional information on validation can be found on the manufacturer's websites, below.
CD62L - clone DREG-56, BV510 (Biolegend Cat# 304844): https://www.biolegend.com/en-us/products/brilliant-violet-510-anti-human-cd62l-antibody-13426?GroupID=BLG10034
CD56 - clone HCD56, BV605 (Biolegend Cat# 318334): https://www.biolegend.com/en-us/products/brilliant-violet-605-anti-human-cd56-ncam-antibody-7668
CD4 - clone OKT4, BV650 (Biolegend Cat# 317436): https://www.biolegend.com/en-us/products/brilliant-violet-650-anti-human-cd4-antibody-7786
CD19 - clone HIB19, BV711 (Biolegend Cat# 302246): https://www.biolegend.com/en-us/products/brilliant-violet-711-anti-human-cd19-antibody-8519
FoxP3 - clone 206D, PE (Biolegend Cat# 320108): https://www.biolegend.com/en-us/products/pe-anti-human-foxp3-antibody-3178
CD3 - clone SK-7, PE-Cy7 (Biolegend Cat# 344816): https://www.biolegend.com/en-us/products/pe-cyanine7-anti-human-cd3-antibody-6934
CD8 - clone SK1, FITC (Biolegend Cat# 344704): https://www.biolegend.com/en-us/products/fitc-anti-human-cd8-antibody-6149
CD8 - clone SK1, Alexa Fluor 700 (Biolegend Cat# 344724): https://www.biolegend.com/en-us/products/fitc-anti-human-cd8-antibody-6149
CD45RA -clone HI100, APC (Biolegend Cat# 304112): https://www.biolegend.com/en-us/products/apc-anti-human-cd45ra-antibody-684
CD45 - clone 2D1, Alexa Fluor 700 (Biolegend Cat# 368514): https://www.biolegend.com/en-us/products/alexa-fluor-700-anti-human-cd45-antibody-12399
CD39 - clone A1, BV421 (Biolegend Cat# 328214): https://www.biolegend.com/en-us/products/brilliant-violet-421-anti-human-cd39-antibody-7204
LAG-3 - clone 11C3C65, PerCP-Cy5.5 (Biolegend Cat# 369312): https://www.biolegend.com/en-us/products/percp-cyanine5-5-anti-human-cd223-lag-3-antibody-13552
CD366 - clone F38-2E2, APC-Cy7 (Biolegend Cat# 345026): https://www.biolegend.com/en-us/products/apc-cyanine7-anti-human-cd366-tim-3-antibody-11928
CD11c - clone S-HCL-3, BV421 (Biolegend Cat# 371512): https://www.biolegend.com/en-us/products/brilliant-violet-421-anti-human-cd11c-antibody-14048
HLA-DR - clone L243, BV785 (Biolegend Cat# 307642): https://www.biolegend.com/en-us/products/brilliant-violet-785-anti-human-hla-dr-antibody-7975
CD14 - clone HCD14, PE (Biolegend Cat# 325606): https://www.biolegend.com/en-us/products/pe-anti-human-cd14-antibody-3952
CD11b - clone ICRF44, APC (Biolegend Cat# 301310): https://www.biolegend.com/en-us/products/apc-anti-human-cd11b-antibody-765
IFNγ - clone 4S.B3, BV421 (Biolegend Cat# 502532): https://www.biolegend.com/en-us/products/brilliant-violet-421-anti-human-ifn-gamma-antibody-7189
mTRB - clone H57-597, PE-Cy5 (Biolegend Cat# 109210): https://www.biolegend.com/en-us/products/pe-cyanine5-anti-mouse-tcr-beta-chain-antibody-273
CD137 - clone 4B4-1, PE (Biolegend Cat# 309808): https://www.biolegend.com/en-us/products/pe-cyanine5-anti-human-cd137-4-1bb-antibody-3909
HLA-A,B,C - clone W6/23, APC) (Biolegend Cat# 311409): https://www.biolegend.com/en-us/products/apc-anti-human-hla-a-b-c-antibody-1870
PD-1 - clone EH12.1, BV786 (BD Biosciences Cat# 563789): https://www.bdbiosciences.com/en-us/products/reagents/flow-cytometry-reagents/research-reagents/single-color-antibodies-ruo/BV786-Mouse-Anti-Human-CD279-(PD-1).563789
TNFα - clone MAb11, APC (BD Biosciences Cat# 554514): https://www.bdbiosciences.com/en-us/products/reagents/flow-cytometry-reagents/research-reagents/single-color-antibodies-ruo/apc-mouse-anti-human-tnf.554514
CD107a - clone H4A3, PE (BD Biosciences Cat# 555801): https://www.bdbiosciences.com/en-us/products/reagents/flow-cytometry-reagents/research-reagents/single-color-antibodies-ruo/pe-mouse-anti-human-cd107a.555801
CD56 - clone NCAM16.2, BV786 (BD Biosciences Cat# 564058): https://www.bdbiosciences.com/en-us/products/reagents/flow-cytometry-reagents/research-reagents/single-color-antibodies-ruo/bv786-mouse-anti-human-cd56.564058
Ki-67 - clone SolA15, PE-Cy5 (ThermoFisher Scientific Cat# 15-5698-82): https://www.thermofisher.com/antibody/product/Ki-67-Antibody-clone-SolA15-Monoclonal/15-5698-82
anti-Rabbit HRP secondary antibody (Leica Biosystems, Catalog # DS9800): https://shop.leicabiosystems.com/us/ihc-ish/detection-systems/pid-bond-polymer-refine-detection

Eukaryotic cell lines

Policy information about cell lines and Sex and Gender in Research

Cell line source(s)

H29 cells were developed in the Richard C. Mulligan lab and have been previously described (PMID: 8876147). The K562 cell line was purchased from ATCC (CCL-243).

Authentication

H29 cells were strictly maintained with the selection antibiotics: G418 (gag/pol selection) and puromycin (VSV-G selection) to ensure maintenance of retroviral protein-expressing plasmids (no additional authentication). STR profiling was performed to authenticate the K562 cell line.

Mycoplasma contamination

Cell lines were regularly tested using MycoAlert Mycoplasma Detection Kit (Lonza). None of the cell lines used in this study tested positive for Mycoplasma.

Commonly misidentified lines (See ICLAC register)	No commonly misidentified lines were used in this study.

Clinical data

Policy information about clinical studies

All manuscripts should comply with the ICMJE guidelines for publication of clinical research and a completed CONSORT checklist must be included with all submissions.

Clinical trial registration	NCT04161755
Study protocol	Updated study protocol is available (Supplementary File 1).
Data collection	Patients were enrolled from December 2019 to August 2021. Data were collected at MSK during and beyond enrollment period.
Outcomes	Primary endpoint was safety and was assessed using a predefined number of grade 3 adverse events due to atezolizumab and autogene cevumeran per number of patients enrolled (Table 1 in manuscript). Secondary endpoints were 18-month recurrence free survival (RFS) and overall survival (OS). We defined recurrence as new lesions by RECIST 1.1, and RFS from either the date of surgery (RFS), or from the date of the last autogene cevumeran priming dose (landmark RFS) to the date of recurrence or death, whichever occurred first. We censored patients without events at the last known date they were recurrence-free. We defined OS from the date of surgery to the date of death. As exploratory endpoints, we measured immune response and feasibility as actual vs. benchmarked treatment times. Data cut-off was April 1, 2022, extending the median follow-up beyond the prespecified 18-month RFS secondary endpoint.

Flow Cytometry

Plots

Confirm that:

☒ The axis labels state the marker and fluorochrome used (e.g. CD4-FITC).

☒ The axis scales are clearly visible. Include numbers along axes only for bottom left plot of group (a 'group' is an analysis of identical markers).

☒ All plots are contour plots with outliers or pseudocolor plots.

☒ A numerical value for number of cells or percentage (with statistics) is provided.

Methodology

Sample preparation	We purified patient peripheral blood mononuclear cells (PBMCs) from blood samples by density centrifugation over Ficoll-Paque Plus (GE Healthcare, IL, USA). We purified healthy donor PBMCs from buffy coats (New York Blood Center, NY, USA) and isolated T cells using a Pan-T cell isolation kit (Miltenyi Biotech, Germany). We let patient PBMCs rest overnight at 37°C and 5% CO2 before staining. We activated T cells with CD3/CD28 beads (Thermo Fisher, MA, USA) with IL-7 (3000 IU/ml) and IL-15 (100 IU/ml) (Miltenyi Biotec, Germany), and transduced T cells on day 2 post activation. We defined TCR-transduced CD8+ T cells as live, CD3+, CD8+, mTCR+ cells. We stained cells using antibody cocktails in the dark at 4°C, washed, and analyzed. To examine expression of intracellular markers, we surface-stained, fixed, permeabilized, and stained the cells for intracellular proteins using the Fixation and Permeabilization Buffer Kit as per the manufacturer's recommendations (Invitrogen, MA, USA). Full details are provided in the Methods.
Instrument	Flow cytometry was performed on an LSR Fortessa (BD Biosciences; Catalog# 647177; Serial# H64717700135).
Software	Data were analyzed using FlowJo Software (version 10, Tree Star).
Cell population abundance	Representative cell abundance is indicated in Extended Data Figure 4a. Due to the scarcity of the samples, we were not able to confirm the purity of the populations within the post-sorting fractions.
Gating strategy	Relevant gating strategies are indicated in Extended Data Figure 4a.<br><br>We gated lymphocytes based on size and complexity gating (SSC-A vs FSC-A). We considered events with high SSC-W and FSC-W and normal SSC-H and FSC-W, respectively, as doublets and excluded them from the analysis. We identified dead cells as positive for Fixable Viability Dye 520 (top) or DAPI (bottom) and excluded them from the analysis. We identified T cells by the expression of CD3 and lack of expression of CD56. We identified degranulating T cells (top) by the expression of the surface marker CD107a, and we set the gate using mock-stimulated T cells pulsed with DMSO (no peptides were added). We identified T cells expressing the transduced TCRs (bottom)by the expression of mTCR using untransduced T cells to set the gate. Then, we determined T cell activation by the expression of 4-1BB using untransduced T cells and transduced T cells pulsed with an irrelevant peptide to set the gate.

☒ Tick this box to confirm that a figure exemplifying the gating strategy is provided in the Supplementary Information.
