# Supplementary material for: Personalized RNA neoantigen vaccines stimulate T cells in pancreatic cancer
Source: Nature. 2023 May 10;618(7963):144–50. doi: 10.1038/s41586-023-06063-y (PMC10171177; doi:10.1038/s41586-023-06063-y)
Supplement: Supplementary file 3 — Supplementary Tables 1–4 [file 41586_2023_6063_MOESM3_ESM.pdf]

Supplementary Table 1. T cell clones at final assessment

Immune responders (*n* = 8)		
Characteristic		
Final assessment (weeks after surgery)		
	Median (range)	66.2 (53.4 - 104.6)
	Mean ± SD	69.61 +/- 15.47
Final assessment (weeks after first vaccination)		
	Median (range)	57.0 (44.3 - 96.0)
	Mean ± SD	59.89 +/- 15.90
Number of vaccine-expanded clones		
	Median (range)	7 (2 - 23)
	Mean ± SD	8.38 ± 7.19
Percentage of vaccine-expanded clones		
	Median (range)	89.52 (50 - 100)
	Mean ± SD	86.4 ± 16.85
Percentage of all T cells		
	Median (range)	0.34 (0.03 - 2.55)
	Mean ± SD	1.27 ± 1.58

SD: standard deviation

Supplementary Table 2. Genetic characteristics in all evaluable patients

Safety evaluable + biomarker evaluable patients (*n* = 17*)		
Characteristic		
Germline mutations (no.)<br>(RAD50, BLM, PALB2, APC, ATM)		
	Median (range)	0 (0 - 2)
	Mean ± SD	0.3 ± 0.6
Somatic mutations (no.)†		
	Median (range)	3 (1 - 6)
	Mean ± SD	3.2 ± 1.5
Copy number alterations (no.)		
	Median (range)	0 (0 - 8)
	Mean ± SD	0.9 ± 2.3
Somatic mutations + copy number (no.)		
	Median (range)	3 (1 - 12)
	Mean ± SD	4.1 ± 3.2

SD: standard deviation

*: Two patients did not have testing

†: Somatic genes by MSK-IMPACT (*n* = 468 genes)

Supplementary Table 3. Genetic characteristics in all responders vs. non-responders

Biomarker evaluable patients				
Characteristic		Responders (*n* = 6*)	Non-responders (*n* = 8)	*P* value
Germline mutations (no.) (RAD50, BLM, PALB2, APC, ATM)				
	Median (range)	0 (0 - 1)	0 (0 - 1)	>0.99
	Mean ± SD	0.2 ± 0.4	0.2 ± 0.5	
Somatic mutations (no.)[†]				
	Median (range)	4 (2 - 6)	3 (1 - 5)	0.3
	Mean ± SD	4.0 ± 1.5	3.0 ± 1.3	
Copy number alterations (no.)				
	Median (range)	0 (0 - 6)	0 (0 - 8)	0.9
	Mean ± SD	1.2 ± 2.4	1.1 ± 2.8	
Somatic mutations + copy number				
	Median (range)	4 (2 - 11)	3.5 (1 - 12)	0.9
	Mean ± SD	5.2 ± 3.4	4.1 ± 3.4	
Driver genes, *n* (%)				
	KRAS	6 (100)	8 (100)	NA
	TP53	4 (67)	7 (87.5)	0.5
	CDKN2A	2(33)	2 (25)	>0.99
	SMAD4	0 (0)	0 (0)	
Other somatic mutations, *n* (%)				
	ARID1A	1 (16.6)	1 (12.5)	>0.99
	AR	0 (0)	1 (12.5)	>0.99
	PDGFRB	1 (16.6)	1 (12.5)	>0.99
	BRCA2	1 (16.6)	0 (0)	0.4
	TGFBR2	1 (16.6)	0 (0)	0.4
	TRAF7	1 (16.6)	0 (0)	0.4
	SMAD3	1 (16.6)	0 (0)	0.4
	PHF6	1 (16.6)	0 (0)	0.4
	MTOR	1 (16.6)	0 (0)	0.4
	MED12	0 (0)	1 (12.5)	>0.99
	KMT2A	0 (0)	1 (12.5)	>0.99
	MAP2K4	1 (16.6)	0 (0)	0.4
	CTNNB1	0 (0)	1 (12.5)	>0.99

SD: standard deviation; *P* values by two-tailed Mann Whitney or Fisher's Exact tests.
*: Two responders did not have testing
†: Somatic genes by MSK-IMPACT (*n* = 468 genes)

Supplementary Table 4. Germline gene targets in MSK-IMPACT

ALK (NM_004304)	APC (NM_000038)	ATM (NM_000051)	BAP1 (NM_004656)
BARD1 (NM_000465)	BLM (NM_000057)	BMPR1A (NM_004329)	BRCA1 (NM_007294)
BRCA2 (NM_000059)	BRIP1 (NM_032043)	CDC73 (NM_024529)	CDH1 (NM_004360)
CDK4 (NM_000075)	CDKN2A (NM_058195)	CDKN2A (NM_000077)	CEBPA (NM_004364)
CHEK2 (NM_007194)	DICER1 (NM_177438)	EGFR (NM_005228)	EPCAM (NM_002354; exons8-9 deletion)
ERBB2 (NM_004448)	ERCC3 (NM_000122)	ETV6 (NM_001987)	FAM175A (NM_139076)
FANCA (NM_000135)	FANCC (NM_000136)	FH (NM_000143)	FLCN (NM_144997)
GATA2 (NM_032638)	HOXB13 (NM_006361; c.251G>A p.Gly84Glu)	HRAS (NM_005343)	KIT (NM_000222)
KRAS (NM_004985)	MAX (NM_002382)	MEN1 (NM_130799)	MET (NM_001127500)
MITF (NM_000248; c.952G>A p.Glu318Lys)	MLH1 (NM_000249)	MRE11A (NM_005591)	MSH2 (NM_000251)
MSH3 (NM_002439)	MSH6 (NM_000179)	MUTYH (NM_001128425)	NBN (NM_002485)
NF1 (NM_000267)	NF2 (NM_000268)	NRAS (NM_002524)	NTHL1 (NM_002528)
PALB2 (NM_024675)	PAX5 (NM_016734)	PDGFRA (NM_006206)	PHOX2B (NM_003924)
PMS2 (NM_000535)	POLD1 (NM_002691; (amino acids197-562))	POLE (NM_006231; (amino acids 268-491))	PTCH1 (NM_000264)
PTEN (NM_000314)	RAD50 (NM_005732)	RAD51 (NM_002875)	RAD51B (NM_133509)
RAD51C (NM_058216)	RAD51D (NM_002878)	RB1 (NM_000321)	RECQL (NM_002907)
RECQL4 (NM_004260)	RET (NM_020975)	RTEL1 (NM_032957)	RUNX1 (NM_001754)
SDHA (NM_004168)	SDHAF2 (NM_017841)	SDHB (NM_003000)	SDHC (NM_003001)
SDHD (NM_003002)	SMAD3 (NM_005902)	SMAD4 (NM_005359)	SMARCA4 (NM_001128849)
SMARCB1 (NM_003073)	STK11 (NM_000455)	SUFU (NM_016169)	TERT (NM_198253)
TGFBR1 (NM_004612)	TGFBR2 (NM_003242)	TMEM127 (NM_017849)	TP53 (NM_000546)
TSC1 (NM_000368)	TSC2 (NM_000548)	VHL (NM_000551)	WT1 (NM_024426)

Supplementary Table 5. mRNA vaccine neoantigen characteristics

This table is provided in Excel format file, and contains a comprehensive list of neoantigen targets used in vaccines.
